# Supplementary material for: Early Life Interventions for Childhood Growth and Development in Tanzania (ELICIT): a protocol for a randomised factorial, double-blind, placebo-controlled trial of azithromycin, nitazoxanide and nicotinamide
Source: BMJ Open. 2018 Jul 7;8(7):e021817. doi: 10.1136/bmjopen-2018-021817 (PMC6042604; doi:10.1136/bmjopen-2018-021817)
Supplement: Author's manuscript [file bmjopen-2018-021817.draft_revisions.pdf]

BMJ Open is committed to open peer review. As part of this commitment we make the peer review history of every article we publish publicly available.

When an article is published we post the peer reviewers' comments and the authors' responses online. We also post the versions of the paper that were used during peer review. These are the versions that the peer review comments apply to.

The versions of the paper that follow are the versions that were submitted during the peer review process. They are not the versions of record or the final published versions. They should not be cited or distributed as the published version of this manuscript.

BMJ Open is an open access journal and the full, final, typeset and author-corrected version of record of the manuscript is available on our site with no access controls, subscription charges or pay-per-view fees (http://bmjopen.bmj.com).

If you have any questions on BMJ Open's open peer review process please email info.bmjopen@bmj.com

BMJ Open

Early Life Interventions for Childhood Growth and Development in Tanzania (ELICIT): A Protocol for a Randomized Factorial, Double-Blind, Placebo-Controlled Trial of Azithromycin, Nitazoxanide and Nicotinamide

Journal:	*BMJ Open*
Manuscript ID	bmjopen-2018-021817
Article Type:	Protocol
Date Submitted by the Author:	19-Jan-2018

Complete List of Authors:	DeBoer, Mark; University of Virginia, Pediatrics<br>Platts-Mills, James; University of Virginia, Medicine<br>Scharf, Rebecca; University of Virginia Children's Hospital, Pediatrics<br>McDermid, Joann; University of Virginia, Medicine<br>Wanjuhi, Anne; University of Virginia, Global Health<br>Gratz, Jean; University of Virginia, Medicine<br>Svensen, Erling; Haukeland Universitetssjukehus<br>Swann, Jon; Imperial College London<br>Donowitz, Jeffrey; University of Virginia, Medicine<br>Jatosh, Samwel; Haydom Lutheran Hospital, Haydom Global Health Research Center<br>Houpt, Eric; University of Virginia,<br>Mduma, Estomih ; Haydom Lutheran Hospital

Keywords:	growth, stunting, development, antibiotics, niacin, Tanzania

For peer review only

SCHOLARONE™
Manuscripts

Early Life Interventions for Childhood Growth and Development in Tanzania (ELICIT): A Protocol for a Randomized Factorial, Double-Blind, Placebo-Controlled Trial of Azithromycin, Nitazoxanide and Nicotinamide

Mark D. DeBoer MD, MSc, MCR[1,7]; James A. Platts-Mills MD[2]; Rebecca J. Scharf MD, MPH[1]; Joann M. McDermid Ph.D, RD[2]; Anne Wanjuhi MPH[1]; Jean Gratz MS[2]; Erling Svensen Ph.D[3]; Jon R. Swann Ph.D[4]; Jeffrey R. Donowitz MD[5]; Samwel Jatosh BS[6]; Eric R. Houpt MD[2]; Estomih Mduma MPH[6]

1. Department of Pediatrics, University of Virginia, Charlottesville, VA, USA;
2. Center for Global Health, Department of Medicine, University of Virginia, Charlottesville, VA, USA;
3. Department of Global Health and Primary Care, University of Bergen, Bergen, Norway;
4. Imperial College of London, London, UK;
5. Division of Infectious Disease, Children's Hospital of Richmond at Virginia Commonwealth University
6. Haydom Global Health Research Centre, Haydom, Tanzania;
7. Corresponding Author:

Division of Pediatric Endocrinology
University of Virginia School of Medicine
P.O. Box 800386
Charlottesville, VA 22908
Phone: 434-924-5956
Fax: 434-924-9181
Email: deboer@virginia.edu

For peer review only - http://bmjopen.bmj.com/site/about/guidelines.xhtml

ABSTRACT

Introduction: In many developing areas in the world, a high burden of enteric pathogens in early childhood are associated with growth deficits. The tryptophan-kynurenine-niacin pathway has been linked to the enteric inflammatory response to intestinal infections. However, it is not known in these settings whether scheduled antimicrobial intervention to reduce subclinical enteric pathogen carriage or repletion of the tryptophan-kynurenine-niacin pathway improves linear growth and development.

Methods and analysis: We are conducting a randomized, placebo-controlled, factorial intervention trial in the rural setting of Haydom, Tanzania. We are recruiting 1188 children within the first 14 days of life, who will be randomized in a 2 x 2 factorial design to administration of antimicrobials (azithromycin and nitazoxanide, randomized together) and nicotinamide. The nicotinamide is administered as a daily oral dose, which for breast-feeding children aged 0-6 months is given to the mother and for children aged 6-18 months is given to the child directly. Azithromycin is given to the child as a single oral dose at months 6, 9, 12, and 15; nitazoxanide is given as a 3-day course at months 12 and 15. Mother/child pairs are followed via monthly in-home visits. The primary outcome is the child's length-for-age Z-score at 18 months. Secondary outcomes for the child include additional anthropometry measures; stool pathogen burden and bacterial microbiome; systemic and enteric inflammation; blood metabolomics, growth factors, inflammation and nutrition; hydrogen breath assessment to estimate small-intestinal bacterial overgrowth; and assessment of cognitive development. Secondary outcomes for the mother include breast milk content of nicotinamide, other vitamins and amino acids; blood measures of tryptophan-kynurenine-niacin pathway; and stool pathogens.

Ethics and dissemination: This trial has been approved by the Tanzanian National Institute for Medical Research, the Tanzanian Food and Drug Administration and the University of Virginia Institutional Review Board.

Trial registration number: ClinicalTrials.gov #NCT03268902.

For peer review only - http://bmjopen.bmj.com/site/about/guidelines.xhtml

Key words: growth, stunting, development, antibiotics, niacin, Tanzania

Protocol version: 5.0, 4 December 2017

Protocol Sponsor: Haydom Lutheran Hospital, Haydom, Manyara, Tanzania

For peer review only

For peer review only - http://bmjopen.bmj.com/site/about/guidelines.xhtml

STRENGTHS AND LIMITATIONS OF THIS STUDY

- This study assesses the efficacy of two different intervention approaches targeting early childhood growth and development in a rural site in sub-Saharan Africa with a high degree of both enteric pathogen carriage and stunting.
- This study assesses intervention with nicotinamide, targeting the tryptophan-kynurenine-niacin pathway, which may have important roles in intestinal bacterial content and immune and metabolic response.
- In addition to the primary outcome of length-for-age Z-score, this study measures extensive secondary outcomes related to infection, nutrition, metabolism and cognitive development.
- Due to sample size constraints, one limitation of the study is that two different antimicrobial interventions (azithromycin and nitazoxanide) are randomized together, limiting the ability to determine any individual contribution of either of these to study outcomes.

For peer review only

INTRODUCTION

Childhood growth and thriving remain sub-optimal in low-resource settings worldwide, underscoring the need to better understand underlying etiologies and to identify interventions toward improving outcomes.[1 2] The Etiology, Risk Factors, and Interactions of Enteric Infections and Malnutrition and the Consequences for Child Health (MAL-ED) study evaluated correlates of childhood growth and cognitive development in 8 low- to middle-income countries (LMIC) worldwide, including the region around Haydom, Tanzania.[3 4] Haydom is in a rural, semi-arid area with a high degree of poverty. Children in Haydom followed in MAL-ED experienced a 70% rate of stunting by 18 months of age.[5] Data from MAL-ED also showed that linear growth deficits correlated with lower scores for cognitive development,[6] potentially through shared barriers to optimal growth and brain development.[1 7]

Antimicrobials to improve childhood growth

In MAL-ED, children in the Haydom site had a high burden of infection with intestinal pathogens starting early in life. High carriage of enteropathogens, even in the absence of diarrhea, was associated with a 1.36 increased odds of having lower length for age.[3] As for the specific enteropathogens, among the most prevalent pathogens at age 6 months were enteroaggregative *E.coli* (48%) and *Campylobacter* (27%),[8] each of which were associated with a 0.85 cm and 0.83 cm, respectively, shortfall in height attainment.[9] Both of these infections are treatable with azithromycin. Other highly prevalent pathogens by age 6 months in the MAL-ED study included *Giardia* (22%) and *Cryptosporidium* (6%), and early *Giardia* infection was also associated with growth shortfalls.[10] Both of these protozoal infections are treatable with nitazoxanide. These observations raise questions as to whether such antibiotics may be useful in ameliorating growth deficits.

A meta-analysis of 10 randomized controlled trials showed that antibiotic use increased height by 0.04 cm/month.[11] Another study demonstrated that randomized administration of

For peer review only - http://bmjopen.bmj.com/site/about/guidelines.xhtml

azithromycin to asymptomatic young children in Ethiopia was associated with a halving of mortality,[12] suggesting overall effects of suppressing bacterial pathogens in areas with high rates of infectious disease. However results from individual studies of azithromycin mass drug-administration on growth have not shown consistent results.[13] Among children with cryptosporidial infection in Zambia, treatment with nitazoxanide resulted in reduced mortality, while linear growth was not assessed.[14] Therefore, the role of these antimicrobials for linear growth remains unclear.

Nicotinamide to improve childhood growth

Another potential means of improving growth is by targeting the tryptophan-kynurenine-niacin pathway (Figure 1). In this pathway, dietary tryptophan is absorbed and then metabolized by indoleamine 2,3-dioxygenase (IDO) to kynurenine and later to nicotinic acid, a key precursor to NAD+. The importance of this pathway has been supported by recent work identifying mutations in the NAD+ pathway as a cause of birth defects in humans; in animal models with these mutations, birth defects were prevented via supplementation with niacin.[15] Deficiencies in this pathway have been noted in areas where diets are heavily maize-based, leading to pellagra. Infants at the Haydom site in MAL-ED were noted to have inadequate dietary intake of multiple vitamins[16]; while deficiency in niacin was not assessed, individuals in the region around Haydom eat a diet based heavily on maize, with potentially low bioavailability of tryptophan. Basic science models have demonstrated that tryptophan deficiency is associated with reduced growth velocity.[17]

Abnormalities in the tryptophan-kynurenine-niacin pathway are also associated with intestinal inflammation, including during response to intestinal pathogens.[18 19] In animal models, an inability to transport tryptophan into enterocytes results in diarrhea, while provision of nicotinamide (a form of niacin) partly restores function of mTOR in enterocytes, increasing secretion of antimicrobial peptides and reducing diarrhea,[18 19] suggesting effects on diarrheal

For peer review only - http://bmjopen.bmj.com/site/about/guidelines.xhtml

pathogens. IDO activity is increased during inflammation, including in inflammatory bowel disease[20 21] and during infections;[22] this appears to be a maladaptive in most cases, as kynurenine has potential immunosuppressive effects.[23 24] In MAL-ED, children at the Haydom site with higher ratios of kynurenine:tryptophan were noted to have poorer linear growth.[24] Given that 1) children in the Haydom area have a maize-based diet low in tryptophan, a key precursor to niacin/nicotinamide and NAD+, 2) preclinical studies demonstrate improvements in antimicrobial peptides and reduced diarrhea with nicotinamide supplementation, 3) an increase in nicotinamide as a precursor of NAD+ should cause reduced need for the tryptophan-kynurenine-niacin pathway, reducing activity of IDO and thereby reducing kynurenine, and 4) higher kynurenine:tryptophan is associated with poor growth, we reasoned that intervention with nicotinamide among children in this region would improve linear growth.

Study hypotheses

We hypothesized that among children with a high degree of stunting and enteric pathogen burden in this region, intervention with 1) antimicrobials and/or 2) nicotinamide would increase linear growth. We further set out to interrogate the potential mechanisms behind these effects. In the case of antimicrobial intervention, we hypothesize that intervention with azithromycin and nitazoxanide (relative to placebo) would result in a decrease in enteropathogen burden that would be associated with an increase in growth. In the case of nicotinamide (relative to placebo), we hypothesize that intervention will result in a reduction in the kynurenine:tryptophan ratio, an increase in availability of nicotinamide for NAD+ synthesis and associated increases in growth.

OBJECTIVE

The primary objectives of this randomized factorial, double-blind, placebo-controlled trial are to determine if interventions with 1) antimicrobials and 2) nicotinamide are associated with

For peer review only - http://bmjopen.bmj.com/site/about/guidelines.xhtml

increased length-for-age z-score (LAZ, relative to placebo-treated children) by age 18 months. Specifically, the antimicrobial intervention consists of azithromycin administered as a single dose at age 6, 9, 12, and 15 months and nitazoxanide as a 3-day course at age 12 and 15 months (with both antimicrobials randomized together). The nicotinamide intervention consists of nicotinamide 250 mg daily administered daily by mouth to the child's mother during breast feeding 0-6 months (and thus passed on to the child via breast milk[25]) and then 100 mg administered daily by mouth to the child during months 6-18. These interventions are delivered in a factorial manner such that children receive both interventions, either intervention alone or neither intervention.

Because multiple prior large-scale studies of other preparations of vitamins and antibiotics have failed to show effects on linear growth, we have multiple secondary outcomes to interrogate additional physiological effects of the interventions—potentially helping to identify underlying mechanisms. These secondary outcomes include measures of inflammation, intestinal bacterial content (pathogens and otherwise), and measures of metabolism, development and health.

METHODS AND ANALYSIS (Standard Protocol Items for Randomized Trials)

Eligibility

Participants are eligible if the mother is ≥18 years and the child is age ≤14 days and was born in the Haydom catchment area (defined for study purposes as 25 km radius of Haydom Lutheran Hospital).

Exclusion criteria are as follows: maternal inability to adhere to the protocol, multiple gestation, significant birth defect or neonatal illness, weight <1,500 g at enrollment (age ≤ 14 days), lack of intent to breastfeed infant and plan to move from area within the next 18 months.

For peer review only - http://bmjopen.bmj.com/site/about/guidelines.xhtml

Recruitment

Recruitment occurs via survey by field teams of surrounding villages and via input from local community healthcare workers. Pregnant women are identified and informed regarding the study. Pregnant women in the third trimester may provide informed consent for the study but are not enrolled until the initial assessment visit of the infant to determine eligibility (which includes ascertainment of infant weight). If informed consent is not obtained during pregnancy, it is obtained at enrollment.

Enrollment

Infant/mother pairs are enrolled during the initial study visit to their homes between birth and day of life 14, after eligibility criteria are confirmed, including measurement of infant weight which should be 1.5 or above. Informed consent is provided at this time, if it was not obtained during pregnancy. Eligibility (besides current weight) is confirmed and the mother/child pair is randomized in the study.

Randomization

Randomization occurs at the time of enrollment. Block randomization was used in groups of eight for the four intervention combinations (antimicrobial + nicotinamide, antimicrobial alone, nicotinamide alone, all placebo). Study teams assign participant study number prior to the initial study visit.

Intervention

As a factorial study design, the two intervention domains (antimicrobial and nicotinamide) are randomized separately. The antimicrobial intervention consists of two medications that are randomized together: azithromycin and nitazoxanide (vs. placebos of both). Azithromycin 20 mg/kg (vs. placebo) (both manufactured by Universal Corp, Kenya) is

For peer review only

For peer review only - http://bmjopen.bmj.com/site/about/guidelines.xhtml

administered by study personnel at months 6, 9, 12 and 15 as a single dose of azithromycin (200 mg/5 mL suspension by mouth). The nitazoxanide (vs. placebo) (both manufactured by Romark, Florida, USA) is administered as a course of nitazoxanide (100 mg/5 mL) 100 mg twice daily for three days. The first of these nitazoxanide doses is administered by the study personnel and the remaining doses are administered at home by the child's family. Families are asked at a subsequent visit about whether they completed the course of nitazoxanide.

The nicotinamide (vs. placebo) (both manufactured by Vita-gen, New York, USA) is delivered as a daily dose throughout the study. During the first 6 months of life, this is delivered to the child via breastmilk. Mothers take nicotinamide 250 mg tablets, one tablet by mouth daily. Prior studies have demonstrated that nicotinamide is present in breast milk in quantities that are associated with serum levels.[25] Haydom-specific data from MAL-ED demonstrated a high prevalence (96%) of breast feeding until children were 6 months old.[5]

From months 6-18 the child receives sachets of nicotinamide 100 mg by mouth daily. Mothers are instructed to mix the nicotinamide powder with a small volume of age-appropriate food (e.g. infant cereal) to give to the child.

For both the maternal nicotinamide pills and the children's nicotinamide sachets, doses are given as a 60-day supply every two months. When a new supply is provided, the family is asked for the remainder of doses not consumed the prior 60 days. These unused doses are counted and recorded by pharmacy staff to determine adherence.

Data collection

Questionnaires

Study staff use standardized questionnaires designed for the study, in many cases adapted from questionnaires used in the MAL-ED study. These include questions asked at each monthly visit regarding breastfeeding, use of complementary foods, childhood illness and treatment (including whether an antibiotic was used and if so which one), types of food sources

For peer review only - http://bmjopen.bmj.com/site/about/guidelines.xhtml

For peer review only

given, and food insecurity. Additional questionnaires are asked at other specific time periods, including aspects of the child's home environment (completed at the first month's visit) and the vaccines that the child has received (completed at the 12-month visit).

Anthropometry measures

All field team members are trained in the accurate measurement of anthropometry indices. The child's weight is measured at the initial visit and at each monthly visit through age 18 months. The child's length and head circumference are measured at the initial visit and every three visits thereafter through 18 month. The mother's weight and height are measured at month 10-11. All weights are measured using a digital scale that undergoes quality assessments weekly. Infants are weighed on an infant tray with the infant nude or wearing a dry diaper or standing, when able. Lengths are measured by two field team members with the child lying flat on a measuring board with a fixed, perpendicular board against which the head is placed and a second adjustable perpendicular board is placed against the feet. Two measurements are obtained; if these are within 2 mm, the average of the two is used; if these are not within 2 mm, a third measurement is obtained and the average of the two closest measurements is used. Head circumference is measured by placing a non-distensible tape around the child's head just above the eyebrows, above the ears, and around the biggest part of the back of the head. Maternal weight is measured using a standing scale. Maternal height is measured using a portable stadiometer. For quality assurance, 5% of infant weights, lengths, and head circumferences are measured by a supervisor skilled in measurement to compare to the field team's measurement.

Developmental assessments

To assess maternal-child interaction, an important predictor of early childhood development and health,[26 27] The Observation of Maternal Child Interaction (OMCI)[28]

For peer review only - http://bmjopen.bmj.com/site/about/guidelines.xhtml

assessment is collected at 12 months of age. This observation includes a 5 minute observation of mother and child interacting over a picture book, with ratings given for positive affect, verbal statements, sensitivity, scaffolding, language stimulation, focus and mutual enjoyment, among others.

At 18 months, developmental assessment is performed by trained assessors using two standardized scores, the Malawi Developmental Assessment Tool (MDAT)[29] and the Bayley Scales of Infant and Toddler Development, cognitive assessment (Bayley).[30] The MDAT is a validated score that tests multiple childhood development domains, including gross motor, fine motor, language and social development that has been validated in this age range in Malawi and intended for use in rural sub-Saharan settings. Briefly, this assessment was designed to be a culturally-relevant method for examining child development across multiple domains using objects and tools typically available in rural or lower-resource settings. Both direct assessment and caregiver report of skills go into the score.

The Bayley cognitive assessment involves a series of tasks presented to the infant, which assess for play, reasoning, and mental representation. This is a commonly used assessment for early childhood and has been used in many research sites around the world.[6 31]

Specimen collection

Breast milk (months 1 and 5): Participating mothers express a maximal volume of breast milk from one breast (up to 40 mL), using hand expression under low-light conditions. Breast milk will be collected into foil-covered sterile containers and transported on ice to the central laboratory center within 8 hours for archiving of whole milk at -80 degrees.

Blood (months 12 and 18; in a subset months 2 and 8): Blood is drawn from an experienced phlebotomy team using a butterfly needle and syringe and placed in micro-containers for transport to the laboratory for processing into serum and plasma and storage at -80 degrees. In

For peer review only - http://bmjopen.bmj.com/site/about/guidelines.xhtml

the case of laboratory studies to assess the safety of nicotinamide administration, a whole blood specimen is immediately tested in the clinical laboratory at Haydom Lutheran Hospital for complete blood count (CBC).

Stool (months 6, 9, 12, 15, and 18): Stool is collected in diapers that are provided just prior to the stool collection date. Mothers or caregivers are instructed to collect approximately 3–4 spoonfuls of stool and place it within 1 hour into a labeled stool container, seal in a plastic bag, and place inside a transport box with cold packs. The stool collection will be timed prior to a planned study visit, such that the field-worker can collect the sample and the laboratory process the specimen shortly thereafter. Stool samples are then stored at -80 degrees. A subset of specimens, prior to freezing will be cultured for E.coli and screened for antibiotic resistance (AMR).

Urine (months 6, 12, and 18): Urine is collected by placing a bag over the child's genitalia at the beginning of a study visit. The child is given adequate hydration, via breast feeding if possible. Collected urine is then transferred in a sterile container that is stored on ice prior to transport to the research site and then stored at -80 degrees.

Saliva (maternal and infant at month 12): Maternal and child saliva is collected into a vial containing storage reagent and stored at -80 degrees for genomic assessment.

Breath (subset of participants at months 6, 9 and 12): Glucose hydrogen breath testing is performed in a subset of children as an assessment of small intestine bacterial overgrowth (SIBO),[32] which has been associated with intestinal inflammation and linear growth shortfall.[33] Children are fasted for 2 hours prior to the initiation of the breath test. They are then given 1 g/kg actual body weight of a glucose dissolved in a 1 g/5ml solution. Following ingestion of the

For peer review only - http://bmjopen.bmj.com/site/about/guidelines.xhtml

standardized glucose solution, participants have breath collected via the Quintron pediatric breath collection bag system every 20 minutes for 2 hours. This non-invasive procedure involves the child breathing into an anesthesia mask connected to a one-way flutter valve and collection bag. Breath samples are extracted from the collection bag using a syringe and stopcock and placed into specialized vacutainer glass vials. These vials are then transported to our research facility at Haydom Lutheran Hospital for assessment.

Laboratory procedures and storage

Upon receipt of the samples listed above, the laboratory team at the research site prepares aliquots of samples for storage at -80 degrees for local testing or shipping to additional testing facilities as shown below.

Breast milk

Rationale

During the first 6 months of participation, the only means of the child receiving intervention is through maternal delivery of nicotinamide in breast milk. However, nicotinamide *in vivo* is both derived from tryptophan and converted to multiple other vitamers.[34] As such, we will assess content of nicotinamide, vitamers and tryptophan in breast milk at two time points during the intervention.

Maternal breast milk samples collected at months 1 and 5 will be tested at the United States Department of Agriculture laboratory at the University of California – Davis. Planned LC-MS analyses includes nicotinamide, thiamin, riboflavin, flavin adenine dinucleotide, pyridoxal, pyridoxine, biotin and pantothenic acid concentrations, and on a random subset (n=600 samples) niacin vitamer, and free and total tryptophan concentrations.

Blood

For peer review only - http://bmjopen.bmj.com/site/about/guidelines.xhtml

Rationale

Blood testing will include assessment of multiple measures both to verify safety of the interventions and interrogate the effects of the intervention on physiological processes related to linear growth. Safety assessment includes testing of general tests of health with basic metabolic panel, liver function and enzyme testing, and complete blood count. This will assess for differences between intervention groups in measures of kidney, liver and hematopoetic health. Additional tests to interrogate the physiologic effects of the intervention include assessment of metabolic effects (using metabolomics) and assessment of levels of factor related to growth (insulin-like growth factor-1 [IGF-1] and collagen X).

<u>Complete blood count (CBC)</u>: To assess general safety of the nicotinamide dosing in the first 100 infants at months 2 and 8. CBC is performed at Haydom Lutheran Hospital clinical on a Swelab Alfa hematology analyzer (Boule Diagnositics, Stagna, Sweden; this machine has quality control assessed daily).

<u>Basic metabolic panel (BMP), aminotransferases and bilirubin</u>: To assess general safety of the nicotinamide dosing in the first 100 infants at months 2 and 8, additional laboratory measures will be performed on serum samples at the Kilimanjaro Christian Medical Center. BMP, bilirubin, alanine aminotransferase (ALT) and aspartate aminotransferase (AST) will be assessed using a Cobas Integra 400 Plus biochemistry analyzer (Roche Diagnostics, Germany), according to the manufacturer's instructions.[35]

<u>Insulin-like growth factor-1 (IGF-1) and high-sensitivity C-reactive protein (hsCRP)</u>: To interrogate these interventions for changes in growth factor and low-grade systemic inflammation, respectively, IGF-1 and hsCRP will be tested at 12 and 18 months at the UVa Center for Research in Reproduction Ligand Core Laboratory using an Immulite 2000.

For peer review only - http://bmjopen.bmj.com/site/about/guidelines.xhtml

Collagen X: We will assess for intervention-associated changes in collagen X (a biomarker of rapid skeletal growth) using an ELISA developed at Shriners Hospital for Children in Portland, OR, and Oregon Health and Science University.[36]

Metabolomics: The tryptophan-kynurenine-niacin pathway will be assessed at 12 and 18 months via metabolomics analyses of serum and plasma using ^{1}H nuclear magnetic resonance (NMR) spectroscopy and ultra-performance liquid chromatography-mass spectrometry (UPLC-MS) at Imperial College, London. Assessments include the following: 1) metabolites of the tryptophan-kynurenine-niacin pathway to assess for (among other items) changes in the tryptophan:kynurenine ratio in the setting of nicotinamide treatment;[24] 2) plasma bile salts, which have implications for risk of infection;[37] 3) untargeted metabolomics analysis, providing information on the global metabolic status of the individual including pathways related to the metabolic activity of the gut microbiota and their biochemical interactions with the host;[38] and untargeted UPLC-MS based lipidomics to assess for differences in lipid metabolism by nutritional status.

Stool

Stool testing will include assessment of the following:

Microbiome: Stool bacterial content will be assessed at in a subset of 200 participants at the University of Maryland. Total stool DNA will be isolated by bead beating method. A library will be created each specimen. The libraries will be multiplexed and sequenced by Illumina HiSeq. The resulting reads will be cleaned, binned by specimen ID, separated in operational taxonomic units (OTUs) by DNAclust[39] and compared using metagenomeseq. Metagenomeseq normalizes the data and introduces a correction factor to account for under-sampling of OTUs.

For peer review only - http://bmjopen.bmj.com/site/about/guidelines.xhtml

The normalized data will be compared using odds ratios and multiple t-tests, with p-values adjusted for multiple testing.

Taqman Array Cards (TAC): Pathogen burden will be assessed using TAC cards (Figure 2) as performed previously.[40 41 42] This will be performed at in the laboratory at Haydom Global Health Research Center using. Briefly, stool samples undergo nucleic acid extraction via the QIAamp Fast Stool DNA Mini kit (Valencia, CA). Nucleic acids are mixed with AgPath-ID One-Step RT-PCR buffer (Thermo Fisher) and nuclease free water to a 100ul final volume and run on a custom-designed TAC[40]:

Antibiotic resistance: We will perform stool bacterial culture on a subset of participants to monitor for development of antimicrobial resistance, which is a valid concern of any mass antimicrobial administration. Briefly, stool will be cultured on MacConkey agar and 5 lactose fermenting E. coli colonies pooled and tested for AMR using disc diffusion to screen for ESBL, AmpC, carbapenemase and susceptibility to antibiotics commonly in use locally. Azrythromycin susceptibility will be tested by E-Test.[43]

Myeloperoxidase: As an assessment of stool inflammation, myeloperoxidase will be assessed at months 6, 12 and 18. Testing will be performed at the Global Health Research Center laboratory in Haydom using a commercially-available assay (Alpco, Salem, NH).

Urine

Urine samples at 6, 12 and 18 months will assessed alongside serum and plasma samples at Imperial College, London for untargeted metabolomics analysis by NMR spectroscopy and UPLC-MS, as described above.[38]

For peer review only - http://bmjopen.bmj.com/site/about/guidelines.xhtml

Saliva

For a more complete assessment of these interventions on intestinal inflammation in this setting, we will test mothers and children for secretor status of fucosyltransferase 2 (*FUT2*), a regulator of blood group antigens on the intestinal mucosa, as non-secretor status has been associated with infections[44] and other forms of colitis.[45] DNA for mothers and children will be extracted from salivary samples at UVa and *FUT2* genotype will be determined by dot blot assays in a hierarchical method based largely on Lewis Type a and b antigens. Lewis double negatives (5-7%) are not able to be characterized as for their secretor status and so will be typed for A, B, and H antigens to allow for phenotypic characterization of all samples.[46]

Breath

To interrogate these interventions for changes in SIBO, a pathophysiological process associated with poor linear growth, we will assess hydrogen and methane quantity of breath samples in a subset of 200 participants. Breath samples collected in glass vials will be extracted and analyzed using a Quintron BreathTracker SC breath chromatograph (Quintron Inc, Milwaukee, WI USA).[33] Data on both hydrogen and methane production will be collected.

Data management and confidentiality

Data are collected onto paper forms, which are then quality checked before being double-entered by two different study team data clerks independently at the research site into a secure web-based system (Multi-Schema Information Capture, MuSIC) managed by UVa Department of Public Health Sciences. Any discrepancies in this double-data entry are reconciled by assessing the original paper form.

Data analysis

<u>Primary endpoint</u>

For peer review only - http://bmjopen.bmj.com/site/about/guidelines.xhtml

The primary outcome for the overall study is LAZ at 18 months. For each of the intervention domains we will compare this outcome between those randomized to receive the intervention vs. those who did not. This will be performed using a modified intent-to-treat analysis, assessing participants who complete the study and have a measurement of LAZ at 18 months.

<u>Secondary endpoints</u>

Each of the intervention domains has its own set of secondary outcomes as well (see Table 1), and these will be compared between those who receive the intervention and those who do not. This will initially be performed using the same modified intent-to-treat analysis assessing those with the individual outcome of interest. Secondary analysis for all endpoints (including LAZ at 18 months) will also be performed using per-protocol treatment, defined as participants meeting the following criteria:

- Breastfed through age 6 months
- Received all doses of azithromycin
- Received initial dose of nitazoxanide
- Received at least 50% of nicotinamide doses as measured by pill-counting.
- Have the outcome of interest measured.

Data and safety monitoring

A data and safety monitoring board has been formed to review data regarding safety of these interventions. This board contains four experts and physicians from Tanzania, the US and the United Kingdom with specialties in gastroenterology, global health, pediatrics, and statistics to assist with reviewing data related to adverse events, health outcomes and surveillance cultures evaluating for antibiotic resistance. Adverse event data are reported

For peer review only - http://bmjopen.bmj.com/site/about/guidelines.xhtml

monthly. The DSMB makes recommendations regarding ongoing safety of the interventions and any need to be un-blinded as to treatment allocation or to discontinue any of the treatment arms.

Statistical power

The primary outcome is difference in LAZ at 18 months. The sample size was calculated to provide sufficient power for testing the main effects for the two interventions in a 2 x 2 factorial design. If each main effect were tested at the 5% levels, as is customary in the analysis of factorial studies,[47 48] with 270 participants per group, there is 80% power for a difference in LAZ of 0.176. This is within the range of improvement of an increase of 0.16 seen in a prior study after 12 months of micronutrient supplementation,[49] though in Haydom the growth and deficiencies are more severe than most areas, thus the potential for observing a greater effect size. The interaction between main effects will then be estimated and tested as a secondary analysis. If we were to take a more conservative approach and use a significance level of 2.5% for each of the main effects, with 270 subjects per group, we would still have 80% power to detect an average LAZ increase of 0.193. Finally, if we were to adjust the significance to include the test for the interaction, the F-test for the main effect in a 2-way ANOVA has 80% power with a two-sided significance level of 1.67%, when the main effect of the intervention is to increase the mean LAZ by 0.203. All of these are reasonable changes in LAZ to observe for in the Haydom population, given the considerable demonstrated mean deficit in growth. Adjusting for 10% dropout, the total sample size required is 1188. Within our recruitment radius >2507 births occur yearly. We will recruit during a 12-month window. In our extensive research experience at the Haydom MAL-ED site, we observed 0% refusal to participate and a high 81% retention rate by 4 years of follow-up during MAL-ED (and, thus, a study of shorter duration may have improved follow-up). Based on this, we anticipate a high rate of recruitment (>90%) and 18-month retention (>90%). Therefore, accounting for drop-out, we anticipate achieving 1188

For peer review only - http://bmjopen.bmj.com/site/about/guidelines.xhtml

mother/child dyads divided between intervention and control, leaving us adequate power to determine differences in LAZ. In addition to standard 2-way ANOVA to assess main effects and the interaction, linear and logistic regression will be used to evaluate the effect of the individual intervention domains on outcomes after taking into account individual maternal and child characteristics such as socioeconomic status, maternal height and enrollment weight as covariates.

Study timeline

Enrollment began 5 September 2017 and is planned to continue through 5 September 2018. Children will be followed until age 18 months, resulting in a planned completion in February 2020. Blinding will be maintained until all final study visits are completed. The primary analysis will be performed once data collection is complete for the variables involved. To reduce intra-assay variability, the majority of laboratory testing will be batched and performed once complete samples are present.

Potential challenges and limitations

Potential challenges include adequate recruitment of target participant numbers within the one-year study window. Should this be true, we will consider extending the recruitment window. Maintaining adherence of study medication, particularly the daily doses of nicotinamide to breast-feeding mothers during months 0-6, and infants during months 6-18, may be a challenge in a setting in which few individuals are accustomed to taking daily medication or nutritional supplements. To protect against adherence challenges, we performed a pilot study beforehand, identifying potential barriers to adherence and resulting in steps to remind mothers frequently during the first month of the study, with follow-up home visits by the field team and by local community healthcare workers.

For peer review only - http://bmjopen.bmj.com/site/about/guidelines.xhtml

ETHICS AND DISSEMINATION

Regulatory authorities

ELICIT has received approval from Tanzania's National Institute of Medical Research (NIMR, reference number HQ/R.8a/Vol.IX/2424, approved 3 March 2017), the University of Virginia Health Sciences Research Institutional Review Board (HSR-IRB, reference #19465, approved 19 January 2017), and the Tanzanian Food and Drug Administration (TFDA, reference number TFDA0017/CTR/0005/02, approved 5 July 2017). All research procedures are conducted in accordance with the Declaration of Helsinki and according to International Conference on Harmonisation Good Clinical Practice guidelines. All participant mothers provide informed consent. An external monitor (FHI360) evaluated the site prior to initiation of the study and performs yearly site visits and assessments thereafter.

Dissemination

This trial has been registered at ClinicalTrials.gov (#NCT03268902). Scientific reports resulting from this study will be made publicly available. Following completion of data accrual, data will be made publicly available in an online database. Data will be presented at major conferences such as those of the American Society of Tropical Medicine and Hygiene, the Endocrine Society, and the African Academy of Sciences. Scientific articles will be published with open-access availability at major scientific journals.

Authors' contributions

MDD, JPM, RJS, JM, JG, ES, EH and EM conceived of this trial and developed this study protocol. EM is the Principal Investigator; MDD is the UVa Principal Investigator. SJ is the

For peer review only - http://bmjopen.bmj.com/site/about/guidelines.xhtml

Clinical Research Coordinator; AW is the UVa Project Manager. JPM, JG and EH developed procedures related to stool testing for pathogens and antibiotic resistance. JS developed procedures related to metabolomics testing. JRD developed procedures related to testing for small intestinal bacterial overgrowth. JMM developed procedures related to the breastfeeding support of mothers for community, field and nursing staff. RJS and ES developed procedures related to developmental testing. JG oversees all sample processing and laboratory procedures in Haydom. All authors contributed to the development of this manuscript and/or study procedures and to reading and approving the final version for publication.

Funding statement

This work was supported by the Bill and Melinda Gates Foundation, OPP1141342.

Role of the sponsor and funder

The funder contributed input into the design of the study. Neither the sponsor nor the funder had input into the preparation of this report for publication. The sponsor has ultimate authority into these activities.

Acknowledgments

Romark donated the nitazoxanide.

Competing interests statement

None of the authors or study co-investigators have any competing interests to declare.

REFERENCES

1. Grantham-McGregor S, Cheung YB, Cueto S, et al. Child development in developing countries 1 - Developmental potential in the first 5 years for children in developing countries. *Lancet* 2007;369(9555):60-70.

For peer review only - http://bmjopen.bmj.com/site/about/guidelines.xhtml

2. Prendergast AJ, Humphrey JH. The stunting syndrome in developing countries. *Paediatrics and International Child Health* 2014;34(4):250-65. doi: 10.1179/2046905514y.0000000158
3. Kosek MN, Investigators M-EN. Causal Pathways from Enteropathogens to Environmental Enteropathy: Findings from the MAL-ED Birth Cohort Study. *EBioMedicine* 2017;18:109-17. doi: 10.1016/j.ebiom.2017.02.024 [published Online First: 2017/03/08]
4. Mduma ER, Gratz J, Patil C, et al. The etiology, risk factors, and interactions of enteric infections and malnutrition and the consequences for child health and development study (MAL-ED): description of the Tanzanian site. *Clin Infect Dis* 2014;59 Suppl 4:S325-30. doi: 10.1093/cid/ciu439
5. Investigators M-EN. Childhood stunting in relation to the pre- and postnatal environment during the first 2 years of life: The MAL-ED longitudinal birth cohort study. *PLoS Med* 2017;14(10):e1002408. doi: 10.1371/journal.pmed.1002408 [published Online First: 2017/10/25]
6. Scharf RJ, Rogawski ET, Murray-Kolb LE, et al. Early Childhood Growth and Cognitive Outcomes: Findings from the MAL-*ED Study*. *Maternal and Child Nutrition* 2017;in press
7. Krebs NF, Lozoff B, Georgieff MK. Neurodevelopment: The Impact of Nutrition and Inflammation During Infancy in Low-Resource Settings. *Pediatrics* 2017;139(Suppl 1):S50-S58. doi: 10.1542/peds.2016-2828G
8. Platts-Mills JA, Babji S, Bodhidatta L, et al. Pathogen-specific burdens of community diarrhoea in developing countries: a multisite birth cohort study (MAL-ED). *Lancet Glob Health* 2015;3(9):e564-75. doi: 10.1016/S2214-109X(15)00151-5 [published Online First: 2015/07/19]
9. MAL-ED_Network_Investigators. Relationship between growth and illness, enteropathogens and dietary intakes in the first 2 years of life: findings from the MAL-ED birth cohort study. *BMJ Global Health* 2017;2(4):e000370.
10. Rogawski ET, Bartelt LA, Platts-Mills JA, et al. Determinants and Impact of Giardia Infection in the First 2 Years of Life in the MAL-ED Birth Cohort. *J Pediatric Infect Dis Soc* 2017;6(2):153-60. doi: 10.1093/jpids/piw082
11. Gough EK, Moodie EEM, Prendergast AJ, et al. The impact of antibiotics on growth in children in low and middle income countries: systematic review and meta-analysis of randomised controlled trials. *Bmj-British Medical Journal* 2014;348 doi: 10.1136/bmj.g2267
12. Porco TC, Gebre T, Ayele B, et al. Effect of Mass Distribution of Azithromycin for Trachoma Control on Overall Mortality in Ethiopian Children A Randomized Trial. *Jama-Journal of the American Medical Association* 2009;302(9):962-68.
13. Amza A, Kadri B, Nassirou B, et al. A cluster-randomized controlled trial evaluating the effects of mass azithromycin treatment on growth and nutrition in Niger. *Am J Trop Med Hyg* 2013;88(1):138-43. doi: 10.4269/ajtmh.2012.12-0284
14. Amadi B, Mwiya M, Musuku J, et al. Effect of nitazoxanide on morbidity and mortality in Zambian children with cryptosporidiosis: a randomised controlled trial. *Lancet* 2002;360(9343):1375-80. doi: 10.1016/S0140-6736(02)11401-2
15. Shi H, Enriquez A, Rapadas M, et al. NAD Deficiency, Congenital Malformations, and Niacin Supplementation. *N Engl J Med* 2017;377(6):544-52. doi: 10.1056/NEJMoa1616361
16. Chen C, Mitchell NJ, Gratz J, et al. Fumonisin, Not Aflatoxin, is Associated with Growth Impairment in Tanzanian

Children. *Environmental International* 2018;in press
17. Le Floc'h N, Otten W, Merlot E. Tryptophan metabolism, from nutrition to potential therapeutic applications. *Amino Acids* 2011;41(5):1195-205. doi: 10.1007/s00726-010-0752-7

For peer review only - http://bmjopen.bmj.com/site/about/guidelines.xhtml

18. Perlot T, Penninger JM. ACE2 - from the renin-angiotensin system to gut microbiota and malnutrition. *Microbes Infect* 2013;15(13):866-73. doi: 10.1016/j.micinf.2013.08.003
19. Hashimoto T, Perlot T, Rehman A, et al. ACE2 links amino acid malnutrition to microbial ecology and intestinal inflammation. *Nature* 2012;487(7408):477-81. doi: 10.1038/nature11228 [published Online First: 2012/07/25]
20. Wolf AM, Wolf D, Rumpold H, et al. Overexpression of indoleamine 2,3-dioxygenase in human inflammatory bowel disease. *Clin Immunol* 2004;113(1):47-55. doi: 10.1016/j.clim.2004.05.004
21. Gupta NK, Thaker AI, Kanuri N, et al. Serum analysis of tryptophan catabolism pathway: correlation with Crohn's disease activity. *Inflamm Bowel Dis* 2012;18(7):1214-20. doi: 10.1002/ibd.21849 [published Online First: 2011/08/05]
22. Favre D, Mold J, Hunt PW, et al. Tryptophan catabolism by indoleamine 2,3-dioxygenase 1 alters the balance of TH17 to regulatory T cells in HIV disease. *Sci Transl Med* 2010;2(32):32ra36. doi: 10.1126/scitranslmed.3000632
23. Mayneris-Perxachs J, Lima AA, Guerrant RL, et al. Urinary N-methylnicotinamide and β-aminoisobutyric acid predict catch-up growth in undernourished Brazilian children. *Sci Rep* 2016;6:19780. doi: 10.1038/srep19780
24. Kosek MN, Mduma E, Kosek PS, et al. Plasma Tryptophan and the Kynurenine-Tryptophan Ratio are Associated with the Acquisition of Statural Growth Deficits and Oral Vaccine Underperformance in Populations with Environmental Enteropathy. *Am J Trop Med Hyg* 2016;95(4):928-37. doi: 10.4269/ajtmh.16-0037 [published Online First: 2016/08/08]
25. Allen LH, Hampel D, Shahab-Ferdows S, et al. Antiretroviral therapy provided to HIV-infected Malawian women in a randomized trial diminishes the positive effects of lipid-based nutrient supplements on breast-milk B vitamins. *Am J Clin Nutr* 2015;102(6):1468-74. doi: 10.3945/ajcn.114.105106
26. Obradović J, Yousafzai AK, Finch JE, et al. Maternal scaffolding and home stimulation: Key mediators of early intervention effects on children's cognitive development. *Dev Psychol* 2016;52(9):1409-21. doi: 10.1037/dev0000182 [published Online First: 2016/08/08]
27. Walker SP, Wachs TD, Grantham-McGregor S, et al. Child Development 1 Inequality in early childhood: risk and protective factors for early child development. *Lancet* 2011;378(9799):1325-38. doi: 10.1016/s0140-6736(11)60555-2
28. Rasheed MA, Yousafzai AK. The development and reliability of an observational tool for assessing mother-child interactions in field studies- experience from Pakistan. *Child Care Health Dev* 2015 doi: 10.1111/cch.12287
29. Gladstone M, Lancaster GA, Umar E, et al. The Malawi Developmental Assessment Tool (MDAT): the creation, validation, and reliability of a tool to assess child development in rural African settings. *PLoS Med* 2010;7(5):e1000273. doi: 10.1371/journal.pmed.1000273
30. Bayley N. Bayley Scales of Infant and Toddler Development. San Antonio, TX: The Psychological Corporation 2006.
31. Murray-Kolb LE, Rasmussen ZA, Scharf RJ, et al. The MAL-ED Cohort Study: Methods and Lessons Learned When Assessing Early Child Development and Caregiving Mediators in Infants and Young Children in 8 Low- and Middle-Income Countries. *Clinical Infectious Diseases* 2014;59:S261-S72. doi: 10.1093/cid/ciu437
32. Donowitz JR, Petri WA. Pediatric small intestine bacterial overgrowth in low-income countries. *Trends Mol Med* 2015;21(1):6-15. doi: 10.1016/j.molmed.2014.11.001 [published Online First: 2014/11/15]
33. Donowitz JR, Haque R, Kirkpatrick BD, et al. Small Intestine Bacterial Overgrowth and Environmental Enteropathy in Bangladeshi Children. *MBio* 2016;7(1):e02102-15. doi: 10.1128/mBio.02102-15 [published Online First: 2016/01/12]

For peer review only - http://bmjopen.bmj.com/site/about/guidelines.xhtml

34. Trammell SA, Schmidt MS, Weidemann BJ, et al. Nicotinamide riboside is uniquely and orally bioavailable in mice and humans. *Nat Commun* 2016;7:12948. doi: 10.1038/ncomms12948 [published Online First: 2016/10/10]
35. Buchanan AM, Fiorillo SP, Omondi MW, et al. Establishment of biochemistry reference values for healthy Tanzanian infants, children and adolescents in Kilimanjaro Region. *Trop Med Int Health* 2015;20(11):1569-77. doi: 10.1111/tmi.12580 [published Online First: 2015/08/25]
36. Coghlan RF, Oberdorf JA, Sienko S, et al. A degradation fragment of type X collagen is a real-time marker for bone growth velocity. *Science Translational Medicine* 2017
37. Inagaki T, Moschetta A, Lee YK, et al. Regulation of antibacterial defense in the small intestine by the nuclear bile acid receptor. *Proc Natl Acad Sci U S A* 2006;103(10):3920-5. doi: 10.1073/pnas.0509592103 [published Online First: 2006/02/10]
38. Claus SP, Swann JR. Nutrimetabonomics: Applications for Nutritional Sciences, with Specific Reference to Gut Microbial Interactions. In: Doyle MP, Klaenhammer TR, eds. Annual Review of Food Science and Technology, Vol 4. Palo Alto: Annual Reviews 2013:381-99.
39. Ghodsi M, Liu B, Pop M. DNACLUST: accurate and efficient clustering of phylogenetic marker genes. *BMC Bioinformatics* 2011;12:271. doi: 10.1186/1471-2105-12-271
40. Liu J, Gratz J, Amour C, et al. A laboratory-developed TaqMan Array Card for simultaneous detection of 19 enteropathogens. *J Clin Microbiol* 2013;51(2):472-80. doi: 10.1128/JCM.02658-12 [published Online First: 2012/11/21]
41. Liu J, Gratz J, Amour C, et al. Optimization of Quantitative PCR Methods for Enteropathogen Detection. *PLoS One* 2016;11(6):e0158199. doi: 10.1371/journal.pone.0158199 [published Online First: 2016/06/23]
42. Liu J, Platts-Mills JA, Juma J, et al. Use of quantitative molecular diagnostic methods to identify causes of diarrhoea in children: a reanalysis of the GEMS case-control study. *Lancet* 2016;388(10051):1291-301. doi: 10.1016/S0140-6736(16)31529-X
43. Clinical_and_Laboratory_Standards_Institute. Performance Standards for Antimicrobial Susceptibility Testing, 27 ed. Wayne, Pennsylvania: Clinical and Laboratory Standards Institute 2017.
44. Ikehara Y, Nishihara S, Yasutomi H, et al. Polymorphisms of two fucosyltransferase genes (Lewis and Secretor genes) involving type I Lewis antigens are associated with the presence of anti-Helicobacter pylori IgG antibody. *Cancer Epidemiol Biomarkers Prev* 2001;10(9):971-7.
45. McGovern DP, Jones MR, Taylor KD, et al. Fucosyltransferase 2 (FUT2) non-secretor status is associated with Crohn's disease. *Hum Mol Genet* 2010;19(17):3468-76. doi: 10.1093/hmg/ddq248
46. Marionneau S, Airaud F, Bovin NV, et al. Influence of the combined ABO, FUT2, and FUT3 polymorphism on susceptibility to Norwalk virus attachment. *J Infect Dis* 2005;192(6):1071-7. doi: 10.1086/432546 [published Online First: 2005/08/09]
47. Yusuf S, Lonn E, Pais P, et al. Blood-Pressure and Cholesterol Lowering in Persons without Cardiovascular Disease. *N Engl J Med* 2016;374(21):2032-43. doi: 10.1056/NEJMoa1600177
48. Locks LM, Manji KP, McDonald CM, et al. Effect of zinc and multivitamin supplementation on the growth of Tanzanian children aged 6-84 wk: a randomized, placebo-controlled, double-blind trial. *Am J Clin Nutr* 2016;103(3):910-8. doi: 10.3945/ajcn.115.120055
49. Soofi S, Cousens S, Iqbal SP, et al. Effect of provision of daily zinc and iron with several micronutrients on growth and morbidity among young children in Pakistan: a cluster-randomised trial. *Lancet* 2013;382(9886):29-40. doi: 10.1016/S0140-6736(13)60437-7

For peer review only - http://bmjopen.bmj.com/site/about/guidelines.xhtml

FIGURE HEADINGS

Figure 1: The Tryptophan-kynurenine-niacin pathway in the intestinal mucosa. Dietary tryptophan is metabolized to kynurenine by indoleamine 2,3-dioxygenase (IDO)—whose activity is increased in settings of inflammation. Adverse effects of elevated IDO activity relate to modulation of the immune response, decreased activity of mTOR in producing antimicrobial peptides, and decreased tryptophan for protein synthesis. Intervention with nicotinamide ameliorates some of these effects in preclinical models; in the current study, we hypothesize that intervention with nicotinamide will increase linear growth via normalization of the tryptophan-kynurenine-niacin pathway and restored enterocyte activity. Both nicotinic acid and nicotinamide are considered dietary forms of niacin. Abbreviations: IDO = indoleamine 2,3-dioxygenase; NAD = nicotinamide adenine dinucleotide.

Figure 2: Taqman Array Card (TAC) schematic. Burden of intestinal pathogens will be assessed in stool samples from participants at multiple time points using TAC.

For peer review only

For peer review only - http://bmjopen.bmj.com/site/about/guidelines.xhtml

Table 1: Primary and secondary outcome measures by study intervention.

Outcome	Timing (age of child in months)	Antimicrobial intervention		Nicotinamide intervention
Primary outcome				
LAZ	18	X		X
Secondary outcomes				
• Proportion of stunting (LAZ<-2)	18	X		X
• Weight-for-age (WAZ) and weight-for-length (WLZ) z-scores	18	X		X
• Metabolomic assay of gut microbial metabolism and alterations to tryptophan-niacin and other pathways	6, 12, 18	X		X
• Tryptophan:kynurenine ratio and other pathway metabolites in serum	12, 18	X		X
• IGF-1 at 12 and 18 months	12, 18	X		X
• High-sensitivity C-reactive protein at 12 and 18 months	12, 18	X		X
• Stool myeloperoxidase at months 6,12 and 18 months	6, 12, 18	X		X
• Prevalence of enteric pathogens	6, 6.5, 12, 12.5,18	X		X
• Change in microbiota (by traditional sequencing) (subset of participants)	6, 6.5, 12, 18	X		X
• Change in small intestinal bacterial overgrowth as assessed by exhaled increased hydrogen following ingestion of sugar (subset of participants)	6, 12, 18	X		X
• Difference in breast milk composition (nicotinamide, tryptophan, amino acids)	1, 5			X
• Symptomatic diarrhea		X		X
• Hospitalization and all-cause mortality	18	X		X
• Cognitive outcomes Malawi Development Assessment Tool (MDAT) assessment and the Bayley Scales of Infant and Toddler Development	18	X		X

For peer review only

For peer review only - http://bmjopen.bmj.com/site/about/guidelines.xhtml

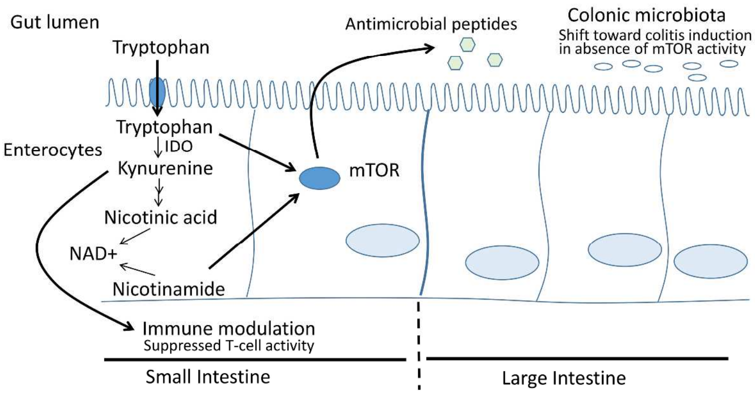

Figure 1: The Tryptophan-kynurenine-niacin pathway in the intestinal mucosa. Dietary tryptophan is metabolized to kynurenine by indoleamine 2,3-dioxygenase (IDO)—whose activity is increased in settings of inflammation. Adverse effects of elevated IDO activity relate to modulation of the immune response, decreased activity of mTOR in producing antimicrobial peptides, and decreased tryptophan for protein synthesis. Intervention with nicotinamide ameliorates some of these effects in preclinical models; in the current study, we hypothesize that intervention with nicotinamide will increase linear growth via normalization of the tryptophan-kynurenine-niacin pathway and restored enterocyte activity. Both nicotinic acid and nicotinamide are considered dietary forms of niacin. Abbreviations: IDO = indoleamine 2,3-dioxygenase; NAD = nicotinamide adenine dinucleotide.

338x190mm (300 x 300 DPI)

For peer review only - http://bmjopen.bmj.com/site/about/guidelines.xhtml

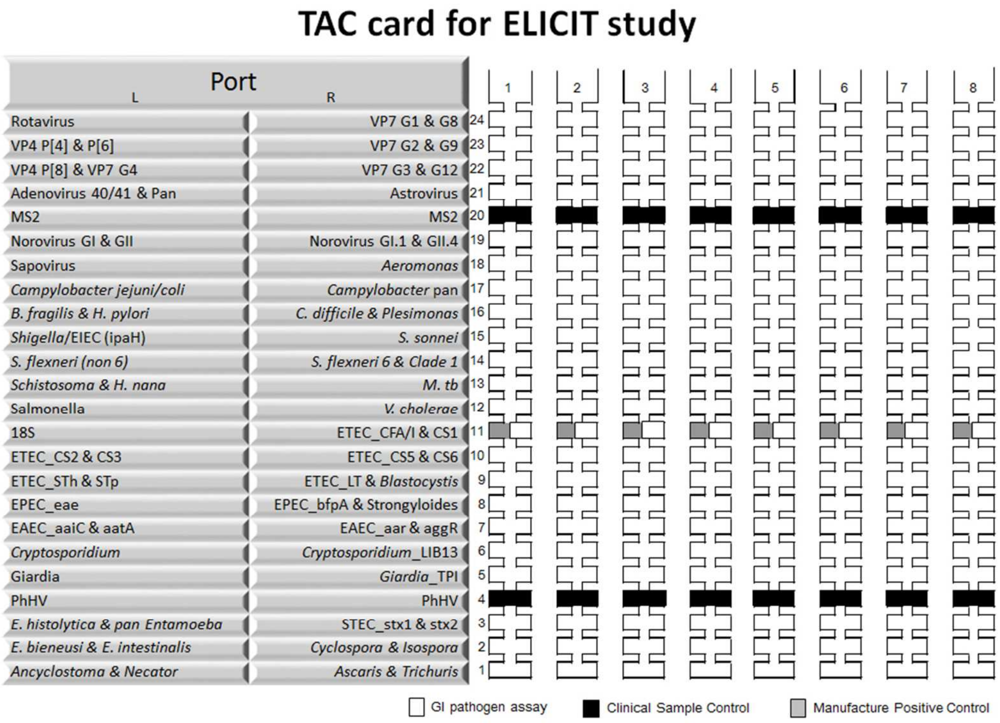

336x248mm (72 x 72 DPI)

SPIRIT

STANDARD PROTOCOL ITEMS: RECOMMENDATIONS FOR INTERVENTIONAL TRIALS

SPIRIT 2013 Checklist: Recommended items to address in a clinical trial protocol and related documents*

Section/item	Item No	Description	Addressed on page number
**Administrative information**			
Title	1	Descriptive title identifying the study design, population, interventions, and, if applicable, trial acronym	1
Trial registration	2a	Trial identifier and registry name. If not yet registered, name of intended registry	2
	2b	All items from the World Health Organization Trial Registration Data Set	
Protocol version	3	Date and version identifier	3
Funding	4	Sources and types of financial, material, and other support	23
Roles and responsibilities	5a	Names, affiliations, and roles of protocol contributors	22-23
	5b	Name and contact information for the trial sponsor	3
	5c	Role of study sponsor and funders, if any, in study design; collection, management, analysis, and interpretation of data; writing of the report; and the decision to submit the report for publication, including whether they will have ultimate authority over any of these activities	23
	5d	Composition, roles, and responsibilities of the coordinating centre, steering committee, endpoint adjudication committee, data management team, and other individuals or groups overseeing the trial, if applicable (see Item 21a for data monitoring committee)	NA

For peer review only

**Introduction**			
Background and rationale	6a	Description of research question and justification for undertaking the trial, including summary of relevant studies (published and unpublished) examining benefits and harms for each intervention	______5-7_____
	6b	Explanation for choice of comparators	______7_______
Objectives	7	Specific objectives or hypotheses	______7-8_____
Trial design	8	Description of trial design including type of trial (eg, parallel group, crossover, factorial, single group), allocation ratio, and framework (eg, superiority, equivalence, noninferiority, exploratory)	______7-8_____
**Methods: Participants, interventions, and outcomes**			
Study setting	9	Description of study settings (eg, community clinic, academic hospital) and list of countries where data will be collected. Reference to where list of study sites can be obtained	______9_______
Eligibility criteria	10	Inclusion and exclusion criteria for participants. If applicable, eligibility criteria for study centres and individuals who will perform the interventions (eg, surgeons, psychotherapists)	______8_______
Interventions	11a	Interventions for each group with sufficient detail to allow replication, including how and when they will be administered	______9-10____
	11b	Criteria for discontinuing or modifying allocated interventions for a given trial participant (eg, drug dose change in response to harms, participant request, or improving/worsening disease)	_____19-20____
	11c	Strategies to improve adherence to intervention protocols, and any procedures for monitoring adherence (eg, drug tablet return, laboratory tests)	______10______
	11d	Relevant concomitant care and interventions that are permitted or prohibited during the trial	______10_______
Outcomes	12	Primary, secondary, and other outcomes, including the specific measurement variable (eg, systolic blood pressure), analysis metric (eg, change from baseline, final value, time to event), method of aggregation (eg, median, proportion), and time point for each outcome. Explanation of the clinical relevance of chosen efficacy and harm outcomes is strongly recommended	_18-19, Table 1__
Participant timeline	13	Time schedule of enrolment, interventions (including any run-ins and washouts), assessments, and visits for participants. A schematic diagram is highly recommended (see Figure)	_____9-10______

For peer review only - http://bmjopen.bmj.com/site/about/guidelines.xhtml

Sample size	14	Estimated number of participants needed to achieve study objectives and how it was determined, including clinical and statistical assumptions supporting any sample size calculations	______20_______
Recruitment	15	Strategies for achieving adequate participant enrolment to reach target sample size	______21_______
**Methods: Assignment of interventions (for controlled trials)**			
Allocation:			
Sequence generation	16a	Method of generating the allocation sequence (eg, computer-generated random numbers), and list of any factors for stratification. To reduce predictability of a random sequence, details of any planned restriction (eg, blocking) should be provided in a separate document that is unavailable to those who enrol participants or assign interventions	______9_______
Allocation concealment mechanism	16b	Mechanism of implementing the allocation sequence (eg, central telephone; sequentially numbered, opaque, sealed envelopes), describing any steps to conceal the sequence until interventions are assigned	______9_______
Implementation	16c	Who will generate the allocation sequence, who will enrol participants, and who will assign participants to interventions	______9_______
Blinding (masking)	17a	Who will be blinded after assignment to interventions (eg, trial participants, care providers, outcome assessors, data analysts), and how	______9-10_____
	17b	If blinded, circumstances under which unblinding is permissible, and procedure for revealing a participant's allocated intervention during the trial	______19-20____
**Methods: Data collection, management, and analysis**			
Data collection methods	18a	Plans for assessment and collection of outcome, baseline, and other trial data, including any related processes to promote data quality (eg, duplicate measurements, training of assessors) and a description of study instruments (eg, questionnaires, laboratory tests) along with their reliability and validity, if known. Reference to where data collection forms can be found, if not in the protocol	_____10-18______
	18b	Plans to promote participant retention and complete follow-up, including list of any outcome data to be collected for participants who discontinue or deviate from intervention protocols	_____9-10______

For peer review only - http://bmjopen.bmj.com/site/about/guidelines.xhtml

Data management	19	Plans for data entry, coding, security, and storage, including any related processes to promote data quality (eg, double data entry; range checks for data values). Reference to where details of data management procedures can be found, if not in the protocol	_____18________
Statistical methods	20a	Statistical methods for analysing primary and secondary outcomes. Reference to where other details of the statistical analysis plan can be found, if not in the protocol	_____18-19_____
	20b	Methods for any additional analyses (eg, subgroup and adjusted analyses)	_____19_______
	20c	Definition of analysis population relating to protocol non-adherence (eg, as randomised analysis), and any statistical methods to handle missing data (eg, multiple imputation)	_____19_______
**Methods: Monitoring**			
Data monitoring	21a	Composition of data monitoring committee (DMC); summary of its role and reporting structure; statement of whether it is independent from the sponsor and competing interests; and reference to where further details about its charter can be found, if not in the protocol. Alternatively, an explanation of why a DMC is not needed	_____19-20_____
	21b	Description of any interim analyses and stopping guidelines, including who will have access to these interim results and make the final decision to terminate the trial	_____19-20_____
Harms	22	Plans for collecting, assessing, reporting, and managing solicited and spontaneously reported adverse events and other unintended effects of trial interventions or trial conduct	_____19-20_____
Auditing	23	Frequency and procedures for auditing trial conduct, if any, and whether the process will be independent from investigators and the sponsor	______18_______
**Ethics and dissemination**			
Research ethics approval	24	Plans for seeking research ethics committee/institutional review board (REC/IRB) approval	______22_______
Protocol amendments	25	Plans for communicating important protocol modifications (eg, changes to eligibility criteria, outcomes, analyses) to relevant parties (eg, investigators, REC/IRBs, trial participants, trial registries, journals, regulators)	______22_______

For peer review only - http://bmjopen.bmj.com/site/about/guidelines.xhtml

Consent or assent	26a	Who will obtain informed consent or assent from potential trial participants or authorised surrogates, and how (see Item 32)	_____9________
	26b	Additional consent provisions for collection and use of participant data and biological specimens in ancillary studies, if applicable	_____9________
Confidentiality	27	How personal information about potential and enrolled participants will be collected, shared, and maintained in order to protect confidentiality before, during, and after the trial	_____18________
Declaration of interests	28	Financial and other competing interests for principal investigators for the overall trial and each study site	_____23________
Access to data	29	Statement of who will have access to the final trial dataset, and disclosure of contractual agreements that limit such access for investigators	_____18________
Ancillary and post-trial care	30	Provisions, if any, for ancillary and post-trial care, and for compensation to those who suffer harm from trial participation	_____19-20_____
Dissemination policy	31a	Plans for investigators and sponsor to communicate trial results to participants, healthcare professionals, the public, and other relevant groups (eg, via publication, reporting in results databases, or other data sharing arrangements), including any publication restrictions	________22______
	31b	Authorship eligibility guidelines and any intended use of professional writers	________22______
	31c	Plans, if any, for granting public access to the full protocol, participant-level dataset, and statistical code	________22______
**Appendices**			
Informed consent materials	32	Model consent form and other related documentation given to participants and authorised surrogates	__Supplement__
Biological specimens	33	Plans for collection, laboratory evaluation, and storage of biological specimens for genetic or molecular analysis in the current trial and for future use in ancillary studies, if applicable	____10-18______

*It is strongly recommended that this checklist be read in conjunction with the SPIRIT 2013 Explanation & Elaboration for important clarification on the items. Amendments to the protocol should be tracked and dated. The SPIRIT checklist is copyrighted by the SPIRIT Group under the Creative Commons "Attribution-NonCommercial-NoDerivs 3.0 Unported" license.

For peer review only - http://bmjopen.bmj.com/site/about/guidelines.xhtml

BMJ Open

Early Life Interventions for Childhood Growth and Development in Tanzania (ELICIT): A Protocol for a Randomized Factorial, Double-Blind, Placebo-Controlled Trial of Azithromycin, Nitazoxanide and Nicotinamide

Journal:	*BMJ Open*
Manuscript ID	bmjopen-2018-021817.R1
Article Type:	Protocol
Date Submitted by the Author:	16-Mar-2018

Complete List of Authors:	DeBoer, Mark; University of Virginia, Pediatrics<br>Platts-Mills, James; University of Virginia, Medicine<br>Scharf, Rebecca; University of Virginia Children's Hospital, Pediatrics<br>McDermid, Joann; University of Virginia, Medicine<br>Wanjuhi, Anne; University of Virginia, Global Health<br>Gratz, Jean; University of Virginia, Medicine<br>Svensen, Erling; Haukeland Universitetssjukehus<br>Swann, Jon; Imperial College London<br>Donowitz, Jeffrey; University of Virginia, Medicine<br>Jatosh, Samwel; Haydom Lutheran Hospital, Haydom Global Health Research Center<br>Houpt, Eric; University of Virginia,<br>Mduma, Estomih ; Haydom Lutheran Hospital

**Primary Subject Heading**:	Global health
Secondary Subject Heading:	Infectious diseases, Nutrition and metabolism, Paediatrics, Public health
Keywords:	growth, stunting, development, antibiotics, niacin, Tanzania

SCHOLARONE™
Manuscripts

Early Life Interventions for Childhood Growth and Development in Tanzania (ELICIT): A Protocol for a Randomized Factorial, Double-Blind, Placebo-Controlled Trial of Azithromycin, Nitazoxanide and Nicotinamide

Mark D. DeBoer MD, MSc, MCR[1,7]; James A. Platts-Mills MD[2]; Rebecca J. Scharf MD, MPH[1,2]; Joann M. McDermid Ph.D, RD[2]; Anne Wanjuhi MPH[1]; Jean Gratz MS[2]; Erling Svensen Ph.D[3]; Jon R. Swann Ph.D[4]; Jeffrey R. Donowitz MD[5]; Samwel Jatosh BS[6]; Eric R. Houpt MD[2]; Estomih Mduma MPH[6]

1. Department of Pediatrics, University of Virginia, Charlottesville, VA, USA;
2. Center for Global Health, Department of Medicine, University of Virginia, Charlottesville, VA, USA;
3. Department of Global Health and Primary Care, University of Bergen, Bergen, Norway;
4. Imperial College of London, London, UK;
5. Division of Infectious Disease, Children's Hospital of Richmond at Virginia Commonwealth University
6. Haydom Global Health Research Centre, Haydom, Tanzania;
7. Corresponding Author:

Division of Pediatric Endocrinology
University of Virginia School of Medicine
P.O. Box 800386
Charlottesville, VA 22908
Phone: 434-924-5956
Fax: 434-924-9181
Email: deboer@virginia.edu

For peer review only
For peer review only - http://bmjopen.bmj.com/site/about/guidelines.xhtml

ABSTRACT

Introduction: In many developing areas in the world, a high burden of enteric pathogens in early childhood are associated with growth deficits. The tryptophan-kynurenine-niacin pathway has been linked to enteric inflammatory responses to intestinal infections. However, it is not known in these settings whether scheduled antimicrobial intervention to reduce subclinical enteric pathogen carriage or repletion of the tryptophan-kynurenine-niacin pathway improves linear growth and development.

Methods and analysis: We are conducting a randomized, placebo-controlled, factorial intervention trial in the rural setting of Haydom, Tanzania. We are recruiting 1188 children within the first 14 days of life, who will be randomized in a 2x2 factorial design to administration of antimicrobials (azithromycin and nitazoxanide, randomized together) and nicotinamide. The nicotinamide is administered as a daily oral dose, which for breast-feeding children aged 0-6 months is given to the mother and for children aged 6-18 months is given to the child directly. Azithromycin is given to the child as a single oral dose at months 6, 9, 12, and 15; nitazoxanide is given as a 3-day course at months 12 and 15. Mother/child pairs are followed via monthly in-home visits. The primary outcome is the child's length-for-age Z-score at 18 months. Secondary outcomes for the child include additional anthropometry measures; stool pathogen burden and bacterial microbiome; systemic and enteric inflammation; blood metabolomics, growth factors, inflammation and nutrition; hydrogen breath assessment to estimate small-intestinal bacterial overgrowth; and assessment of cognitive development. Secondary outcomes for the mother include breastmilk content of nicotinamide, other vitamins and amino acids; blood measures of tryptophan-kynurenine-niacin pathway; and stool pathogens.

Ethics and dissemination: This trial has been approved by the Tanzanian National Institute for Medical Research, the Tanzanian FDA and the University of Virginia IRB. Findings will be presented at national and international conferences and published in peer-review journals.

Trial registration number: ClinicalTrials.gov #NCT03268902.

For peer review only - http://bmjopen.bmj.com/site/about/guidelines.xhtml

Key words: growth, stunting, development, antibiotics, niacin, Tanzania

Protocol version: 5.0, 4 December 2017

Protocol Sponsor: Haydom Lutheran Hospital, Haydom, Manyara, Tanzania

For peer review only

For peer review only - http://bmjopen.bmj.com/site/about/guidelines.xhtml

STRENGTHS AND LIMITATIONS OF THIS STUDY

- This study uses a factorial design to determine independent contributions of two different intervention approaches onearly childhood growth and development.
- The study is set in a rural site in sub-Saharan Africa with a high degree of both enteric pathogen carriage and stunting.
- This study assesses intervention with nicotinamide, targeting the tryptophan-kynurenine-niacin pathway, which may have important roles in intestinal bacterial content and immune and metabolic response.
- In addition to the primary outcome of length-for-age Z-score, this study measures extensive secondary outcomes related to infection, nutrition, metabolism and cognitive development.
- Due to sample size constraints, one limitation of the study is that two different antimicrobial interventions (azithromycin and nitazoxanide) are randomized together, limiting the ability to determine any individual contribution of either of these to study outcomes.

For peer review only

INTRODUCTION

Childhood growth and thriving remain sub-optimal in low-resource settings worldwide, underscoring the need to better understand underlying etiologies and to identify interventions toward improving outcomes.[1 2] The Etiology, Risk Factors, and Interactions of Enteric Infections and Malnutrition and the Consequences for Child Health (MAL-ED) study evaluated correlates of childhood growth and cognitive development in 8 low- to middle-income countries (LMIC) worldwide, including the region around Haydom, Tanzania.[3 4] Haydom is in a rural, semi-arid area with a high degree of poverty. Children in Haydom followed in MAL-ED had a prevalence of stunting of 70% at 18 months of age.[5] Data from MAL-ED also showed that linear growth deficits correlated with lower scores for cognitive development,[6] potentially through shared barriers to optimal growth and brain development.[1 7]

Antimicrobials to improve childhood growth

In MAL-ED, children in the Haydom site had a high burden of infection with intestinal pathogens starting early in life. High carriage of enteropathogens, even in the absence of diarrhea, was associated with a 1.36 increased odds of having lower length for age.[3] As for the specific enteropathogens, among the most prevalent pathogens at age 6 months were enteroaggregative *E.coli* (48%) and *Campylobacter* (27%),[8] each of which were associated with a 0.85 cm and 0.83 cm, respectively, shortfall in height attainment.[9] Both of these infections are treatable with azithromycin. Other highly prevalent pathogens by age 6 months in the MAL-ED study included *Giardia* (22%) and *Cryptosporidium* (6%), and early *Giardia* infection was also associated with growth shortfalls.[10] Both of these protozoal infections are treatable with nitazoxanide. These observations raise questions as to whether such antibiotics may be useful in ameliorating growth deficits.

A meta-analysis of 10 randomized controlled trials showed that antibiotic use increased height by 0.04 cm/month.[11] Another study demonstrated that randomized administration of

For peer review only - http://bmjopen.bmj.com/site/about/guidelines.xhtml

azithromycin to asymptomatic young children in Ethiopia was associated with a halving of mortality,[12] suggesting overall effects of suppressing bacterial pathogens in areas with high rates of infectious disease. However results from individual studies of azithromycin mass drug-administration on growth have not shown consistent results.[13] Among children with cryptosporidial infection in Zambia, treatment with nitazoxanide resulted in reduced mortality, while linear growth was not assessed.[14] Therefore, the role of these antimicrobials for linear growth remains unclear.

Nicotinamide to improve childhood growth

Another potential means of improving growth is by targeting the tryptophan-kynurenine-niacin pathway (Figure 1). In this pathway, dietary tryptophan is absorbed and then metabolized by indoleamine 2,3-dioxygenase (IDO) to kynurenine and later to nicotinic acid, a key precursor to NAD+. The importance of this pathway has been supported by recent work identifying mutations in the NAD+ pathway as a cause of birth defects in humans; in animal models with these mutations, birth defects were prevented via supplementation with niacin.[15] Deficiencies in this pathway have been noted in areas where diets are heavily maize-based, leading to pellagra. Infants at the Haydom site in MAL-ED were noted to have inadequate dietary intake of multiple vitamins[16]; while deficiency in niacin was not assessed, individuals in the region around Haydom eat a diet based heavily on maize, with potentially low bioavailability of tryptophan. Basic science models have demonstrated that tryptophan deficiency is associated with reduced growth velocity.[17]

Abnormalities in the tryptophan-kynurenine-niacin pathway are also associated with intestinal inflammation, including during response to intestinal pathogens.[18 19] In animal models, an inability to transport tryptophan into enterocytes results in diarrhea, while provision of nicotinamide (a form of niacin) partly restores function of mTOR in enterocytes, increasing secretion of antimicrobial peptides and reducing diarrhea,[18 19] suggesting effects on diarrheal

For peer review only - http://bmjopen.bmj.com/site/about/guidelines.xhtml

pathogens. IDO activity is increased during inflammation, including in inflammatory bowel disease[20 21] and during infections;[22] this appears to be a maladaptive in most cases, as kynurenine has potential immunosuppressive effects.[23 24] In MAL-ED, children at the Haydom site with higher ratios of kynurenine:tryptophan were noted to have poorer linear growth.[24] Given that 1) children in the Haydom area have a maize-based diet low in tryptophan, a key precursor to niacin/nicotinamide and NAD+, 2) preclinical studies demonstrate improvements in antimicrobial peptides and reduced diarrhea with nicotinamide supplementation, 3) an increase in nicotinamide as a precursor of NAD+ should cause reduced need for the tryptophan-kynurenine-niacin pathway, reducing activity of IDO and thereby reducing kynurenine, and 4) higher kynurenine:tryptophan is associated with poor growth, we reasoned that intervention with nicotinamide among children in this region would improve linear growth. However, because growth is a long-term process and nicotinamide is a water-soluble vitamin without a mechanism for on-going storage, we reasoned that an intervention with nicotinamide would need to be given over a long period of time.

Study hypotheses

We hypothesized that among children with a high degree of stunting and enteric pathogen burden in this region, intervention with 1) antimicrobials and/or 2) nicotinamide would increase linear growth. We further set out to interrogate the potential mechanisms behind these effects. In the case of antimicrobial intervention, we hypothesize that intervention with azithromycin and nitazoxanide (relative to placebo) would result in a decrease in enteropathogen burden that would be associated with an increase in growth. In the case of nicotinamide (relative to placebo), we hypothesize that intervention will result in a reduction in the kynurenine:tryptophan ratio, an increase in availability of nicotinamide for NAD+ synthesis and associated increases in growth.

For peer review only - http://bmjopen.bmj.com/site/about/guidelines.xhtml

OBJECTIVE

The primary objectives of this randomized factorial, double-blind, placebo-controlled trial are to determine if interventions with 1) antimicrobials and 2) nicotinamide are associated with increased length-for-age z-score (LAZ, relative to placebo-treated children) by age 18 months. Specifically, the antimicrobial intervention consists of azithromycin administered as a single dose at age 6, 9, 12, and 15 months and nitazoxanide as a 3-day course at age 12 and 15 months (with both antimicrobials randomized together). The nicotinamide intervention consists of nicotinamide 250 mg daily administered daily by mouth to the child's mother during breast feeding 0-6 months (and thus passed on to the child via breast milk[25]) and then 100 mg administered daily by mouth to the child during months 6-18. These interventions are delivered in a factorial manner such that children receive both interventions, either intervention alone or neither intervention.

Because multiple prior large-scale studies of other preparations of vitamins and antibiotics have failed to show effects on linear growth, we have multiple secondary outcomes to interrogate additional physiological effects of the interventions—potentially helping to identify underlying mechanisms. These secondary outcomes include measures of inflammation, intestinal bacterial content (pathogens and otherwise), and measures of metabolism, development and health.

SETTING

Haydom is a rural area in north-central Tanzania, approximately 300 km west of Arusha, the nearest urban center.[4] There is poor infrastructure for roads and other resources, and the local population of approximately 20,000 depends primarily on subsistence agriculture. Haydom Lutheran Hospital (HLH) is the central hub for the area and has a strong relationship with the Hadyom community. Healthcare in the area relies both on HLH and reproductive child health system (RCHS) with 38 outreach clinics across 74 villages, where children are followed monthly

For peer review only - http://bmjopen.bmj.com/site/about/guidelines.xhtml

for weight-checks and immunizations. Given the low HIV seroprevalence (<2%) among mothers of childbearing age, HIV testing is not performed I the current study. Because of its elevation (1,700 meters), Haydom is not a high-risk area for malaria, with almost no *Plasmodium falciparum* in the region. Among the families followed in MAL-ED, 92.3% lived in houses with mud floors, 0% had electricity, and 67.9% obtained their water from an unprotected well or surface water.[4] Further estimates of the low socioeconomic status of the area have been published previously.[4]

METHODS AND ANALYSIS (Standard Protocol Items for Randomized Trials)

Eligibility

Participants are eligible if the mother is ≥18 years and the child is age ≤14 days and was born in the Haydom catchment area (defined for study purposes as 25 km radius of Haydom Lutheran Hospital).

Exclusion criteria are as follows: maternal inability to adhere to the protocol, multiple gestation, significant birth defect or neonatal illness, weight <1,500 g at enrollment (age ≤ 14 days), lack of intent to breastfeed infant and plan to move from area within the next 18 months.

Recruitment

Recruitment occurs via survey by field teams of surrounding villages and via input from local community healthcare workers. Pregnant women are identified and informed regarding the study. Pregnant women in the third trimester may provide informed consent for the study but are not enrolled until the initial assessment visit of the infant to determine eligibility (which includes ascertainment of infant weight). If informed consent is not obtained during pregnancy, it is obtained at enrollment. The general timeline of study procedures is shown in Table 1.

For peer review only - http://bmjopen.bmj.com/site/about/guidelines.xhtml

Enrollment

Infant/mother pairs are enrolled during the initial study visit to their homes between birth and day of life 14, after eligibility criteria are confirmed, including measurement of infant weight which should be 1.5 or above. Informed consent is provided at this time, if it was not obtained during pregnancy. Eligibility (besides current weight) is confirmed and the mother/child pair is randomized in the study.

Randomization

Randomization occurs at the time of enrollment. Block randomization was used in permuted blocks of eight for the four intervention combinations (antimicrobial + nicotinamide, antimicrobial alone, nicotinamide alone, all placebo). Study teams are assigned permuted blocks before initial study visits to sequentially allocate randomization status to participants based on study identification number assigned prior to the initial study visit.

Intervention

As a factorial study design, the two intervention domains (antimicrobial and nicotinamide) are randomized separately. The antimicrobial intervention consists of two medications that are randomized together: azithromycin and nitazoxanide (vs. placebos of both). Azithromycin 20 mg/kg (vs. placebo) (both manufactured by Universal Corp, Kenya) is administered by study personnel at months 6, 9, 12 and 15 as a single dose of azithromycin (200 mg/5 mL suspension by mouth). This timing was selected (versus earlier initiation) because while children have enteropathogen carriage before 6 month in this area,[8] carriage of azithromycin-susceptible pathogens such as Campylobacter are not highly prevalent, and the decline in LAZ is steeper in the 6-18-month window compared to the 0-6 month window.[5] The nitazoxanide (vs. placebo) (both manufactured by Romark, Florida, USA) is administered as a course of nitazoxanide (100 mg/5 mL) 100 mg twice daily for three days. This timeline was

For peer review only - http://bmjopen.bmj.com/site/about/guidelines.xhtml
For peer review only

selected because nitazoxanide is not approved for use below 12 months. The first of these nitazoxanide doses is administered by the study personnel and the remaining doses are administered at home by the child's family. Families are asked at a subsequent visit about whether they completed the course of nitazoxanide.

The nicotinamide (vs. placebo) (both manufactured by Vita-gen, New York, USA) is delivered as a daily dose throughout the study. During the first 6 months of life, this is delivered to the child via breastmilk. This is performed because of the lack of availability of a liquid preparation of nicotinamide and to emphasize the importance of exclusive breast feeding (i.e., that intake beyond breast milk is not needed during the first 6 months of life). Mothers take nicotinamide 250 mg tablets, one tablet by mouth daily. Prior studies have demonstrated that nicotinamide is present in breast milk in quantities that are associated with serum levels.[25] Haydom-specific data from MAL-ED demonstrated a high prevalence (96%) of breast feeding until children were 6 months old.[5] Because the prevalence of exclusive breastfeeding was low in the area around Haydom during MAL-ED, the current study includes a breast-feeding encouragement and support initiative, utilizing nurses who have received specialized training in breast feeding; these nurse work with the local community healthcare workers in home visits to participating families in the first 6 months of the study. Their message is that exclusive breast feeding is better for mother and baby (highlighting a variety of reasons) and that if the family chooses not to breast feed, that providing breast milk before any complimentary foods will allow the child to receive a greater amount of the needed nutrition—including a greater amount of the nicotinamide ingested by the mother. The lack of intent to breast feed is an exclusion criterion at the time of enrollment; however, if the mother dies or discontinues breast feeding, the child subsequently would not receive nicotinamide/placebo until age 6 months.

From months 6-18 the child receives sachets of nicotinamide 100 mg by mouth daily. Mothers are instructed to mix the nicotinamide powder with a small volume of age-appropriate food (e.g. infant cereal) to give to the child.

For peer review only - http://bmjopen.bmj.com/site/about/guidelines.xhtml

For both the maternal nicotinamide pills and the children's nicotinamide sachets, doses are given as a 60-day supply every two months. When a new supply is provided, the family is asked for the remainder of doses not consumed the prior 60 days. These unused doses are counted and recorded by pharmacy staff to determine adherence.

Data collection

Questionnaires

Study staff use standardized questionnaires designed for the study, in many cases adapted from questionnaires used in the MAL-ED study. These include questions asked at each monthly visit regarding breastfeeding, use of complementary foods, childhood illness and treatment (including whether an antibiotic was used and if so which one), types of food sources given, and food insecurity. Additional questionnaires are asked at other specific time periods, including aspects of the child's home environment (completed at the first month's visit) and the vaccines that the child has received (completed at the 12-month visit).

Anthropometry measures

All field team members are trained in the accurate measurement of anthropometry indices. The child's weight is measured at the initial visit and at each monthly visit through age 18 months. The child's length and head circumference are measured at the initial visit and every three visits thereafter through 18 month. The mother's weight and height are measured at month 10-11. Quarterly, weights are measured using a digital scale that undergoes quality assessments weekly. Infants are weighed on an infant tray with the infant nude or wearing a dry diaper or standing, when able. In between these quarterly weight measures, weights are measured monthly using hanging spring scales that are zeroed before each use and assessed for accuracy weekly. Lengths are measured quarterly by two field team members with the child lying flat on a measuring board with a fixed, perpendicular board against which the head is

For peer review only - http://bmjopen.bmj.com/site/about/guidelines.xhtml

placed and a second adjustable perpendicular board is placed against the feet. Two measurements are obtained; if these are within 2 mm, the average of the two is used; if these are not within 2 mm, a third measurement is obtained and the average of the two closest measurements is used. Head circumference is measured quarterly by placing a non-distensible tape around the child's head just above the eyebrows, above the ears, and around the biggest part of the back of the head. Mid-upper arm circumference is measured quarterly by using the same measuring tape at the mid-point of the upper arm segment. Maternal weight is measured using a standing scale. Maternal height is measured using a portable stadiometer. For quality assurance, 5% of infant weights, lengths, and head circumferences are measured by a supervisor skilled in measurement to compare to the field team's measurement.

Developmental assessments

To assess maternal-child interaction, an important predictor of early childhood development and health,[26 27] The Observation of Maternal Child Interaction (OMCI)[28] assessment is collected at 12 months of age. This observation includes a 5 minute observation of mother and child interacting over a picture book, with ratings given for positive affect, verbal statements, sensitivity, scaffolding, language stimulation, focus and mutual enjoyment, among others.

At 18 months, developmental assessment is performed by trained assessors using two standardized scores, the Malawi Developmental Assessment Tool (MDAT)[29] and the Bayley Scales of Infant and Toddler Development, cognitive assessment (Bayley).[30] The MDAT is a validated score that tests multiple childhood development domains, including gross motor, fine motor, language and social development that has been validated in this age range in Malawi and intended for use in rural sub-Saharan settings. Briefly, this assessment was designed to be a culturally-relevant method for examining child development across multiple domains using

For peer review only - http://bmjopen.bmj.com/site/about/guidelines.xhtml

objects and tools typically available in rural or lower-resource settings. Both direct assessment and caregiver report of skills go into the score.

The Bayley cognitive assessment involves a series of tasks presented to the infant, which assess for play, reasoning, and mental representation. This is a commonly used assessment for early childhood and has been used in many research sites around the world.[6 31]

Specimen collection

Breast milk (months 1 and 5): Participating mothers express a maximal volume of breast milk from one breast (up to 40 mL), using hand expression under low-light conditions. Breast milk will be collected into foil-covered sterile containers and transported on ice to the central laboratory center within 8 hours for archiving of whole milk at -80 degrees.

Blood (months 12 and 18; in a subset months 2 and 8): Blood is drawn from an experienced phlebotomy team using a butterfly needle and syringe and placed in micro-containers for transport to the laboratory for processing into serum and plasma and storage at -80 degrees. In the case of laboratory studies to assess the safety of nicotinamide administration (performed on the first 100 children enrolled), a whole blood specimen is immediately tested in the clinical laboratory at Haydom Lutheran Hospital for complete blood count (CBC).

Stool (months 6, 9, 12, 15, and 18): Stool is collected in diapers that are provided just prior to the stool collection date. Mothers or caregivers are instructed to collect approximately 3–4 spoonfuls of stool and place it within 1 hour into a labeled stool container, seal in a plastic bag, and place inside a transport box with cold packs. The stool collection will be timed prior to a planned study visit, such that the field-worker can collect the sample and the laboratory process the specimen shortly thereafter. Stool samples are then stored at -80 degrees. A subset of specimens, prior to freezing will be cultured for E.coli and screened for antibiotic resistance

For peer review only - http://bmjopen.bmj.com/site/about/guidelines.xhtml

(AMR). This is performed as surveillance for safety and will be performed on a randomly-selected 25 participants per intervention allocation group at 12 and 15 months.

Urine (months 6, 12, and 18): Urine is collected by placing a bag over the child's genitalia at the beginning of a study visit. The child is given adequate hydration, via breast feeding if possible. Collected urine is then transferred in a sterile container that is stored on ice prior to transport to the research site and then stored at -80 degrees.

Saliva (maternal and infant at month 12): Maternal and child saliva is collected into a vial containing storage reagent and stored at -80 degrees for genomic assessment.

Breath (subset of participants at months 6, 9 and 12): Glucose hydrogen breath testing is performed in a subset of children (the last 400 children enrolled) as an assessment of small intestine bacterial overgrowth (SIBO),[32] which has been associated with intestinal inflammation and linear growth shortfall.[33] Children are fasted for 2 hours prior to the initiation of the breath test. They are then given 1 g/kg actual body weight of a glucose dissolved in a 1 g/5ml solution. Following ingestion of the standardized glucose solution, participants have breath collected via the Quintron pediatric breath collection bag system every 20 minutes for 2 hours. This non-invasive procedure involves the child breathing into an anesthesia mask connected to a one-way flutter valve and collection bag. Breath samples are extracted from the collection bag using a syringe and stopcock and placed into specialized vacutainer glass vials. These vials are then transported to our research facility at Haydom Lutheran Hospital for assessment.

Laboratory procedures and storage

For peer review only - http://bmjopen.bmj.com/site/about/guidelines.xhtml

Upon receipt of the samples listed above, the laboratory team at the research site prepares aliquots of samples for storage at -80 degrees for local testing or shipping to additional testing facilities as shown below.

Breast milk

Rationale

During the first 6 months of participation, the only means of the child receiving intervention is through maternal delivery of nicotinamide in breast milk. However, nicotinamide *in vivo* is both derived from tryptophan and converted to multiple other vitamers.[34] As such, we will assess content of nicotinamide, vitamers and tryptophan in breast milk at two time points during the intervention.

Maternal breast milk samples collected at months 1 and 5 will be tested at the United States Department of Agriculture laboratory at the University of California – Davis. Planned LC-MS analyses includes nicotinamide, thiamin, riboflavin, flavin adenine dinucleotide, pyridoxal, pyridoxine, biotin and pantothenic acid concentrations, and on a random subset (n=600 samples) niacin vitamer, and free and total tryptophan concentrations.

Blood

Rationale

Blood testing will include assessment of multiple measures both to verify safety of the interventions and interrogate the effects of the intervention on physiological processes related to linear growth. Safety assessment includes testing of general tests of health with basic metabolic panel, liver function and enzyme testing, and complete blood count. This will assess for differences between intervention groups in measures of kidney, liver and hematopoetic health. Additional tests to interrogate the physiologic effects of the intervention include

For peer review only - http://bmjopen.bmj.com/site/about/guidelines.xhtml

assessment of metabolic effects (using metabolomics) and assessment of levels of factor related to growth (insulin-like growth factor-1 [IGF-1] and collagen X).

Complete blood count (CBC): To assess general safety of the nicotinamide dosing in the first 100 infants at months 2 and 8. CBC is performed at Haydom Lutheran Hospital clinical on a Swelab Alfa hematology analyzer (Boule Diagnositics, Stagna, Sweden; this machine has quality control assessed daily).

Basic metabolic panel (BMP), aminotransferases and bilirubin: To assess general safety of the nicotinamide dosing in the first 100 infants at months 2 and 8, additional laboratory measures will be performed on serum samples at the Kilimanjaro Christian Medical Center. BMP, bilirubin, alanine aminotransferase (ALT) and aspartate aminotransferase (AST) will be assessed using a Cobas Integra 400 Plus biochemistry analyzer (Roche Diagnostics, Germany), according to the manufacturer's instructions.[35]

Insulin-like growth factor-1 (IGF-1) and high-sensitivity C-reactive protein (hsCRP): To interrogate these interventions for changes in growth factor and low-grade systemic inflammation, respectively, IGF-1 and hsCRP will be tested at 12 and 18 months at the UVa Center for Research in Reproduction Ligand Core Laboratory using an Immulite 2000.

Collagen X: We will assess for intervention-associated changes in collagen X (a biomarker of rapid skeletal growth) using an ELISA developed at Shriners Hospital for Children in Portland, OR, and Oregon Health and Science University.[36]

Metabolomics: The tryptophan-kynurenine-niacin pathway will be assessed at 12 and 18 months via metabolomics analyses of serum and plasma using ^{1}H nuclear magnetic resonance

For peer review only - http://bmjopen.bmj.com/site/about/guidelines.xhtml

(NMR) spectroscopy and ultra-performance liquid chromatography-mass spectrometry (UPLC-MS) at Imperial College, London. Assessments include the following: 1) metabolites of the tryptophan-kynurenine-niacin pathway to assess for (among other items) changes in the tryptophan:kynurenine ratio in the setting of nicotinamide treatment;[24] 2) plasma bile salts, which have implications for risk of infection;[37] 3) untargeted metabolomics analysis, providing information on the global metabolic status of the individual including pathways related to the metabolic activity of the gut microbiota and their biochemical interactions with the host;[38] and untargeted UPLC-MS based lipidomics to assess for differences in lipid metabolism by nutritional status.

Stool

Stool testing will include assessment of the following:

Microbiome: Stool bacterial content will be assessed at in a subset of 200 participants at the University of Maryland. Total stool DNA will be isolated by bead beating method. A library will be created each specimen. The libraries will be multiplexed and sequenced by Illumina HiSeq. The resulting reads will be cleaned, binned by specimen ID, separated in operational taxonomic units (OTUs) by DNAclust[39] and compared using metagenomeseq. Metagenomeseq normalizes the data and introduces a correction factor to account for under-sampling of OTUs. The normalized data will be compared using odds ratios and multiple t-tests, with p-values adjusted for multiple testing.

Taqman Array Cards (TAC): Pathogen burden will be assessed using TAC cards (Figure 2) as performed previously.[40 41 42] This will be performed at in the laboratory at Haydom Global Health Research Center using. Briefly, stool samples undergo nucleic acid extraction via the QIAamp Fast Stool DNA Mini kit (Valencia, CA). Nucleic acids are mixed with AgPath-ID One-Step RT-

For peer review only - http://bmjopen.bmj.com/site/about/guidelines.xhtml

PCR buffer (Thermo Fisher) and nuclease free water to a 100ul final volume and run on a custom-designed TAC[40]:

Antibiotic resistance: We will perform stool bacterial culture on a subset of participants to monitor for development of antimicrobial resistance, which is a valid concern of any mass antimicrobial administration. Briefly, stool will be cultured on MacConkey agar and 5 lactose fermenting E. coli colonies pooled and tested for AMR using disc diffusion to screen for ESBL, AmpC, carbapenemase and susceptibility to antibiotics commonly in use locally. Azrythromycin susceptibility will be tested by E-Test.[43]

Myeloperoxidase: As an assessment of stool inflammation, myeloperoxidase will be assessed at months 6, 12 and 18. Testing will be performed at the Global Health Research Center laboratory in Haydom using a commercially-available assay (Alpco, Salem, NH).

Urine

Urine samples at 6, 12 and 18 months will assessed alongside serum and plasma samples at Imperial College, London for untargeted metabolomics analysis by NMR spectroscopy and UPLC-MS, as described above.[38]

Saliva

For a more complete assessment of these interventions on intestinal inflammation in this setting, we will test mothers and children for secretor status of fucosyltransferase 2 (*FUT2*), a regulator of blood group antigens on the intestinal mucosa, as non-secretor status has been associated with infections[44] and other forms of colitis.[45] DNA for mothers and children will be extracted from salivary samples at UVa and *FUT2* genotype will be determined by dot blot assays in a hierarchical method based largely on Lewis Type a and b antigens. Lewis double

For peer review only - http://bmjopen.bmj.com/site/about/guidelines.xhtml

negatives (5-7%) are not able to be characterized as for their secretor status and so will be typed for A, B, and H antigens to allow for phenotypic characterization of all samples.[46]

Breath

To interrogate these interventions for changes in SIBO, a pathophysiological process associated with poor linear growth, we will assess hydrogen and methane quantity of breath samples in a subset of 200 participants. Breath samples collected in glass vials will be extracted and analyzed using a Quintron BreathTracker SC breath chromatograph (Quintron Inc, Milwaukee, WI USA).[33] Data on both hydrogen and methane production will be collected.

Data management and confidentiality

Data are collected onto paper forms, which are then quality checked before being double-entered by two different study team data clerks independently at the research site into a secure web-based system (Multi-Schema Information Capture, MuSIC) managed by UVa Department of Public Health Sciences. Any discrepancies in this double-data entry are reconciled by assessing the original paper form.

Data analysis

Primary endpoint

The primary outcome for the overall study is LAZ at 18 months. For each of the intervention domains we will compare this outcome as a t-test between those randomized to receive the intervention vs. those who did not. This will be performed using a modified intent-to-treat analysis, assessing participants who complete the study and have a measurement of LAZ at 18 months.

Secondary endpoints

For peer review only - http://bmjopen.bmj.com/site/about/guidelines.xhtml

Each of the intervention domains has its own set of secondary outcomes as well (see Table 2), and these will be compared between those who receive the intervention and those who do not. This will initially be performed using the same modified intent-to-treat analysis assessing those with the individual outcome of interest. This will be performed as t-tests for continuous outcomes and chi square tests for binary outcomes.

Secondary analysis for all endpoints (including LAZ at 18 months) will also be performed using per-protocol treatment, defined as participants meeting the following criteria:

- Breastfed through age 6 months
- Received all doses of azithromycin
- Received initial dose of nitazoxanide
- Received at least 50% of nicotinamide doses as measured by pill- and sachet counting.
- Have the outcome of interest measured.

Blinding

The study is being performed in a double-blinded manner. Individual study medications were purchased from the same manufacturer as their corresponding placebo, and placebos were designed to be similar in appearance and taste. Study personnel received the medications and placebos merely labeled as "A" and "B," with the key to this allocation provided to one individual at UVa not otherwise associated with the study. Blinding will be maintained throughout the study unless adverse events occur in significantly disproportionate amounts by intervention allocation status. Allocation status will otherwise only be revealed following completion of collection of data and samples.

Data and safety monitoring

For peer review only - http://bmjopen.bmj.com/site/about/guidelines.xhtml

A data and safety monitoring board has been formed to review data regarding safety of these interventions. This board contains four experts and physicians from Tanzania, the US and the United Kingdom with specialties in gastroenterology, global health, pediatrics, and statistics to assist with reviewing data related to adverse events, health outcomes and surveillance cultures evaluating for antibiotic resistance. Adverse event data are reported monthly by treatment allocation group (in a blinded fashion). The DSMB makes recommendations regarding ongoing safety of the interventions and any need to be un-blinded due to differences in adverse events between treatment allocation group and can recommend discontinuation of any of the intervention arms.

Statistical power

The primary outcome is difference between intervention groups in LAZ at 18 months. The sample size was calculated to provide sufficient power for testing the main effects using separate t-tests for each of the two interventions in this 2 x 2 factorial design, using a standard deviation of 1.03 HAZ at 18 months. If each main effect were tested at the 5% levels, as is customary in the analysis of factorial studies,[47 48] with 270 participants per group, there is 80% power for a difference in LAZ of 0.176. This is within the range of improvement of an increase of 0.16 seen in a prior study after 12 months of micronutrient supplementation,[49] though in Haydom the growth and deficiencies are more severe than most areas, thus the potential for observing a greater effect size. The interaction between main effects of these interventions will then be estimated and tested as a secondary analysis using a 2-way ANOVA. If we were to take a more conservative approach and use a significance level of 2.5% for each of the main effects, with 270 subjects per group, we would still have 80% power to detect an average LAZ increase of 0.193. Finally, if we were to adjust the significance to include the test for the interaction, the F-test for the main effect in a 2-way ANOVA has 80% power with a two-sided significance level of 1.67%, when the main effect of the intervention is to increase the mean LAZ

For peer review only - http://bmjopen.bmj.com/site/about/guidelines.xhtml

by 0.203. All of these are reasonable changes in LAZ to observe for in the Haydom population, given the considerable demonstrated mean deficit in growth. Adjusting for 10% dropout, the total sample size required is 1188. Within our recruitment radius >2507 births occur yearly. We will recruit during a 12-month window. In our extensive research experience at the Haydom MAL-ED site, we observed 0% refusal to participate and a high 81% retention rate by 4 years of follow-up during MAL-ED (and, thus, a study of shorter duration may have improved follow-up). Based on this, we anticipate a high rate of recruitment (>90%) and 18-month retention (>90%). Therefore, accounting for drop-out, we anticipate achieving 1188 mother/child dyads divided between intervention and control, leaving us adequate power to determine differences in LAZ.

Statistics

In addition to use of ITT for the central analysis (t-tests) and the standard 2-way ANOVA to assess main effects and the interaction, we will perform several sensitivity analyses to assess for influences from children who dropped out or died during the study. These will include assessments of last-measurement carried forward and assumptions about those who dropped out being taller or shorter than the mean. Additional secondary analyses will include assessing hazard ratios for events such as mortality and hospitalization, including time to event analysis.

Study timeline

Enrollment began 5 September 2017 and is planned to continue through 5 September 2018. Children will be followed until age 18 months, resulting in a planned completion in February 2020. Blinding will be maintained until all final study visits are completed. The primary analysis will be performed once data collection is complete for the variables involved. To reduce intra-assay variability, the majority of laboratory testing will be batched and performed once complete samples are present.

For peer review only - http://bmjopen.bmj.com/site/about/guidelines.xhtml
For peer review only

Potential challenges and limitations

Potential challenges include adequate recruitment of target participant numbers within the one-year study window. Should this be true, we will consider extending the recruitment window. Maintaining adherence of study medication, particularly the daily doses of nicotinamide to breast-feeding mothers during months 0-6, and infants during months 6-18, may be a challenge in a setting in which few individuals are accustomed to taking daily medication or nutritional supplements. To protect against adherence challenges, we performed a pilot study beforehand, identifying potential barriers to adherence and resulting in steps to remind mothers frequently during the first month of the study, with follow-up home visits by the field team and by local community healthcare workers.

ETHICS AND DISSEMINATION

Regulatory authorities

ELICIT has received approval from Tanzania's National Institute of Medical Research (NIMR, reference number HQ/R.8a/Vol.IX/2424, approved 3 March 2017), the University of Virginia Health Sciences Research Institutional Review Board (HSR-IRB, reference #19465, approved 19 January 2017), and the Tanzanian Food and Drug Administration (TFDA, reference number TFDA0017/CTR/0005/02, approved 5 July 2017). All research procedures are conducted in accordance with the Declaration of Helsinki and according to International Conference on Harmonisation Good Clinical Practice guidelines. All participant mothers provide informed consent. An external monitor (FHI360) evaluated the site prior to initiation of the study and performs yearly site visits and assessments thereafter.

Patient and Public Involvement

For peer review only - http://bmjopen.bmj.com/site/about/guidelines.xhtml

Patients and the public were not directly involved in the planning of this study. Results will be communicated to participants via community meetings with local leaders.

Dissemination

This trial has been registered at ClinicalTrials.gov (#NCT03268902). Scientific reports resulting from this study will be made publicly available. Following completion of data accrual, data will be made publicly available in an online database. Data will be presented at major conferences such as those of the American Society of Tropical Medicine and Hygiene, the Pediatric Academic Society, the Endocrine Society, and the African Academy of Sciences. Scientific articles will be published with open-access availability at major scientific journals.

For peer review only

Authors' contributions

MDD, JPM, RJS, JM, JG, ES, EH and EM conceived of this trial and developed this study protocol. EM is the Principal Investigator; MDD is the UVa Principal Investigator. SJ is the Clinical Research Coordinator; AW is the UVa Project Manager. JPM, JG and EH developed procedures related to stool testing for pathogens and antibiotic resistance. JS developed procedures related to metabolomics testing. JRD developed procedures related to testing for small intestinal bacterial overgrowth. JMM developed procedures related to the breastfeeding support of mothers for community, field and nursing staff. RJS and ES developed procedures related to developmental testing. JG oversees all sample processing and laboratory procedures in Haydom. All authors contributed to the development of this manuscript and/or study procedures and to reading and approving the final version for publication.

For peer review only - http://bmjopen.bmj.com/site/about/guidelines.xhtml

Funding statement

This work was supported by the Bill and Melinda Gates Foundation, OPP1141342.

Role of the sponsor and funder

The funder contributed input into the design of the study. Neither the sponsor nor the funder had input into the preparation of this report for publication. The sponsor has ultimate authority into these activities.

Acknowledgments

Romark donated the nitazoxanide.

Competing interests statement

None of the authors or study co-investigators have any competing interests to declare.

REFERENCES

1. Grantham-McGregor S, Cheung YB, Cueto S, et al. Child development in developing countries 1 - Developmental potential in the first 5 years for children in developing countries. *Lancet* 2007;369(9555):60-70.
2. Prendergast AJ, Humphrey JH. The stunting syndrome in developing countries. *Paediatrics and International Child Health* 2014;34(4):250-65. doi: 10.1179/2046905514y.0000000158
3. Kosek MN, Investigators M-EN. Causal Pathways from Enteropathogens to Environmental Enteropathy: Findings from the MAL-ED Birth Cohort Study. *EBioMedicine* 2017;18:109-17. doi: 10.1016/j.ebiom.2017.02.024 [published Online First: 2017/03/08]
4. Mduma ER, Gratz J, Patil C, et al. The etiology, risk factors, and interactions of enteric infections and malnutrition and the consequences for child health and development study (MAL-ED): description of the Tanzanian site. *Clin Infect Dis* 2014;59 Suppl 4:S325-30. doi: 10.1093/cid/ciu439
5. MAL-ED_Network_Investigators. Childhood stunting in relation to the pre- and postnatal environment during the first 2 years of life: The MAL-ED longitudinal birth cohort study. *PLoS Med* 2017;14(10):e1002408. doi: 10.1371/journal.pmed.1002408 [published Online First: 2017/10/25]
6. Scharf RJ, Rogawski ET, Murray-Kolb LE, et al. Early Childhood Growth and Cognitive Outcomes: Findings from the MAL-*ED Study*. *Maternal and Child Nutrition* 2017;in press

For peer review only - http://bmjopen.bmj.com/site/about/guidelines.xhtml

7. Krebs NF, Lozoff B, Georgieff MK. Neurodevelopment: The Impact of Nutrition and Inflammation During Infancy in Low-Resource Settings. *Pediatrics* 2017;139(Suppl 1):S50-S58. doi: 10.1542/peds.2016-2828G
8. Platts-Mills JA, Babji S, Bodhidatta L, et al. Pathogen-specific burdens of community diarrhoea in developing countries: a multisite birth cohort study (MAL-ED). *Lancet Glob Health* 2015;3(9):e564-75. doi: 10.1016/S2214-109X(15)00151-5 [published Online First: 2015/07/19]
9. MAL-ED_Network_Investigators. Relationship between growth and illness, enteropathogens and dietary intakes in the first 2 years of life: findings from the MAL-ED birth cohort study. *BMJ Global Health* 2017;2(4):e000370.
10. Rogawski ET, Bartelt LA, Platts-Mills JA, et al. Determinants and Impact of Giardia Infection in the First 2 Years of Life in the MAL-ED Birth Cohort. *J Pediatric Infect Dis Soc* 2017;6(2):153-60. doi: 10.1093/jpids/piw082
11. Gough EK, Moodie EEM, Prendergast AJ, et al. The impact of antibiotics on growth in children in low and middle income countries: systematic review and meta-analysis of randomised controlled trials. *Bmj-British Medical Journal* 2014;348 doi: 10.1136/bmj.g2267
12. Porco TC, Gebre T, Ayele B, et al. Effect of Mass Distribution of Azithromycin for Trachoma Control on Overall Mortality in Ethiopian Children A Randomized Trial. *Jama-Journal of the American Medical Association* 2009;302(9):962-68.
13. Amza A, Kadri B, Nassirou B, et al. A cluster-randomized controlled trial evaluating the effects of mass azithromycin treatment on growth and nutrition in Niger. *Am J Trop Med Hyg* 2013;88(1):138-43. doi: 10.4269/ajtmh.2012.12-0284
14. Amadi B, Mwiya M, Musuku J, et al. Effect of nitazoxanide on morbidity and mortality in Zambian children with cryptosporidiosis: a randomised controlled trial. *Lancet* 2002;360(9343):1375-80. doi: 10.1016/S0140-6736(02)11401-2
15. Shi H, Enriquez A, Rapadas M, et al. NAD Deficiency, Congenital Malformations, and Niacin Supplementation. *N Engl J Med* 2017;377(6):544-52. doi: 10.1056/NEJMoa1616361
16. Chen C, Mitchell NJ, Gratz J, et al. Fumonisin, Not Aflatoxin, is Associated with Growth Impairment in Tanzanian Children. *Environmental International* 2018;in press
17. Le Floc'h N, Otten W, Merlot E. Tryptophan metabolism, from nutrition to potential therapeutic applications. *Amino Acids* 2011;41(5):1195-205. doi: 10.1007/s00726-010-0752-7
18. Perlot T, Penninger JM. ACE2 - from the renin-angiotensin system to gut microbiota and malnutrition. *Microbes Infect* 2013;15(13):866-73. doi: 10.1016/j.micinf.2013.08.003
19. Hashimoto T, Perlot T, Rehman A, et al. ACE2 links amino acid malnutrition to microbial ecology and intestinal inflammation. *Nature* 2012;487(7408):477-81. doi: 10.1038/nature11228 [published Online First: 2012/07/25]
20. Wolf AM, Wolf D, Rumpold H, et al. Overexpression of indoleamine 2,3-dioxygenase in human inflammatory bowel disease. *Clin Immunol* 2004;113(1):47-55. doi: 10.1016/j.clim.2004.05.004
21. Gupta NK, Thaker AI, Kanuri N, et al. Serum analysis of tryptophan catabolism pathway: correlation with Crohn's disease activity. *Inflamm Bowel Dis* 2012;18(7):1214-20. doi: 10.1002/ibd.21849 [published Online First: 2011/08/05]
22. Favre D, Mold J, Hunt PW, et al. Tryptophan catabolism by indoleamine 2,3-dioxygenase 1 alters the balance of TH17 to regulatory T cells in HIV disease. *Sci Transl Med* 2010;2(32):32ra36. doi: 10.1126/scitranslmed.3000632
23. Mayneris-Perxachs J, Lima AA, Guerrant RL, et al. Urinary N-methylnicotinamide and β-aminoisobutyric acid predict catch-up growth in undernourished Brazilian children. *Sci Rep* 2016;6:19780. doi: 10.1038/srep19780

For peer review only - http://bmjopen.bmj.com/site/about/guidelines.xhtml

24. Kosek MN, Mduma E, Kosek PS, et al. Plasma Tryptophan and the Kynurenine-Tryptophan Ratio are Associated with the Acquisition of Statural Growth Deficits and Oral Vaccine Underperformance in Populations with Environmental Enteropathy. *Am J Trop Med Hyg* 2016;95(4):928-37. doi: 10.4269/ajtmh.16-0037 [published Online First: 2016/08/08]
25. Allen LH, Hampel D, Shahab-Ferdows S, et al. Antiretroviral therapy provided to HIV-infected Malawian women in a randomized trial diminishes the positive effects of lipid-based nutrient supplements on breast-milk B vitamins. *Am J Clin Nutr* 2015;102(6):1468-74. doi: 10.3945/ajcn.114.105106
26. Obradović J, Yousafzai AK, Finch JE, et al. Maternal scaffolding and home stimulation: Key mediators of early intervention effects on children's cognitive development. *Dev Psychol* 2016;52(9):1409-21. doi: 10.1037/dev0000182 [published Online First: 2016/08/08]
27. Walker SP, Wachs TD, Grantham-McGregor S, et al. Child Development 1 Inequality in early childhood: risk and protective factors for early child development. *Lancet* 2011;378(9799):1325-38. doi: 10.1016/s0140-6736(11)60555-2
28. Rasheed MA, Yousafzai AK. The development and reliability of an observational tool for assessing mother-child interactions in field studies- experience from Pakistan. *Child Care Health Dev* 2015 doi: 10.1111/cch.12287
29. Gladstone M, Lancaster GA, Umar E, et al. The Malawi Developmental Assessment Tool (MDAT): the creation, validation, and reliability of a tool to assess child development in rural African settings. *PLoS Med* 2010;7(5):e1000273. doi: 10.1371/journal.pmed.1000273
30. Bayley N. Bayley Scales of Infant and Toddler Development. San Antonio, TX: The Psychological Corporation 2006.
31. Murray-Kolb LE, Rasmussen ZA, Scharf RJ, et al. The MAL-ED Cohort Study: Methods and Lessons Learned When Assessing Early Child Development and Caregiving Mediators in Infants and Young Children in 8 Low- and Middle-Income Countries. *Clinical Infectious Diseases* 2014;59:S261-S72. doi: 10.1093/cid/ciu437
32. Donowitz JR, Petri WA. Pediatric small intestine bacterial overgrowth in low-income countries. *Trends Mol Med* 2015;21(1):6-15. doi: 10.1016/j.molmed.2014.11.001 [published Online First: 2014/11/15]
33. Donowitz JR, Haque R, Kirkpatrick BD, et al. Small Intestine Bacterial Overgrowth and Environmental Enteropathy in Bangladeshi Children. *MBio* 2016;7(1):e02102-15. doi: 10.1128/mBio.02102-15 [published Online First: 2016/01/12]
34. Trammell SA, Schmidt MS, Weidemann BJ, et al. Nicotinamide riboside is uniquely and orally bioavailable in mice and humans. *Nat Commun* 2016;7:12948. doi: 10.1038/ncomms12948 [published Online First: 2016/10/10]
35. Buchanan AM, Fiorillo SP, Omondi MW, et al. Establishment of biochemistry reference values for healthy Tanzanian infants, children and adolescents in Kilimanjaro Region. *Trop Med Int Health* 2015;20(11):1569-77. doi: 10.1111/tmi.12580 [published Online First: 2015/08/25]
36. Coghlan RF, Oberdorf JA, Sienko S, et al. A degradation fragment of type X collagen is a real-time marker for bone growth velocity. *Science Translational Medicine* 2017
37. Inagaki T, Moschetta A, Lee YK, et al. Regulation of antibacterial defense in the small intestine by the nuclear bile acid receptor. *Proc Natl Acad Sci U S A* 2006;103(10):3920-5. doi: 10.1073/pnas.0509592103 [published Online First: 2006/02/10]
38. Claus SP, Swann JR. Nutrimetabonomics: Applications for Nutritional Sciences, with Specific Reference to Gut Microbial Interactions. In: Doyle MP, Klaenhammer TR, eds. Annual Review of Food Science and Technology, Vol 4. Palo Alto: Annual Reviews 2013:381-99.
39. Ghodsi M, Liu B, Pop M. DNACLUST: accurate and efficient clustering of phylogenetic marker genes. *BMC Bioinformatics* 2011;12:271. doi: 10.1186/1471-2105-12-271

For peer review only - http://bmjopen.bmj.com/site/about/guidelines.xhtml

40. Liu J, Gratz J, Amour C, et al. A laboratory-developed TaqMan Array Card for simultaneous detection of 19 enteropathogens. *J Clin Microbiol* 2013;51(2):472-80. doi: 10.1128/JCM.02658-12 [published Online First: 2012/11/21]
41. Liu J, Gratz J, Amour C, et al. Optimization of Quantitative PCR Methods for Enteropathogen Detection. *PLoS One* 2016;11(6):e0158199. doi: 10.1371/journal.pone.0158199 [published Online First: 2016/06/23]
42. Liu J, Platts-Mills JA, Juma J, et al. Use of quantitative molecular diagnostic methods to identify causes of diarrhoea in children: a reanalysis of the GEMS case-control study. *Lancet* 2016;388(10051):1291-301. doi: 10.1016/S0140-6736(16)31529-X
43. Clinical_and_Laboratory_Standards_Institute. Performance Standards for Antimicrobial Susceptibility Testing, 27 ed. Wayne, Pennsylvania: Clinical and Laboratory Standards Institute 2017.
44. Ikehara Y, Nishihara S, Yasutomi H, et al. Polymorphisms of two fucosyltransferase genes (Lewis and Secretor genes) involving type I Lewis antigens are associated with the presence of anti-Helicobacter pylori IgG antibody. *Cancer Epidemiol Biomarkers Prev* 2001;10(9):971-7.
45. McGovern DP, Jones MR, Taylor KD, et al. Fucosyltransferase 2 (FUT2) non-secretor status is associated with Crohn's disease. *Hum Mol Genet* 2010;19(17):3468-76. doi: 10.1093/hmg/ddq248
46. Marionneau S, Airaud F, Bovin NV, et al. Influence of the combined ABO, FUT2, and FUT3 polymorphism on susceptibility to Norwalk virus attachment. *J Infect Dis* 2005;192(6):1071-7. doi: 10.1086/432546 [published Online First: 2005/08/09]
47. Yusuf S, Lonn E, Pais P, et al. Blood-Pressure and Cholesterol Lowering in Persons without Cardiovascular Disease. *N Engl J Med* 2016;374(21):2032-43. doi: 10.1056/NEJMoa1600177
48. Locks LM, Manji KP, McDonald CM, et al. Effect of zinc and multivitamin supplementation on the growth of Tanzanian children aged 6-84 wk: a randomized, placebo-controlled, double-blind trial. *Am J Clin Nutr* 2016;103(3):910-8. doi: 10.3945/ajcn.115.120055
49. Soofi S, Cousens S, Iqbal SP, et al. Effect of provision of daily zinc and iron with several micronutrients on growth and morbidity among young children in Pakistan: a cluster-randomised trial. *Lancet* 2013;382(9886):29-40. doi: 10.1016/S0140-6736(13)60437-7

For peer review only

For peer review only - http://bmjopen.bmj.com/site/about/guidelines.xhtml

FIGURE HEADINGS

Figure 1: The Tryptophan-kynurenine-niacin pathway in the intestinal mucosa. Dietary tryptophan is metabolized to kynurenine by indoleamine 2,3-dioxygenase (IDO)—whose activity is increased in settings of inflammation. Adverse effects of elevated IDO activity relate to modulation of the immune response, decreased activity of mTOR in producing antimicrobial peptides, and decreased tryptophan for protein synthesis. Intervention with nicotinamide ameliorates some of these effects in preclinical models; in the current study, we hypothesize that intervention with nicotinamide will increase linear growth via normalization of the tryptophan-kynurenine-niacin pathway and restored enterocyte activity. Both nicotinic acid and nicotinamide are considered dietary forms of niacin. Abbreviations: IDO = indoleamine 2,3-dioxygenase; NAD = nicotinamide adenine dinucleotide.

Figure 2: Taqman Array Card (TAC) schematic. Burden of intestinal pathogens will be assessed in stool samples from participants at multiple time points using TAC.

For peer review only

For peer review only - http://bmjopen.bmj.com/site/about/guidelines.xhtml

Table 1: Timing of interventions and measurements.

	Preg.	Birth-2 wks	Month of life													
			1	2	3	4	5	6	7	8	9	10	11	12	15	18
RECRUITMENT, CONSENT	X	X														
ENROLLMENT		X														
INTERVENTIONS																
A. Nicotinamide intervention																
Nicotinamide (or placebo) to mother: daily 0-6 mo			X	X	X	X	X	X								
Nicotinamide (or placebo) to child: daily 6-12 mo									X	X	X	X	X	X	X	X
B. Antimicrobial intervention																
Azithromycin single dose to child								X			X			X	X	
Nitazoxanide 3-day course to child														X	X	
MEASUREMENTS																
A. Mother																
Weight: Month 11													X			
Height: Month 11													X			
Blood								X								
Stool								X								
Saliva (FUT2 secretor status)									X					X		
B. Child																
1. Questionnaire																
Education, environment, socioeconomic			X													
Illness, treatment, breast feeding, food security			X	X	X	X	X	X	X	X	X	X	X	X	X	X
2. Breast milk: Months 1 and 5																
Tryptophn, niacin, nicotinamide, vitamers			X				X									
Vitamins			X				X									
Protein			X				X									
2. Blood																
Safety assessment (CMP, CBC in subset)				X						X						
Hemoglobin														X		X
IGF-1														X		X
CRP														X		X
Collagen X														X		X
Tryptophan metabolites														X		X
Metabolomics														X		X
3. Stool																
Microbiome								X[a]						X[b]		X
Taqman Array Cards								X[a]						X		X
Myeloperoxidase								X						X		X
4. Urine																
Metabolomics								X						X		X
5. Saliva																

For peer review only - http://bmjopen.bmj.com/site/about/guidelines.xhtml

FUT2 secretor status								X								
6. Breath																
H_2, methane (small intestinal bacterial overgrowth)								X						X		X
7. Developmental testing																
Observation of Maternal Child Interaction								X								
Malawi Developmental Assessment Tool																X
Bayley Scales of Development																X

For peer review only

For peer review only - http://bmjopen.bmj.com/site/about/guidelines.xhtml

Table 2: Primary and secondary outcome measures by study intervention.

Outcome	Timing (age of child in months)	Antimicrobial intervention		Nicotinamide intervention
Primary outcome				
LAZ	18	X		X
Secondary outcomes				
• Proportion of stunting (LAZ<-2)	18	X		X
• Weight-for-age (WAZ) and weight-for-length (WLZ) z-scores	18	X		X
• Metabolomic assay of gut microbial metabolism and alterations to tryptophan-niacin and other pathways	6, 12, 18	X		X
• Tryptophan:kynurenine ratio and other pathway metabolites in serum	12, 18	X		X
• IGF-1 at 12 and 18 months	12, 18	X		X
• High-sensitivity C-reactive protein at 12 and 18 months	12, 18	X		X
• Stool myeloperoxidase at months 6,12 and 18 months	6, 12, 18	X		X
• Prevalence of enteric pathogens	6, 6.5, 12, 12.5,18	X		X
• Change in microbiota (by traditional sequencing) (subset of participants)	6, 6.5, 12, 18	X		X
• Change in small intestinal bacterial overgrowth as assessed by exhaled increased hydrogen following ingestion of sugar (subset of participants)	6, 12, 18	X		X
• Difference in breast milk composition (nicotinamide, tryptophan, amino acids)	1, 5			X
• Symptomatic diarrhea		X		X
• Hospitalization and all-cause mortality	18	X		X
• Cognitive outcomes Malawi Development Assessment Tool (MDAT) assessment and the Bayley Scales of Infant and Toddler Development	18	X		X

For peer review only

For peer review only - http://bmjopen.bmj.com/site/about/guidelines.xhtml

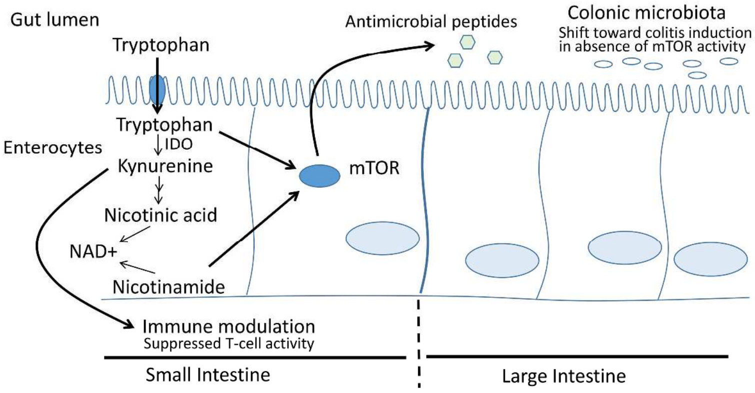

Figure 1: The Tryptophan-kynurenine-niacin pathway in the intestinal mucosa. Dietary tryptophan is metabolized to kynurenine by indoleamine 2,3-dioxygenase (IDO)—whose activity is increased in settings of inflammation. Adverse effects of elevated IDO activity relate to modulation of the immune response, decreased activity of mTOR in producing antimicrobial peptides, and decreased tryptophan for protein synthesis. Intervention with nicotinamide ameliorates some of these effects in preclinical models; in the current study, we hypothesize that intervention with nicotinamide will increase linear growth via normalization of the tryptophan-kynurenine-niacin pathway and restored enterocyte activity. Both nicotinic acid and nicotinamide are considered dietary forms of niacin. Abbreviations: IDO = indoleamine 2,3-dioxygenase; NAD = nicotinamide adenine dinucleotide.

338x190mm (300 x 300 DPI)

For peer review only - http://bmjopen.bmj.com/site/about/guidelines.xhtml

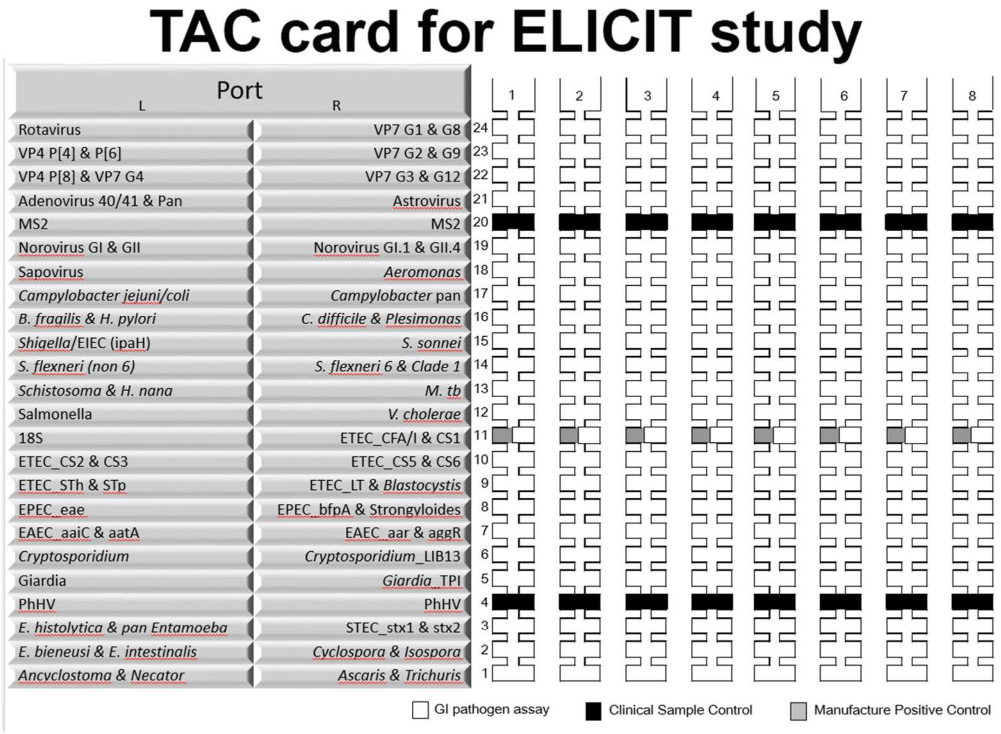

Figure 2: Taqman Array Card (TAC) schematic. Burden of intestinal pathogens will be assessed in stool samples from participants at multiple time points using TAC.

97x71mm (300 x 300 DPI)

w only

SPIRIT
STANDARD PROTOCOL ITEMS: RECOMMENDATIONS FOR INTERVENTIONAL TRIALS

SPIRIT 2013 Checklist: Recommended items to address in a clinical trial protocol and related documents*

Section/item	Item No	Description	Addressed on page number
**Administrative information**			
Title	1	Descriptive title identifying the study design, population, interventions, and, if applicable, trial acronym	________1______
Trial registration	2a	Trial identifier and registry name. If not yet registered, name of intended registry	________2______
	2b	All items from the World Health Organization Trial Registration Data Set	______________
Protocol version	3	Date and version identifier	________3______
Funding	4	Sources and types of financial, material, and other support	________23_____
Roles and responsibilities	5a	Names, affiliations, and roles of protocol contributors	_____22-23_____
	5b	Name and contact information for the trial sponsor	________3______
	5c	Role of study sponsor and funders, if any, in study design; collection, management, analysis, and interpretation of data; writing of the report; and the decision to submit the report for publication, including whether they will have ultimate authority over any of these activities	_______23______
	5d	Composition, roles, and responsibilities of the coordinating centre, steering committee, endpoint adjudication committee, data management team, and other individuals or groups overseeing the trial, if applicable (see Item 21a for data monitoring committee)	_______NA______

For peer review only - http://bmjopen.bmj.com/site/about/guidelines.xhtml

For peer review only

**Introduction**			
Background and rationale	6a	Description of research question and justification for undertaking the trial, including summary of relevant studies (published and unpublished) examining benefits and harms for each intervention	______5-7_____
	6b	Explanation for choice of comparators	______7_______
Objectives	7	Specific objectives or hypotheses	______7-8_____
Trial design	8	Description of trial design including type of trial (eg, parallel group, crossover, factorial, single group), allocation ratio, and framework (eg, superiority, equivalence, noninferiority, exploratory)	______7-8_____
**Methods: Participants, interventions, and outcomes**			
Study setting	9	Description of study settings (eg, community clinic, academic hospital) and list of countries where data will be collected. Reference to where list of study sites can be obtained	______9_______
Eligibility criteria	10	Inclusion and exclusion criteria for participants. If applicable, eligibility criteria for study centres and individuals who will perform the interventions (eg, surgeons, psychotherapists)	______8_______
Interventions	11a	Interventions for each group with sufficient detail to allow replication, including how and when they will be administered	______9-10____
	11b	Criteria for discontinuing or modifying allocated interventions for a given trial participant (eg, drug dose change in response to harms, participant request, or improving/worsening disease)	_____19-20____
	11c	Strategies to improve adherence to intervention protocols, and any procedures for monitoring adherence (eg, drug tablet return, laboratory tests)	______10______
	11d	Relevant concomitant care and interventions that are permitted or prohibited during the trial	______10_______
Outcomes	12	Primary, secondary, and other outcomes, including the specific measurement variable (eg, systolic blood pressure), analysis metric (eg, change from baseline, final value, time to event), method of aggregation (eg, median, proportion), and time point for each outcome. Explanation of the clinical relevance of chosen efficacy and harm outcomes is strongly recommended	_18-19, Table 1__
Participant timeline	13	Time schedule of enrolment, interventions (including any run-ins and washouts), assessments, and visits for participants. A schematic diagram is highly recommended (see Figure)	_____9-10______

For peer review only - http://bmjopen.bmj.com/site/about/guidelines.xhtml

Sample size	14	Estimated number of participants needed to achieve study objectives and how it was determined, including clinical and statistical assumptions supporting any sample size calculations	______20_______
Recruitment	15	Strategies for achieving adequate participant enrolment to reach target sample size	______21_______

Methods: Assignment of interventions (for controlled trials)

Allocation:			
Sequence generation	16a	Method of generating the allocation sequence (eg, computer-generated random numbers), and list of any factors for stratification. To reduce predictability of a random sequence, details of any planned restriction (eg, blocking) should be provided in a separate document that is unavailable to those who enrol participants or assign interventions	______9_______
Allocation concealment mechanism	16b	Mechanism of implementing the allocation sequence (eg, central telephone; sequentially numbered, opaque, sealed envelopes), describing any steps to conceal the sequence until interventions are assigned	______9_______
Implementation	16c	Who will generate the allocation sequence, who will enrol participants, and who will assign participants to interventions	______9_______
Blinding (masking)	17a	Who will be blinded after assignment to interventions (eg, trial participants, care providers, outcome assessors, data analysts), and how	______9-10_____
	17b	If blinded, circumstances under which unblinding is permissible, and procedure for revealing a participant's allocated intervention during the trial	______19-20____

Methods: Data collection, management, and analysis

Data collection methods	18a	Plans for assessment and collection of outcome, baseline, and other trial data, including any related processes to promote data quality (eg, duplicate measurements, training of assessors) and a description of study instruments (eg, questionnaires, laboratory tests) along with their reliability and validity, if known. Reference to where data collection forms can be found, if not in the protocol	____10-18______
	18b	Plans to promote participant retention and complete follow-up, including list of any outcome data to be collected for participants who discontinue or deviate from intervention protocols	_____9-10______

For peer review only - http://bmjopen.bmj.com/site/about/guidelines.xhtml

Data management	19	Plans for data entry, coding, security, and storage, including any related processes to promote data quality (eg, double data entry; range checks for data values). Reference to where details of data management procedures can be found, if not in the protocol	_____18________
Statistical methods	20a	Statistical methods for analysing primary and secondary outcomes. Reference to where other details of the statistical analysis plan can be found, if not in the protocol	_____18-19_____
	20b	Methods for any additional analyses (eg, subgroup and adjusted analyses)	_____19_______
	20c	Definition of analysis population relating to protocol non-adherence (eg, as randomised analysis), and any statistical methods to handle missing data (eg, multiple imputation)	_____19_______
**Methods: Monitoring**			
Data monitoring	21a	Composition of data monitoring committee (DMC); summary of its role and reporting structure; statement of whether it is independent from the sponsor and competing interests; and reference to where further details about its charter can be found, if not in the protocol. Alternatively, an explanation of why a DMC is not needed	_____19-20_____
	21b	Description of any interim analyses and stopping guidelines, including who will have access to these interim results and make the final decision to terminate the trial	_____19-20_____
Harms	22	Plans for collecting, assessing, reporting, and managing solicited and spontaneously reported adverse events and other unintended effects of trial interventions or trial conduct	_____19-20_____
Auditing	23	Frequency and procedures for auditing trial conduct, if any, and whether the process will be independent from investigators and the sponsor	______18_______
**Ethics and dissemination**			
Research ethics approval	24	Plans for seeking research ethics committee/institutional review board (REC/IRB) approval	______22_______
Protocol amendments	25	Plans for communicating important protocol modifications (eg, changes to eligibility criteria, outcomes, analyses) to relevant parties (eg, investigators, REC/IRBs, trial participants, trial registries, journals, regulators)	______22_______

For peer review only - http://bmjopen.bmj.com/site/about/guidelines.xhtml

Consent or assent	26a	Who will obtain informed consent or assent from potential trial participants or authorised surrogates, and how (see Item 32)	_____9________
	26b	Additional consent provisions for collection and use of participant data and biological specimens in ancillary studies, if applicable	_____9________
Confidentiality	27	How personal information about potential and enrolled participants will be collected, shared, and maintained in order to protect confidentiality before, during, and after the trial	_____18________
Declaration of interests	28	Financial and other competing interests for principal investigators for the overall trial and each study site	_____23________
Access to data	29	Statement of who will have access to the final trial dataset, and disclosure of contractual agreements that limit such access for investigators	_____18________
Ancillary and post-trial care	30	Provisions, if any, for ancillary and post-trial care, and for compensation to those who suffer harm from trial participation	_____19-20_____
Dissemination policy	31a	Plans for investigators and sponsor to communicate trial results to participants, healthcare professionals, the public, and other relevant groups (eg, via publication, reporting in results databases, or other data sharing arrangements), including any publication restrictions	________22______
	31b	Authorship eligibility guidelines and any intended use of professional writers	________22______
	31c	Plans, if any, for granting public access to the full protocol, participant-level dataset, and statistical code	________22______
**Appendices**			
Informed consent materials	32	Model consent form and other related documentation given to participants and authorised surrogates	__Supplement__
Biological specimens	33	Plans for collection, laboratory evaluation, and storage of biological specimens for genetic or molecular analysis in the current trial and for future use in ancillary studies, if applicable	____10-18______

*It is strongly recommended that this checklist be read in conjunction with the SPIRIT 2013 Explanation & Elaboration for important clarification on the items. Amendments to the protocol should be tracked and dated. The SPIRIT checklist is copyrighted by the SPIRIT Group under the Creative Commons "Attribution-NonCommercial-NoDerivs 3.0 Unported" license.

For peer review only - http://bmjopen.bmj.com/site/about/guidelines.xhtml

BMJ Open

Early Life Interventions for Childhood Growth and Development in Tanzania (ELICIT): A Protocol for a Randomized Factorial, Double-Blind, Placebo-Controlled Trial of Azithromycin, Nitazoxanide and Nicotinamide

Journal:	*BMJ Open*
Manuscript ID	bmjopen-2018-021817.R2
Article Type:	Protocol
Date Submitted by the Author:	20-Apr-2018

Complete List of Authors:	DeBoer, Mark; University of Virginia, Pediatrics<br>Platts-Mills, James; University of Virginia, Medicine<br>Scharf, Rebecca; University of Virginia Children's Hospital, Pediatrics<br>McDermid, Joann; University of Virginia, Medicine<br>Wanjuhi, Anne; University of Virginia, Global Health<br>Gratz, Jean; University of Virginia, Medicine<br>Svensen, Erling; Haukeland Universitetssjukehus<br>Swann, Jon; Imperial College London<br>Donowitz, Jeffrey; University of Virginia, Medicine<br>Jatosh, Samwel; Haydom Lutheran Hospital, Haydom Global Health Research Center<br>Houpt, Eric; University of Virginia,<br>Mduma, Estomih ; Haydom Lutheran Hospital

**Primary Subject Heading**:	Global health
Secondary Subject Heading:	Infectious diseases, Nutrition and metabolism, Paediatrics, Public health
Keywords:	growth, stunting, development, antibiotics, niacin, Tanzania

SCHOLARONE™ Manuscripts

Early Life Interventions for Childhood Growth and Development in Tanzania (ELICIT): A Protocol for a Randomized Factorial, Double-Blind, Placebo-Controlled Trial of Azithromycin, Nitazoxanide and Nicotinamide

Mark D. DeBoer MD, MSc, MCR[1,7]; James A. Platts-Mills MD[2]; Rebecca J. Scharf MD, MPH[1,2]; Joann M. McDermid Ph.D, RD[2]; Anne Wanjuhi MPH[1]; Jean Gratz MS[2]; Erling Svensen Ph.D[3]; Jon R. Swann Ph.D[4]; Jeffrey R. Donowitz MD[5]; Samwel Jatosh BS[6]; Eric R. Houpt MD[2]; Estomih Mduma MPH[6]

1. Department of Pediatrics, University of Virginia, Charlottesville, VA, USA;
2. Center for Global Health, Department of Medicine, University of Virginia, Charlottesville, VA, USA;
3. Department of Global Health and Primary Care, University of Bergen, Bergen, Norway;
4. Imperial College of London, London, UK;
5. Division of Infectious Disease, Children's Hospital of Richmond at Virginia Commonwealth University
6. Haydom Global Health Research Centre, Haydom, Tanzania;
7. Corresponding Author:

Division of Pediatric Endocrinology
University of Virginia School of Medicine
P.O. Box 800386
Charlottesville, VA 22908
Phone: 434-924-5956
Fax: 434-924-9181
Email: deboer@virginia.edu

For peer review only - http://bmjopen.bmj.com/site/about/guidelines.xhtml

ABSTRACT

Introduction: In many developing areas in the world, a high burden of enteric pathogens in early childhood are associated with growth deficits. The tryptophan-kynurenine-niacin pathway has been linked to enteric inflammatory responses to intestinal infections. However, it is not known in these settings whether scheduled antimicrobial intervention to reduce subclinical enteric pathogen carriage or repletion of the tryptophan-kynurenine-niacin pathway improves linear growth and development.

Methods and analysis: We are conducting a randomized, placebo-controlled, factorial intervention trial in the rural setting of Haydom, Tanzania. We are recruiting 1188 children within the first 14 days of life, who will be randomized in a 2x2 factorial design to administration of antimicrobials (azithromycin and nitazoxanide, randomized together) and nicotinamide. The nicotinamide is administered as a daily oral dose, which for breast-feeding children aged 0-6 months is given to the mother and for children aged 6-18 months is given to the child directly. Azithromycin is given to the child as a single oral dose at months 6, 9, 12, and 15; nitazoxanide is given as a 3-day course at months 12 and 15. Mother/child pairs are followed via monthly in-home visits. The primary outcome is the child's length-for-age Z-score at 18 months. Secondary outcomes for the child include additional anthropometry measures; stool pathogen burden and bacterial microbiome; systemic and enteric inflammation; blood metabolomics, growth factors, inflammation and nutrition; hydrogen breath assessment to estimate small-intestinal bacterial overgrowth; and assessment of cognitive development. Secondary outcomes for the mother include breastmilk content of nicotinamide, other vitamins and amino acids; blood measures of tryptophan-kynurenine-niacin pathway; and stool pathogens.

Ethics and dissemination: This trial has been approved by the Tanzanian National Institute for Medical Research, the Tanzanian FDA and the University of Virginia IRB. Findings will be presented at national and international conferences and published in peer-review journals.

Trial registration number: ClinicalTrials.gov #NCT03268902.

For peer review only - http://bmjopen.bmj.com/site/about/guidelines.xhtml

For peer review only

Key words: growth, stunting, development, antibiotics, niacin, Tanzania

Protocol version: 5.0, 4 December 2017

Protocol Sponsor: Haydom Lutheran Hospital, Haydom, Manyara, Tanzania

For peer review only

For peer review only - http://bmjopen.bmj.com/site/about/guidelines.xhtml

STRENGTHS AND LIMITATIONS OF THIS STUDY

- This study uses a factorial design to determine independent contributions of two different intervention approaches onearly childhood growth and development.
- The study is set in a rural site in sub-Saharan Africa with a high degree of both enteric pathogen carriage and stunting.
- This study assesses intervention with nicotinamide, targeting the tryptophan-kynurenine-niacin pathway, which may have important roles in intestinal bacterial content and immune and metabolic response.
- In addition to the primary outcome of length-for-age Z-score, this study measures extensive secondary outcomes related to infection, nutrition, metabolism and cognitive development.
- Due to sample size constraints, one limitation of the study is that two different antimicrobial interventions (azithromycin and nitazoxanide) are randomized together, limiting the ability to determine any individual contribution of either of these to study outcomes.

For peer review only

INTRODUCTION

Childhood growth and thriving remain sub-optimal in low-resource settings worldwide, underscoring the need to better understand underlying etiologies and to identify interventions toward improving outcomes.[1 2] The Etiology, Risk Factors, and Interactions of Enteric Infections and Malnutrition and the Consequences for Child Health (MAL-ED) study evaluated correlates of childhood growth and cognitive development in 8 low- to middle-income countries (LMIC) worldwide, including the region around Haydom, Tanzania.[3 4] Haydom is in a rural, semi-arid area with a high degree of poverty. Children in Haydom followed in MAL-ED had a prevalence of stunting of 70% at 18 months of age.[5] Data from MAL-ED also showed that linear growth deficits correlated with lower scores for cognitive development,[6] potentially through shared barriers to optimal growth and brain development.[1 7]

Antimicrobials to improve childhood growth

In MAL-ED, children in the Haydom site had a high burden of infection with intestinal pathogens starting early in life. High carriage of enteropathogens, even in the absence of diarrhea, was associated with a 1.36 increased odds of having lower length for age.[3] As for the specific enteropathogens, among the most prevalent pathogens at age 6 months were enteroaggregative *E.coli* (48%) and *Campylobacter* (27%),[8] each of which were associated with a 0.85 cm and 0.83 cm, respectively, shortfall in height attainment.[9] Both of these infections are treatable with azithromycin. Other highly prevalent pathogens by age 6 months in the MAL-ED study included *Giardia* (22%) and *Cryptosporidium* (6%), and early *Giardia* infection was also associated with growth shortfalls.[10] Both of these protozoal infections are treatable with nitazoxanide. These observations raise questions as to whether such antibiotics may be useful in ameliorating growth deficits.

A meta-analysis of 10 randomized controlled trials showed that antibiotic use increased height by 0.04 cm/month.[11] Another study demonstrated that randomized administration of

For peer review only - http://bmjopen.bmj.com/site/about/guidelines.xhtml

azithromycin to asymptomatic young children in Ethiopia was associated with a halving of mortality,[12] suggesting overall effects of suppressing bacterial pathogens in areas with high rates of infectious disease. However results from individual studies of azithromycin mass drug-administration on growth have not shown consistent results.[13] Among children with cryptosporidial infection in Zambia, treatment with nitazoxanide resulted in reduced mortality, while linear growth was not assessed.[14] Therefore, the role of these antimicrobials for linear growth remains unclear.

Nicotinamide to improve childhood growth

Another potential means of improving growth is by targeting the tryptophan-kynurenine-niacin pathway (Figure 1). In this pathway, dietary tryptophan is absorbed and then metabolized by indoleamine 2,3-dioxygenase (IDO) to kynurenine and later to nicotinic acid, a key precursor to NAD+. The importance of this pathway has been supported by recent work identifying mutations in the NAD+ pathway as a cause of birth defects in humans; in animal models with these mutations, birth defects were prevented via supplementation with niacin.[15] Deficiencies in this pathway have been noted in areas where diets are heavily maize-based, leading to pellagra. Infants at the Haydom site in MAL-ED were noted to have inadequate dietary intake of multiple vitamins[16]; while deficiency in niacin was not assessed, individuals in the region around Haydom eat a diet based heavily on maize, with potentially low bioavailability of tryptophan. Basic science models have demonstrated that tryptophan deficiency is associated with reduced growth velocity.[17]

Abnormalities in the tryptophan-kynurenine-niacin pathway are also associated with intestinal inflammation, including during response to intestinal pathogens.[18 19] In animal models, an inability to transport tryptophan into enterocytes results in diarrhea, while provision of nicotinamide (a form of niacin) partly restores function of mTOR in enterocytes, increasing secretion of antimicrobial peptides and reducing diarrhea,[18 19] suggesting effects on diarrheal

For peer review only - http://bmjopen.bmj.com/site/about/guidelines.xhtml

pathogens. IDO activity is increased during inflammation, including in inflammatory bowel disease[20 21] and during infections;[22] this appears to be a maladaptive in most cases, as kynurenine has potential immunosuppressive effects.[23 24] In MAL-ED, children at the Haydom site with higher ratios of kynurenine:tryptophan were noted to have poorer linear growth.[24] Given that 1) children in the Haydom area have a maize-based diet low in tryptophan, a key precursor to niacin/nicotinamide and NAD+, 2) preclinical studies demonstrate improvements in antimicrobial peptides and reduced diarrhea with nicotinamide supplementation, 3) an increase in nicotinamide as a precursor of NAD+ should cause reduced need for the tryptophan-kynurenine-niacin pathway, reducing activity of IDO and thereby reducing kynurenine, and 4) higher kynurenine:tryptophan is associated with poor growth, we reasoned that intervention with nicotinamide among children in this region would improve linear growth. However, because growth is a long-term process and nicotinamide is a water-soluble vitamin without a mechanism for on-going storage, we reasoned that an intervention with nicotinamide would need to be given over a long period of time.

Study hypotheses

We hypothesized that among children with a high degree of stunting and enteric pathogen burden in this region, intervention with 1) antimicrobials and/or 2) nicotinamide would increase linear growth. We further set out to interrogate the potential mechanisms behind these effects. In the case of antimicrobial intervention, we hypothesize that intervention with azithromycin and nitazoxanide (relative to placebo) would result in a decrease in enteropathogen burden that would be associated with an increase in growth. In the case of nicotinamide (relative to placebo), we hypothesize that intervention will result in a reduction in the kynurenine:tryptophan ratio, an increase in availability of nicotinamide for NAD+ synthesis and associated increases in growth.

For peer review only - http://bmjopen.bmj.com/site/about/guidelines.xhtml

OBJECTIVE

The primary objectives of this randomized factorial, double-blind, placebo-controlled trial are to determine if interventions with 1) antimicrobials and 2) nicotinamide are associated with increased length-for-age z-score (LAZ, relative to placebo-treated children) by age 18 months. Specifically, the antimicrobial intervention consists of azithromycin administered as a single dose at age 6, 9, 12, and 15 months and nitazoxanide as a 3-day course at age 12 and 15 months (with both antimicrobials randomized together). The nicotinamide intervention consists of nicotinamide 250 mg daily administered daily by mouth to the child's mother during breast feeding 0-6 months (and thus passed on to the child via breast milk[25]) and then 100 mg administered daily by mouth to the child during months 6-18. These interventions are delivered in a factorial manner such that children receive both interventions, either intervention alone or neither intervention.

Because multiple prior large-scale studies of other preparations of vitamins and antibiotics have failed to show effects on linear growth, we have multiple secondary outcomes to interrogate additional physiological effects of the interventions—potentially helping to identify underlying mechanisms. These secondary outcomes include measures of inflammation, intestinal bacterial content (pathogens and otherwise), and measures of metabolism, development and health.

SETTING

Haydom is a rural area in north-central Tanzania, approximately 300 km west of Arusha, the nearest urban center.[4] There is poor infrastructure for roads and other resources, and the local population of approximately 20,000 depends primarily on subsistence agriculture. Haydom Lutheran Hospital (HLH) is the central hub for the area and has a strong relationship with the Hadyom community. Healthcare in the area relies both on HLH and reproductive child health system (RCHS) with 38 outreach clinics across 74 villages, where children are followed monthly

For peer review only - http://bmjopen.bmj.com/site/about/guidelines.xhtml

for weight-checks and immunizations. Given the low HIV seroprevalence (<2%) among mothers of childbearing age, HIV testing is not performed I the current study. Because of its elevation (1,700 meters), Haydom is not a high-risk area for malaria, with almost no *Plasmodium falciparum* in the region. Among the families followed in MAL-ED, 92.3% lived in houses with mud floors, 0% had electricity, and 67.9% obtained their water from an unprotected well or surface water.[4] Further estimates of the low socioeconomic status of the area have been published previously.[4]

METHODS AND ANALYSIS (Standard Protocol Items for Randomized Trials)

Eligibility

Participants are eligible if the mother is ≥18 years and the child is age ≤14 days and was born in the Haydom catchment area (defined for study purposes as 25 km radius of Haydom Lutheran Hospital).

Exclusion criteria are as follows: maternal inability to adhere to the protocol, multiple gestation, significant birth defect or neonatal illness, weight <1,500 g at enrollment (age ≤ 14 days), lack of intent to breastfeed infant and plan to move from area within the next 18 months.

Recruitment

Recruitment occurs via survey by field teams of surrounding villages and via input from local community healthcare workers. Pregnant women are identified and informed regarding the study. Pregnant women in the third trimester may provide informed consent for the study but are not enrolled until the initial assessment visit of the infant to determine eligibility (which includes ascertainment of infant weight). If informed consent is not obtained during pregnancy, it is obtained at enrollment. The general timeline of study procedures is shown in Table 1.

For peer review only - http://bmjopen.bmj.com/site/about/guidelines.xhtml

Enrollment

Infant/mother pairs are enrolled during the initial study visit to their homes between birth and day of life 14, after eligibility criteria are confirmed, including measurement of infant weight which should be 1.5 or above. Informed consent is provided at this time, if it was not obtained during pregnancy. Eligibility (besides current weight) is confirmed and the mother/child pair is randomized in the study.

Randomization

Randomization occurs at the time of enrollment. Block randomization was used in permuted blocks of eight for the four intervention combinations (antimicrobial + nicotinamide, antimicrobial alone, nicotinamide alone, all placebo). Study teams are assigned permuted blocks before initial study visits to sequentially allocate randomization status to participants based on study identification number assigned prior to the initial study visit.

Intervention

As a factorial study design, the two intervention domains (antimicrobial and nicotinamide) are randomized separately. The antimicrobial intervention consists of two medications that are randomized together: azithromycin and nitazoxanide (vs. placebos of both). Azithromycin 20 mg/kg (vs. placebo) (both manufactured by Universal Corp, Kenya) is administered by study personnel at months 6, 9, 12 and 15 as a single dose of azithromycin (200 mg/5 mL suspension by mouth). This timing was selected (versus earlier initiation) because while children have enteropathogen carriage before 6 month in this area,[8] carriage of azithromycin-susceptible pathogens such as Campylobacter are not highly prevalent, and the decline in LAZ is steeper in the 6-18-month window compared to the 0-6 month window.[5] The nitazoxanide (vs. placebo) (both manufactured by Romark, Florida, USA) is administered as a course of nitazoxanide (100 mg/5 mL) 100 mg twice daily for three days. This timeline was

For peer review only - http://bmjopen.bmj.com/site/about/guidelines.xhtml

selected because nitazoxanide is not approved for use below 12 months. The first of these nitazoxanide doses is administered by the study personnel and the remaining doses are administered at home by the child's family. Families are asked at a subsequent visit about whether they completed the course of nitazoxanide.

The nicotinamide (vs. placebo) (both manufactured by Vita-gen, New York, USA) is delivered as a daily dose throughout the study. During the first 6 months of life, this is delivered to the child via breastmilk. This is performed because of the lack of availability of a liquid preparation of nicotinamide and to emphasize the importance of exclusive breast feeding (i.e., that intake beyond breast milk is not needed during the first 6 months of life). Mothers take nicotinamide 250 mg tablets, one tablet by mouth daily. Prior studies have demonstrated that nicotinamide is present in breast milk in quantities that are associated with serum levels.[25] Haydom-specific data from MAL-ED demonstrated a high prevalence (96%) of breast feeding until children were 6 months old.[5] Because the prevalence of exclusive breastfeeding was low in the area around Haydom during MAL-ED, the current study includes a breast-feeding encouragement and support initiative, utilizing nurses who have received specialized training in breast feeding; these nurse work with the local community healthcare workers in home visits to participating families in the first 6 months of the study. Their message is that exclusive breast feeding is better for mother and baby (highlighting a variety of reasons) and that if the family chooses not to breast feed, that providing breast milk before any complimentary foods will allow the child to receive a greater amount of the needed nutrition—including a greater amount of the nicotinamide ingested by the mother. The lack of intent to breast feed is an exclusion criterion at the time of enrollment; however, if the mother dies or discontinues breast feeding, the child subsequently would not receive nicotinamide/placebo until age 6 months.

From months 6-18 the child receives sachets of nicotinamide 100 mg by mouth daily. Mothers are instructed to mix the nicotinamide powder with a small volume of age-appropriate food (e.g. infant cereal) to give to the child.

For peer review only - http://bmjopen.bmj.com/site/about/guidelines.xhtml

For both the maternal nicotinamide pills and the children's nicotinamide sachets, doses are given as a 60-day supply every two months. When a new supply is provided, the family is asked for the remainder of doses not consumed the prior 60 days. These unused doses are counted and recorded by pharmacy staff to determine adherence.

Data collection

Questionnaires

Study staff use standardized questionnaires designed for the study, in many cases adapted from questionnaires used in the MAL-ED study. These include questions asked at each monthly visit regarding breastfeeding, use of complementary foods, childhood illness and treatment (including whether an antibiotic was used and if so which one), types of food sources given, and food insecurity. Additional questionnaires are asked at other specific time periods, including aspects of the child's home environment (completed at the first month's visit) and the vaccines that the child has received (completed at the 12-month visit).

Anthropometry measures

All field team members are trained in the accurate measurement of anthropometry indices. The child's weight is measured at the initial visit and at each monthly visit through age 18 months. All anthropometry measures for a given month (including the final visit) are able to be performed up to 14 days before or after the target date (which is based on the number of months after the child's birthday. The child's length and head circumference are measured at the initial visit and every three visits thereafter through 18 month. The mother's weight and height are measured at month 10-11. Quarterly, weights are measured using a digital scale that undergoes quality assessments weekly. Infants are weighed on an infant tray with the infant nude or wearing a dry diaper or standing, when able. In between these quarterly weight measures, weights are measured monthly using hanging spring scales that are zeroed before

For peer review only - http://bmjopen.bmj.com/site/about/guidelines.xhtml

each use and assessed for accuracy weekly. Lengths are measured quarterly by two field team members with the child lying flat on a measuring board with a fixed, perpendicular board against which the head is placed and a second adjustable perpendicular board is placed against the feet. Two measurements are obtained; if these are within 2 mm, the average of the two is used; if these are not within 2 mm, a third measurement is obtained and the average of the two closest measurements is used. Head circumference is measured quarterly by placing a non-distensible tape around the child's head just above the eyebrows, above the ears, and around the biggest part of the back of the head. Mid-upper arm circumference is measured quarterly by using the same measuring tape at the mid-point of the upper arm segment. Maternal weight is measured using a standing scale. Maternal height is measured using a portable stadiometer. For quality assurance, 5% of infant weights, lengths, and head circumferences are measured by a supervisor skilled in measurement to compare to the field team's measurement.

Developmental assessments

To assess maternal-child interaction, an important predictor of early childhood development and health,[26 27] The Observation of Maternal Child Interaction (OMCI)[28] assessment is collected at 12 months of age. This observation includes a 5 minute observation of mother and child interacting over a picture book, with ratings given for positive affect, verbal statements, sensitivity, scaffolding, language stimulation, focus and mutual enjoyment, among others.

At 18 months, developmental assessment is performed by trained assessors using two standardized scores, the Malawi Developmental Assessment Tool (MDAT)[29] and the Bayley Scales of Infant and Toddler Development, cognitive assessment (Bayley).[30] The MDAT is a validated score that tests multiple childhood development domains, including gross motor, fine motor, language and social development that has been validated in this age range in Malawi and intended for use in rural sub-Saharan settings. Briefly, this assessment was designed to be

For peer review only - http://bmjopen.bmj.com/site/about/guidelines.xhtml

a culturally-relevant method for examining child development across multiple domains using objects and tools typically available in rural or lower-resource settings. Both direct assessment and caregiver report of skills go into the score.

The Bayley cognitive assessment involves a series of tasks presented to the infant, which assess for play, reasoning, and mental representation. This is a commonly used assessment for early childhood and has been used in many research sites around the world.[6 31]

Specimen collection

Breast milk (months 1 and 5): Participating mothers express a maximal volume of breast milk from one breast (up to 40 mL), using hand expression under low-light conditions. Breast milk will be collected into foil-covered sterile containers and transported on ice to the central laboratory center within 8 hours for archiving of whole milk at -80 degrees.

Blood (months 12 and 18; in a subset months 2 and 8): Blood is drawn from an experienced phlebotomy team using a butterfly needle and syringe and placed in micro-containers for transport to the laboratory for processing into serum and plasma and storage at -80 degrees. In the case of laboratory studies to assess the safety of nicotinamide administration (performed on the first 100 children enrolled), a whole blood specimen is immediately tested in the clinical laboratory at Haydom Lutheran Hospital for complete blood count (CBC).

Stool (months 6, 9, 12, 15, and 18): Stool is collected in diapers that are provided just prior to the stool collection date. Mothers or caregivers are instructed to collect approximately 3–4 spoonfuls of stool and place it within 1 hour into a labeled stool container, seal in a plastic bag, and place inside a transport box with cold packs. The stool collection will be timed prior to a planned study visit, such that the field-worker can collect the sample and the laboratory process the specimen shortly thereafter. Stool samples are then stored at -80 degrees. A subset of

For peer review only - http://bmjopen.bmj.com/site/about/guidelines.xhtml

For peer review only

specimens, prior to freezing will be cultured for E.coli and screened for antibiotic resistance (AMR). This is performed as surveillance for safety and will be performed on a randomly-selected 25 participants per intervention allocation group at 12 and 15 months.

Urine (months 6, 12, and 18): Urine is collected by placing a bag over the child's genitalia at the beginning of a study visit. The child is given adequate hydration, via breast feeding if possible. Collected urine is then transferred in a sterile container that is stored on ice prior to transport to the research site and then stored at -80 degrees.

Saliva (maternal and infant at month 12): Maternal and child saliva is collected into a vial containing storage reagent and stored at -80 degrees for genomic assessment.

Breath (subset of participants at months 6, 9 and 12): Glucose hydrogen breath testing is performed in a subset of children (the last 400 children enrolled) as an assessment of small intestine bacterial overgrowth (SIBO),[32] which has been associated with intestinal inflammation and linear growth shortfall.[33] Children are fasted for 2 hours prior to the initiation of the breath test. They are then given 1 g/kg actual body weight of a glucose dissolved in a 1 g/5ml solution. Following ingestion of the standardized glucose solution, participants have breath collected via the Quintron pediatric breath collection bag system every 20 minutes for 2 hours. This non-invasive procedure involves the child breathing into an anesthesia mask connected to a one-way flutter valve and collection bag. Breath samples are extracted from the collection bag using a syringe and stopcock and placed into specialized vacutainer glass vials. These vials are then transported to our research facility at Haydom Lutheran Hospital for assessment.

Laboratory procedures and storage

For peer review only - http://bmjopen.bmj.com/site/about/guidelines.xhtml

Upon receipt of the samples listed above, the laboratory team at the research site prepares aliquots of samples for storage at -80 degrees for local testing or shipping to additional testing facilities as shown below.

Breast milk

Rationale

During the first 6 months of participation, the only means of the child receiving intervention is through maternal delivery of nicotinamide in breast milk. However, nicotinamide *in vivo* is both derived from tryptophan and converted to multiple other vitamers.[34] As such, we will assess content of nicotinamide, vitamers and tryptophan in breast milk at two time points during the intervention.

Maternal breast milk samples collected at months 1 and 5 will be tested at the United States Department of Agriculture laboratory at the University of California – Davis. Planned LC-MS analyses includes nicotinamide, thiamin, riboflavin, flavin adenine dinucleotide, pyridoxal, pyridoxine, biotin and pantothenic acid concentrations, and on a random subset (n=600 samples) niacin vitamer, and free and total tryptophan concentrations.

Blood

Rationale

Blood testing will include assessment of multiple measures both to verify safety of the interventions and interrogate the effects of the intervention on physiological processes related to linear growth. Safety assessment includes testing of general tests of health with basic metabolic panel, liver function and enzyme testing, and complete blood count. This will assess for differences between intervention groups in measures of kidney, liver and hematopoetic health. Additional tests to interrogate the physiologic effects of the intervention include

For peer review only - http://bmjopen.bmj.com/site/about/guidelines.xhtml

assessment of metabolic effects (using metabolomics) and assessment of levels of factor related to growth (insulin-like growth factor-1 [IGF-1] and collagen X).

Complete blood count (CBC): To assess general safety of the nicotinamide dosing in the first 100 infants at months 2 and 8. CBC is performed at Haydom Lutheran Hospital clinical on a Swelab Alfa hematology analyzer (Boule Diagnositics, Stagna, Sweden; this machine has quality control assessed daily).

Basic metabolic panel (BMP), aminotransferases and bilirubin: To assess general safety of the nicotinamide dosing in the first 100 infants at months 2 and 8, additional laboratory measures will be performed on serum samples at the Kilimanjaro Christian Medical Center. BMP, bilirubin, alanine aminotransferase (ALT) and aspartate aminotransferase (AST) will be assessed using a Cobas Integra 400 Plus biochemistry analyzer (Roche Diagnostics, Germany), according to the manufacturer's instructions.[35]

Insulin-like growth factor-1 (IGF-1) and high-sensitivity C-reactive protein (hsCRP): To interrogate these interventions for changes in growth factor and low-grade systemic inflammation, respectively, IGF-1 and hsCRP will be tested at 12 and 18 months at the UVa Center for Research in Reproduction Ligand Core Laboratory using an Immulite 2000.

Collagen X: We will assess for intervention-associated changes in collagen X (a biomarker of rapid skeletal growth) using an ELISA developed at Shriners Hospital for Children in Portland, OR, and Oregon Health and Science University.[36]

Metabolomics: The tryptophan-kynurenine-niacin pathway will be assessed at 12 and 18 months via metabolomics analyses of serum and plasma using ^{1}H nuclear magnetic resonance

For peer review only - http://bmjopen.bmj.com/site/about/guidelines.xhtml

(NMR) spectroscopy and ultra-performance liquid chromatography-mass spectrometry (UPLC-MS) at Imperial College, London. Assessments include the following: 1) metabolites of the tryptophan-kynurenine-niacin pathway to assess for (among other items) changes in the tryptophan:kynurenine ratio in the setting of nicotinamide treatment;[24] 2) plasma bile salts, which have implications for risk of infection;[37] 3) untargeted metabolomics analysis, providing information on the global metabolic status of the individual including pathways related to the metabolic activity of the gut microbiota and their biochemical interactions with the host;[38] and untargeted UPLC-MS based lipidomics to assess for differences in lipid metabolism by nutritional status.

Stool

Stool testing will include assessment of the following:

Microbiome: Stool bacterial content will be assessed at in a subset of 200 participants at the University of Maryland. Total stool DNA will be isolated by bead beating method. A library will be created each specimen. The libraries will be multiplexed and sequenced by Illumina HiSeq. The resulting reads will be cleaned, binned by specimen ID, separated in operational taxonomic units (OTUs) by DNAclust[39] and compared using metagenomeseq. Metagenomeseq normalizes the data and introduces a correction factor to account for under-sampling of OTUs. The normalized data will be compared using odds ratios and multiple t-tests, with p-values adjusted for multiple testing.

Taqman Array Cards (TAC): Pathogen burden will be assessed using TAC cards (Figure 2) as performed previously.[40 41 42] This will be performed at in the laboratory at Haydom Global Health Research Center using. Briefly, stool samples undergo nucleic acid extraction via the QIAamp Fast Stool DNA Mini kit (Valencia, CA). Nucleic acids are mixed with AgPath-ID One-Step RT-

For peer review only - http://bmjopen.bmj.com/site/about/guidelines.xhtml

PCR buffer (Thermo Fisher) and nuclease free water to a 100ul final volume and run on a custom-designed TAC[40]:

Antibiotic resistance: We will perform stool bacterial culture on a subset of participants to monitor for development of antimicrobial resistance, which is a valid concern of any mass antimicrobial administration. Briefly, stool will be cultured on MacConkey agar and 5 lactose fermenting E. coli colonies pooled and tested for AMR using disc diffusion to screen for ESBL, AmpC, carbapenemase and susceptibility to antibiotics commonly in use locally. Azrythromycin susceptibility will be tested by E-Test.[43]

Myeloperoxidase: As an assessment of stool inflammation, myeloperoxidase will be assessed at months 6, 12 and 18. Testing will be performed at the Global Health Research Center laboratory in Haydom using a commercially-available assay (Alpco, Salem, NH).

Urine

Urine samples at 6, 12 and 18 months will assessed alongside serum and plasma samples at Imperial College, London for untargeted metabolomics analysis by NMR spectroscopy and UPLC-MS, as described above.[38]

Saliva

For a more complete assessment of these interventions on intestinal inflammation in this setting, we will test mothers and children for secretor status of fucosyltransferase 2 (*FUT2*), a regulator of blood group antigens on the intestinal mucosa, as non-secretor status has been associated with infections[44] and other forms of colitis.[45] DNA for mothers and children will be extracted from salivary samples at UVa and *FUT2* genotype will be determined by dot blot assays in a hierarchical method based largely on Lewis Type a and b antigens. Lewis double

For peer review only - http://bmjopen.bmj.com/site/about/guidelines.xhtml

negatives (5-7%) are not able to be characterized as for their secretor status and so will be typed for A, B, and H antigens to allow for phenotypic characterization of all samples.[46]

Breath

To interrogate these interventions for changes in SIBO, a pathophysiological process associated with poor linear growth, we will assess hydrogen and methane quantity of breath samples in a subset of 200 participants. Breath samples collected in glass vials will be extracted and analyzed using a Quintron BreathTracker SC breath chromatograph (Quintron Inc, Milwaukee, WI USA).[33] Data on both hydrogen and methane production will be collected.

Data management and confidentiality

Data are collected onto paper forms, which are then quality checked before being double-entered by two different study team data clerks independently at the research site into a secure web-based system (Multi-Schema Information Capture, MuSIC) managed by UVa Department of Public Health Sciences. Any discrepancies in this double-data entry are reconciled by assessing the original paper form.

Data analysis

Primary endpoint

The primary outcome for the overall study is LAZ at 18 months. For each of the intervention domains we will compare this outcome as a t-test between those randomized to receive the intervention vs. those who did not. This will be performed using a modified intent-to-treat analysis, assessing participants who complete the study and have a measurement of LAZ at 18 months.

Secondary endpoints

For peer review only - http://bmjopen.bmj.com/site/about/guidelines.xhtml

Each of the intervention domains has its own set of secondary outcomes as well (see Table 2), and these will be compared between those who receive the intervention and those who do not. This will initially be performed using the same modified intent-to-treat analysis assessing those with the individual outcome of interest. This will be performed as t-tests for continuous outcomes and chi square tests for binary outcomes.

Secondary analysis for all endpoints (including LAZ at 18 months) will also be performed using per-protocol treatment, defined as participants meeting the following criteria:

- Breastfed through age 6 months
- Received all doses of azithromycin
- Received initial dose of nitazoxanide
- Received at least 50% of nicotinamide doses as measured by pill- and sachet counting.
- Have the outcome of interest measured.

Blinding

The study is being performed in a double-blinded manner. Individual study medications were purchased from the same manufacturer as their corresponding placebo, and placebos were designed to be similar in appearance and taste. Study personnel received the medications and placebos merely labeled as "A" and "B," with the key to this allocation provided to one individual at UVa not otherwise associated with the study. Blinding will be maintained throughout the study unless adverse events occur in significantly disproportionate amounts by intervention allocation status. Allocation status will otherwise only be revealed following completion of collection of data and samples.

Data and safety monitoring

For peer review only - http://bmjopen.bmj.com/site/about/guidelines.xhtml

For peer review only

A data and safety monitoring board has been formed to review data regarding safety of these interventions. This board contains four experts and physicians from Tanzania, the US and the United Kingdom with specialties in gastroenterology, global health, pediatrics, and statistics to assist with reviewing data related to adverse events, health outcomes and surveillance cultures evaluating for antibiotic resistance. Adverse event data are reported monthly by treatment allocation group (in a blinded fashion). The DSMB makes recommendations regarding ongoing safety of the interventions and any need to be un-blinded due to differences in adverse events between treatment allocation group and can recommend discontinuation of any of the intervention arms.

Statistical power

The primary outcome is difference between intervention groups in LAZ at 18 months. The sample size was calculated to provide sufficient power for testing the main effects using separate t-tests for each of the two interventions in this 2 x 2 factorial design, using a standard deviation of 1.03 HAZ at 18 months. If each main effect were tested at the 5% levels, as is customary in the analysis of factorial studies,[47 48] with 270 participants per group, there is 80% power for a difference in LAZ of 0.176. This is within the range of improvement of an increase of 0.16 seen in a prior study after 12 months of micronutrient supplementation,[49] though in Haydom the growth and deficiencies are more severe than most areas, thus the potential for observing a greater effect size. The interaction between main effects of these interventions will then be estimated and tested as a secondary analysis using a 2-way ANOVA. If we were to take a more conservative approach and use a significance level of 2.5% for each of the main effects, with 270 subjects per group, we would still have 80% power to detect an average LAZ increase of 0.193. Finally, if we were to adjust the significance to include the test for the interaction, the F-test for the main effect in a 2-way ANOVA has 80% power with a two-sided significance level of 1.67%, when the main effect of the intervention is to increase the mean LAZ

For peer review only - http://bmjopen.bmj.com/site/about/guidelines.xhtml

by 0.203. All of these are reasonable changes in LAZ to observe for in the Haydom population, given the considerable demonstrated mean deficit in growth. Adjusting for 10% dropout, the total sample size required is 1188. Within our recruitment radius >2507 births occur yearly. We will recruit during a 12-month window. In our extensive research experience at the Haydom MAL-ED site, we observed 0% refusal to participate and a high 81% retention rate by 4 years of follow-up during MAL-ED (and, thus, a study of shorter duration may have improved follow-up). Based on this, we anticipate a high rate of recruitment (>90%) and 18-month retention (>90%). Therefore, accounting for drop-out, we anticipate achieving 1188 mother/child dyads divided between intervention and control, leaving us adequate power to determine differences in LAZ.

Statistics

In addition to use of ITT for the central analysis (t-tests) and the standard 2-way ANOVA to assess main effects and the interaction, we will perform several sensitivity analyses to assess for influences from children who dropped out or died during the study. These will include use of linear mixed-effects models based on repeated measures of anthropometric measures (adjusted for baseline LAZ), assessments of last-measurement carried forward and assumptions about those who dropped out being taller or shorter than the mean. Additional secondary analyses will include assessing hazard ratios for events such as mortality and hospitalization, including time to event analysis.

Study timeline

Enrollment began 5 September 2017 and is planned to continue through 5 September 2018. Children will be followed until age 18 months, resulting in a planned completion in February 2020. Blinding will be maintained until all final study visits are completed. The primary analysis will be performed once data collection is complete for the variables involved.

For peer review only - http://bmjopen.bmj.com/site/about/guidelines.xhtml

To reduce intra-assay variability, the majority of laboratory testing will be batched and performed once complete samples are present.

Potential challenges and limitations

Potential challenges include adequate recruitment of target participant numbers within the one-year study window. Should this be true, we will consider extending the recruitment window. Maintaining adherence of study medication, particularly the daily doses of nicotinamide to breast-feeding mothers during months 0-6, and infants during months 6-18, may be a challenge in a setting in which few individuals are accustomed to taking daily medication or nutritional supplements. To protect against adherence challenges, we performed a pilot study beforehand, identifying potential barriers to adherence and resulting in steps to remind mothers frequently during the first month of the study, with follow-up home visits by the field team and by local community healthcare workers.

ETHICS AND DISSEMINATION

Regulatory authorities

ELICIT has received approval from Tanzania's National Institute of Medical Research (NIMR, reference number HQ/R.8a/Vol.IX/2424, approved 3 March 2017), the University of Virginia Health Sciences Research Institutional Review Board (HSR-IRB, reference #19465, approved 19 January 2017), and the Tanzanian Food and Drug Administration (TFDA, reference number TFDA0017/CTR/0005/02, approved 5 July 2017). All research procedures are conducted in accordance with the Declaration of Helsinki and according to International Conference on Harmonisation Good Clinical Practice guidelines. All participant mothers provide informed consent. An external monitor (FHI360) evaluated the site prior to initiation of the study and performs yearly site visits and assessments thereafter.

For peer review only - http://bmjopen.bmj.com/site/about/guidelines.xhtml

Patient and Public Involvement

Patients and the public were not directly involved in the planning of this study. Results will be communicated to participants via community meetings with local leaders.

Dissemination

This trial has been registered at ClinicalTrials.gov (#NCT03268902). Scientific reports resulting from this study will be made publicly available. Following completion of data accrual, data will be made publicly available in an online database. Data will be presented at major conferences such as those of the American Society of Tropical Medicine and Hygiene, the Pediatric Academic Society, the Endocrine Society, and the African Academy of Sciences. Scientific articles will be published with open-access availability at major scientific journals.

Authors' contributions

MDD, JPM, RJS, JM, JG, ES, EH and EM conceived of this trial and developed this study protocol. EM is the Principal Investigator; MDD is the UVa Principal Investigator. SJ is the Clinical Research Coordinator; AW is the UVa Project Manager. JPM, JG and EH developed procedures related to stool testing for pathogens and antibiotic resistance. JS developed procedures related to metabolomics testing. JRD developed procedures related to testing for small intestinal bacterial overgrowth. JMM developed procedures related to the breastfeeding support of mothers for community, field and nursing staff. RJS and ES developed procedures related to developmental testing. JG oversees all sample processing and laboratory procedures in Haydom. All authors contributed to the development of this manuscript and/or study procedures and to reading and approving the final version for publication.

For peer review only - http://bmjopen.bmj.com/site/about/guidelines.xhtml

Funding statement

This work was supported by the Bill and Melinda Gates Foundation, OPP1141342.

Role of the sponsor and funder

The funder contributed input into the design of the study. Neither the sponsor nor the funder had input into the preparation of this report for publication. The sponsor has ultimate authority into these activities.

Acknowledgments

Romark donated the nitazoxanide.

Competing interests statement

None of the authors or study co-investigators have any competing interests to declare.

REFERENCES

1. Grantham-McGregor S, Cheung YB, Cueto S, et al. Child development in developing countries 1 - Developmental potential in the first 5 years for children in developing countries. *Lancet* 2007;369(9555):60-70.
2. Prendergast AJ, Humphrey JH. The stunting syndrome in developing countries. *Paediatrics and International Child Health* 2014;34(4):250-65. doi: 10.1179/2046905514y.0000000158
3. Kosek MN, Investigators M-EN. Causal Pathways from Enteropathogens to Environmental Enteropathy: Findings from the MAL-ED Birth Cohort Study. *EBioMedicine* 2017;18:109-17. doi: 10.1016/j.ebiom.2017.02.024 [published Online First: 2017/03/08]
4. Mduma ER, Gratz J, Patil C, et al. The etiology, risk factors, and interactions of enteric infections and malnutrition and the consequences for child health and development study (MAL-ED): description of the Tanzanian site. *Clin Infect Dis* 2014;59 Suppl 4:S325-30. doi: 10.1093/cid/ciu439
5. MAL-ED_Network_Investigators. Childhood stunting in relation to the pre- and postnatal environment during the first 2 years of life: The MAL-ED longitudinal birth cohort study. *PLoS Med* 2017;14(10):e1002408. doi: 10.1371/journal.pmed.1002408 [published Online First: 2017/10/25]
6. Scharf RJ, Rogawski ET, Murray-Kolb LE, et al. Early Childhood Growth and Cognitive Outcomes: Findings from the MAL-*ED Study*. *Maternal and Child Nutrition* 2017;in press

For peer review only - http://bmjopen.bmj.com/site/about/guidelines.xhtml

7. Krebs NF, Lozoff B, Georgieff MK. Neurodevelopment: The Impact of Nutrition and Inflammation During Infancy in Low-Resource Settings. *Pediatrics* 2017;139(Suppl 1):S50-S58. doi: 10.1542/peds.2016-2828G
8. Platts-Mills JA, Babji S, Bodhidatta L, et al. Pathogen-specific burdens of community diarrhoea in developing countries: a multisite birth cohort study (MAL-ED). *Lancet Glob Health* 2015;3(9):e564-75. doi: 10.1016/S2214-109X(15)00151-5 [published Online First: 2015/07/19]
9. MAL-ED_Network_Investigators. Relationship between growth and illness, enteropathogens and dietary intakes in the first 2 years of life: findings from the MAL-ED birth cohort study. *BMJ Global Health* 2017;2(4):e000370.
10. Rogawski ET, Bartelt LA, Platts-Mills JA, et al. Determinants and Impact of Giardia Infection in the First 2 Years of Life in the MAL-ED Birth Cohort. *J Pediatric Infect Dis Soc* 2017;6(2):153-60. doi: 10.1093/jpids/piw082
11. Gough EK, Moodie EEM, Prendergast AJ, et al. The impact of antibiotics on growth in children in low and middle income countries: systematic review and meta-analysis of randomised controlled trials. *Bmj-British Medical Journal* 2014;348 doi: 10.1136/bmj.g2267
12. Porco TC, Gebre T, Ayele B, et al. Effect of Mass Distribution of Azithromycin for Trachoma Control on Overall Mortality in Ethiopian Children A Randomized Trial. *Jama-Journal of the American Medical Association* 2009;302(9):962-68.
13. Amza A, Kadri B, Nassirou B, et al. A cluster-randomized controlled trial evaluating the effects of mass azithromycin treatment on growth and nutrition in Niger. *Am J Trop Med Hyg* 2013;88(1):138-43. doi: 10.4269/ajtmh.2012.12-0284
14. Amadi B, Mwiya M, Musuku J, et al. Effect of nitazoxanide on morbidity and mortality in Zambian children with cryptosporidiosis: a randomised controlled trial. *Lancet* 2002;360(9343):1375-80. doi: 10.1016/S0140-6736(02)11401-2
15. Shi H, Enriquez A, Rapadas M, et al. NAD Deficiency, Congenital Malformations, and Niacin Supplementation. *N Engl J Med* 2017;377(6):544-52. doi: 10.1056/NEJMoa1616361
16. Chen C, Mitchell NJ, Gratz J, et al. Fumonisin, Not Aflatoxin, is Associated with Growth Impairment in Tanzanian Children. *Environmental International* 2018;in press
17. Le Floc'h N, Otten W, Merlot E. Tryptophan metabolism, from nutrition to potential therapeutic applications. *Amino Acids* 2011;41(5):1195-205. doi: 10.1007/s00726-010-0752-7
18. Perlot T, Penninger JM. ACE2 - from the renin-angiotensin system to gut microbiota and malnutrition. *Microbes Infect* 2013;15(13):866-73. doi: 10.1016/j.micinf.2013.08.003
19. Hashimoto T, Perlot T, Rehman A, et al. ACE2 links amino acid malnutrition to microbial ecology and intestinal inflammation. *Nature* 2012;487(7408):477-81. doi: 10.1038/nature11228 [published Online First: 2012/07/25]
20. Wolf AM, Wolf D, Rumpold H, et al. Overexpression of indoleamine 2,3-dioxygenase in human inflammatory bowel disease. *Clin Immunol* 2004;113(1):47-55. doi: 10.1016/j.clim.2004.05.004
21. Gupta NK, Thaker AI, Kanuri N, et al. Serum analysis of tryptophan catabolism pathway: correlation with Crohn's disease activity. *Inflamm Bowel Dis* 2012;18(7):1214-20. doi: 10.1002/ibd.21849 [published Online First: 2011/08/05]
22. Favre D, Mold J, Hunt PW, et al. Tryptophan catabolism by indoleamine 2,3-dioxygenase 1 alters the balance of TH17 to regulatory T cells in HIV disease. *Sci Transl Med* 2010;2(32):32ra36. doi: 10.1126/scitranslmed.3000632
23. Mayneris-Perxachs J, Lima AA, Guerrant RL, et al. Urinary N-methylnicotinamide and β-aminoisobutyric acid predict catch-up growth in undernourished Brazilian children. *Sci Rep* 2016;6:19780. doi: 10.1038/srep19780

For peer review only - http://bmjopen.bmj.com/site/about/guidelines.xhtml

24. Kosek MN, Mduma E, Kosek PS, et al. Plasma Tryptophan and the Kynurenine-Tryptophan Ratio are Associated with the Acquisition of Statural Growth Deficits and Oral Vaccine Underperformance in Populations with Environmental Enteropathy. *Am J Trop Med Hyg* 2016;95(4):928-37. doi: 10.4269/ajtmh.16-0037 [published Online First: 2016/08/08]
25. Allen LH, Hampel D, Shahab-Ferdows S, et al. Antiretroviral therapy provided to HIV-infected Malawian women in a randomized trial diminishes the positive effects of lipid-based nutrient supplements on breast-milk B vitamins. *Am J Clin Nutr* 2015;102(6):1468-74. doi: 10.3945/ajcn.114.105106
26. Obradović J, Yousafzai AK, Finch JE, et al. Maternal scaffolding and home stimulation: Key mediators of early intervention effects on children's cognitive development. *Dev Psychol* 2016;52(9):1409-21. doi: 10.1037/dev0000182 [published Online First: 2016/08/08]
27. Walker SP, Wachs TD, Grantham-McGregor S, et al. Child Development 1 Inequality in early childhood: risk and protective factors for early child development. *Lancet* 2011;378(9799):1325-38. doi: 10.1016/s0140-6736(11)60555-2
28. Rasheed MA, Yousafzai AK. The development and reliability of an observational tool for assessing mother-child interactions in field studies- experience from Pakistan. *Child Care Health Dev* 2015 doi: 10.1111/cch.12287
29. Gladstone M, Lancaster GA, Umar E, et al. The Malawi Developmental Assessment Tool (MDAT): the creation, validation, and reliability of a tool to assess child development in rural African settings. *PLoS Med* 2010;7(5):e1000273. doi: 10.1371/journal.pmed.1000273
30. Bayley N. Bayley Scales of Infant and Toddler Development. San Antonio, TX: The Psychological Corporation 2006.
31. Murray-Kolb LE, Rasmussen ZA, Scharf RJ, et al. The MAL-ED Cohort Study: Methods and Lessons Learned When Assessing Early Child Development and Caregiving Mediators in Infants and Young Children in 8 Low- and Middle-Income Countries. *Clinical Infectious Diseases* 2014;59:S261-S72. doi: 10.1093/cid/ciu437
32. Donowitz JR, Petri WA. Pediatric small intestine bacterial overgrowth in low-income countries. *Trends Mol Med* 2015;21(1):6-15. doi: 10.1016/j.molmed.2014.11.001 [published Online First: 2014/11/15]
33. Donowitz JR, Haque R, Kirkpatrick BD, et al. Small Intestine Bacterial Overgrowth and Environmental Enteropathy in Bangladeshi Children. *MBio* 2016;7(1):e02102-15. doi: 10.1128/mBio.02102-15 [published Online First: 2016/01/12]
34. Trammell SA, Schmidt MS, Weidemann BJ, et al. Nicotinamide riboside is uniquely and orally bioavailable in mice and humans. *Nat Commun* 2016;7:12948. doi: 10.1038/ncomms12948 [published Online First: 2016/10/10]
35. Buchanan AM, Fiorillo SP, Omondi MW, et al. Establishment of biochemistry reference values for healthy Tanzanian infants, children and adolescents in Kilimanjaro Region. *Trop Med Int Health* 2015;20(11):1569-77. doi: 10.1111/tmi.12580 [published Online First: 2015/08/25]
36. Coghlan RF, Oberdorf JA, Sienko S, et al. A degradation fragment of type X collagen is a real-time marker for bone growth velocity. *Science Translational Medicine* 2017
37. Inagaki T, Moschetta A, Lee YK, et al. Regulation of antibacterial defense in the small intestine by the nuclear bile acid receptor. *Proc Natl Acad Sci U S A* 2006;103(10):3920-5. doi: 10.1073/pnas.0509592103 [published Online First: 2006/02/10]
38. Claus SP, Swann JR. Nutrimetabonomics: Applications for Nutritional Sciences, with Specific Reference to Gut Microbial Interactions. In: Doyle MP, Klaenhammer TR, eds. Annual Review of Food Science and Technology, Vol 4. Palo Alto: Annual Reviews 2013:381-99.
39. Ghodsi M, Liu B, Pop M. DNACLUST: accurate and efficient clustering of phylogenetic marker genes. *BMC Bioinformatics* 2011;12:271. doi: 10.1186/1471-2105-12-271

For peer review only - http://bmjopen.bmj.com/site/about/guidelines.xhtml

40. Liu J, Gratz J, Amour C, et al. A laboratory-developed TaqMan Array Card for simultaneous detection of 19 enteropathogens. *J Clin Microbiol* 2013;51(2):472-80. doi: 10.1128/JCM.02658-12 [published Online First: 2012/11/21]
41. Liu J, Gratz J, Amour C, et al. Optimization of Quantitative PCR Methods for Enteropathogen Detection. *PLoS One* 2016;11(6):e0158199. doi: 10.1371/journal.pone.0158199 [published Online First: 2016/06/23]
42. Liu J, Platts-Mills JA, Juma J, et al. Use of quantitative molecular diagnostic methods to identify causes of diarrhoea in children: a reanalysis of the GEMS case-control study. *Lancet* 2016;388(10051):1291-301. doi: 10.1016/S0140-6736(16)31529-X
43. Clinical_and_Laboratory_Standards_Institute. Performance Standards for Antimicrobial Susceptibility Testing, 27 ed. Wayne, Pennsylvania: Clinical and Laboratory Standards Institute 2017.
44. Ikehara Y, Nishihara S, Yasutomi H, et al. Polymorphisms of two fucosyltransferase genes (Lewis and Secretor genes) involving type I Lewis antigens are associated with the presence of anti-Helicobacter pylori IgG antibody. *Cancer Epidemiol Biomarkers Prev* 2001;10(9):971-7.
45. McGovern DP, Jones MR, Taylor KD, et al. Fucosyltransferase 2 (FUT2) non-secretor status is associated with Crohn's disease. *Hum Mol Genet* 2010;19(17):3468-76. doi: 10.1093/hmg/ddq248
46. Marionneau S, Airaud F, Bovin NV, et al. Influence of the combined ABO, FUT2, and FUT3 polymorphism on susceptibility to Norwalk virus attachment. *J Infect Dis* 2005;192(6):1071-7. doi: 10.1086/432546 [published Online First: 2005/08/09]
47. Yusuf S, Lonn E, Pais P, et al. Blood-Pressure and Cholesterol Lowering in Persons without Cardiovascular Disease. *N Engl J Med* 2016;374(21):2032-43. doi: 10.1056/NEJMoa1600177
48. Locks LM, Manji KP, McDonald CM, et al. Effect of zinc and multivitamin supplementation on the growth of Tanzanian children aged 6-84 wk: a randomized, placebo-controlled, double-blind trial. *Am J Clin Nutr* 2016;103(3):910-8. doi: 10.3945/ajcn.115.120055
49. Soofi S, Cousens S, Iqbal SP, et al. Effect of provision of daily zinc and iron with several micronutrients on growth and morbidity among young children in Pakistan: a cluster-randomised trial. *Lancet* 2013;382(9886):29-40. doi: 10.1016/S0140-6736(13)60437-7

For peer review only

For peer review only - http://bmjopen.bmj.com/site/about/guidelines.xhtml

FIGURE HEADINGS

Figure 1: The Tryptophan-kynurenine-niacin pathway in the intestinal mucosa. Dietary tryptophan is metabolized to kynurenine by indoleamine 2,3-dioxygenase (IDO)—whose activity is increased in settings of inflammation. Adverse effects of elevated IDO activity relate to modulation of the immune response, decreased activity of mTOR in producing antimicrobial peptides, and decreased tryptophan for protein synthesis. Intervention with nicotinamide ameliorates some of these effects in preclinical models; in the current study, we hypothesize that intervention with nicotinamide will increase linear growth via normalization of the tryptophan-kynurenine-niacin pathway and restored enterocyte activity. Both nicotinic acid and nicotinamide are considered dietary forms of niacin. Abbreviations: IDO = indoleamine 2,3-dioxygenase; NAD = nicotinamide adenine dinucleotide.

Figure 2: Taqman Array Card (TAC) schematic. Burden of intestinal pathogens will be assessed in stool samples from participants at multiple time points using TAC.

For peer review only

Table 1: Timing of interventions and measurements.

	Preg.	Birth-	Month of life													
		2 wks	1	2	3	4	5	6	7	8	9	10	11	12	15	18
RECRUITMENT, CONSENT	X	X														
ENROLLMENT		X														
INTERVENTIONS																
A. Nicotinamide intervention																
Nicotinamide (or placebo) to mother: daily 0-6 mo			X	X	X	X	X	X								
Nicotinamide (or placebo) to child: daily 6-12 mo									X	X	X	X	X	X	X	X
B. Antimicrobial intervention																
Azithromycin single dose to child								X			X			X	X	
Nitazoxanide 3-day course to child														X	X	
MEASUREMENTS																
A. Mother																
Weight: Month 11													X			
Height: Month 11													X			
Blood								X								
Stool								X								
Saliva (FUT2 secretor status)									X					X		
B. Child																
1. Anthropometry																
Weight		X	X	X	X	X	X	X	X	X	X	X	X	X	X	X
Length, head circumference, mid-upper arm circ.		X			X			X			X			X	X	X
2. Questionnaire																
Education, environment, socioeconomic			X													
Illness, treatment, breast feeding, food security			X	X	X	X	X	X	X	X	X	X	X	X	X	X
3. Breast milk: Months 1 and 5																
Tryptophn, niacin, nicotinamide, vitamers			X				X									
Vitamins			X				X									
Protein			X				X									
4. Blood																
Safety assessment (CMP, CBC in subset)				X						X						
Hemoglobin														X		X
IGF-1														X		X
CRP														X		X
Collagen X														X		X
Tryptophan metabolites														X		X
Metabolomics														X		X
5. Stool																
Microbiome								X[a]						X[b]		X
Taqman Array Cards								X[a]						X		X
Myeloperoxidase								X						X		X

For peer review only

For peer review only - http://bmjopen.bmj.com/site/about/guidelines.xhtml

6. Urine																
Metabolomics								X						X		X
7. Saliva																
FUT2 secretor status								X								
8. Breath																
H_2, methane (small intestinal bacterial overgrowth)								X						X		X
9. Developmental testing																
Observation of Maternal Child Interaction								X								
Malawi Developmental Assessment Tool																X
Bayley Scales of Development																X

For peer review only - http://bmjopen.bmj.com/site/about/guidelines.xhtml

Table 2: Primary and secondary outcome measures by study intervention.

Outcome	Timing (age of child in months)	Antimicrobial intervention		Nicotinamide intervention
Primary outcome				
LAZ	18	X		X
Secondary outcomes				
• Proportion of stunting (LAZ<-2)	18	X		X
• Weight-for-age (WAZ) and weight-for-length (WLZ) z-scores	18	X		X
• Metabolomic assay of gut microbial metabolism and alterations to tryptophan-niacin and other pathways	6, 12, 18	X		X
• Tryptophan:kynurenine ratio and other pathway metabolites in serum	12, 18	X		X
• IGF-1 at 12 and 18 months	12, 18	X		X
• High-sensitivity C-reactive protein at 12 and 18 months	12, 18	X		X
• Stool myeloperoxidase at months 6,12 and 18 months	6, 12, 18	X		X
• Prevalence of enteric pathogens	6, 6.5, 12, 12.5,18	X		X
• Change in microbiota (by traditional sequencing) (subset of participants)	6, 6.5, 12, 18	X		X
• Change in small intestinal bacterial overgrowth as assessed by exhaled increased hydrogen following ingestion of sugar (subset of participants)	6, 12, 18	X		X
• Difference in breast milk composition (nicotinamide, tryptophan, amino acids)	1, 5			X
• Symptomatic diarrhea		X		X
• Hospitalization and all-cause mortality	18	X		X
• Cognitive outcomes Malawi Development Assessment Tool (MDAT) assessment and the Bayley Scales of Infant and Toddler Development	18	X		X

For peer review only

For peer review only - http://bmjopen.bmj.com/site/about/guidelines.xhtml

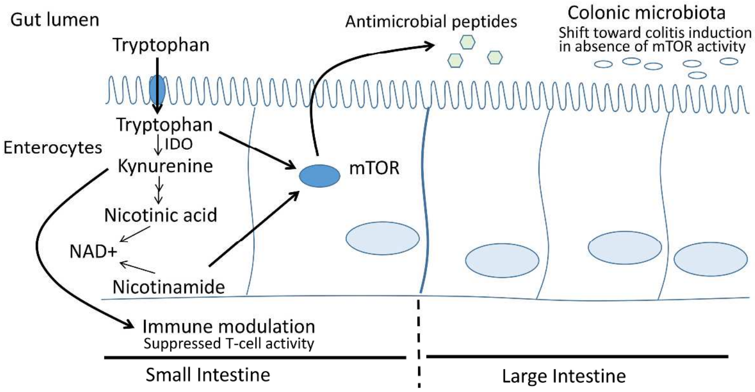

Figure 1: The Tryptophan-kynurenine-niacin pathway in the intestinal mucosa. Dietary tryptophan is metabolized to kynurenine by indoleamine 2,3-dioxygenase (IDO)—whose activity is increased in settings of inflammation. Adverse effects of elevated IDO activity relate to modulation of the immune response, decreased activity of mTOR in producing antimicrobial peptides, and decreased tryptophan for protein synthesis. Intervention with nicotinamide ameliorates some of these effects in preclinical models; in the current study, we hypothesize that intervention with nicotinamide will increase linear growth via normalization of the tryptophan-kynurenine-niacin pathway and restored enterocyte activity. Both nicotinic acid and nicotinamide are considered dietary forms of niacin. Abbreviations: IDO = indoleamine 2,3-dioxygenase; NAD = nicotinamide adenine dinucleotide.

338x190mm (300 x 300 DPI)

For peer review only - http://bmjopen.bmj.com/site/about/guidelines.xhtml

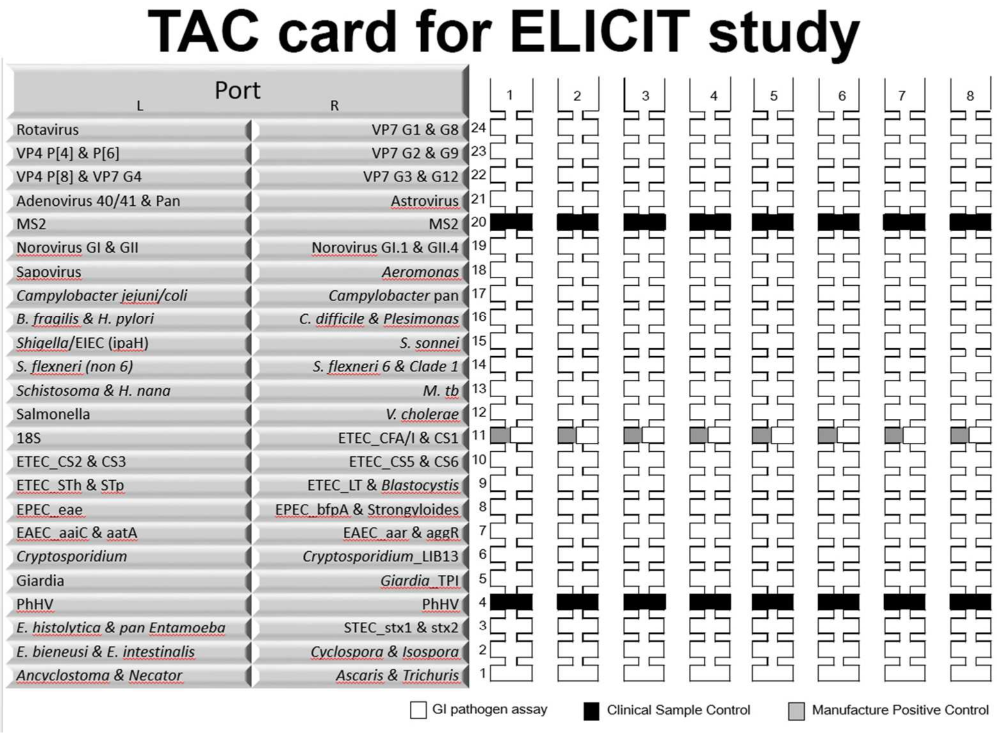

Figure 2: Taqman Array Card (TAC) schematic. Burden of intestinal pathogens will be assessed in stool samples from participants at multiple time points using TAC.

97x71mm (300 x 300 DPI)

SPIRIT

STANDARD PROTOCOL ITEMS: RECOMMENDATIONS FOR INTERVENTIONAL TRIALS

SPIRIT 2013 Checklist: Recommended items to address in a clinical trial protocol and related documents*

Section/item	Item No	Description	Addressed on page number
**Administrative information**			
Title	1	Descriptive title identifying the study design, population, interventions, and, if applicable, trial acronym	________1______
Trial registration	2a	Trial identifier and registry name. If not yet registered, name of intended registry	________2______
	2b	All items from the World Health Organization Trial Registration Data Set	______________
Protocol version	3	Date and version identifier	________3______
Funding	4	Sources and types of financial, material, and other support	________23_____
Roles and responsibilities	5a	Names, affiliations, and roles of protocol contributors	_____22-23_____
	5b	Name and contact information for the trial sponsor	________3______
	5c	Role of study sponsor and funders, if any, in study design; collection, management, analysis, and interpretation of data; writing of the report; and the decision to submit the report for publication, including whether they will have ultimate authority over any of these activities	_______23______
	5d	Composition, roles, and responsibilities of the coordinating centre, steering committee, endpoint adjudication committee, data management team, and other individuals or groups overseeing the trial, if applicable (see Item 21a for data monitoring committee)	_______NA______

For peer review only

For peer review only - http://bmjopen.bmj.com/site/about/guidelines.xhtml

Introduction

Background and rationale	6a	Description of research question and justification for undertaking the trial, including summary of relevant studies (published and unpublished) examining benefits and harms for each intervention	______5-7_____
	6b	Explanation for choice of comparators	______7_______
Objectives	7	Specific objectives or hypotheses	______7-8_____
Trial design	8	Description of trial design including type of trial (eg, parallel group, crossover, factorial, single group), allocation ratio, and framework (eg, superiority, equivalence, noninferiority, exploratory)	______7-8_____

Methods: Participants, interventions, and outcomes

Study setting	9	Description of study settings (eg, community clinic, academic hospital) and list of countries where data will be collected. Reference to where list of study sites can be obtained	______9_______
Eligibility criteria	10	Inclusion and exclusion criteria for participants. If applicable, eligibility criteria for study centres and individuals who will perform the interventions (eg, surgeons, psychotherapists)	______8_______
Interventions	11a	Interventions for each group with sufficient detail to allow replication, including how and when they will be administered	______9-10____
	11b	Criteria for discontinuing or modifying allocated interventions for a given trial participant (eg, drug dose change in response to harms, participant request, or improving/worsening disease)	_____19-20____
	11c	Strategies to improve adherence to intervention protocols, and any procedures for monitoring adherence (eg, drug tablet return, laboratory tests)	______10______
	11d	Relevant concomitant care and interventions that are permitted or prohibited during the trial	______10_______
Outcomes	12	Primary, secondary, and other outcomes, including the specific measurement variable (eg, systolic blood pressure), analysis metric (eg, change from baseline, final value, time to event), method of aggregation (eg, median, proportion), and time point for each outcome. Explanation of the clinical relevance of chosen efficacy and harm outcomes is strongly recommended	_18-19, Table 1__
Participant timeline	13	Time schedule of enrolment, interventions (including any run-ins and washouts), assessments, and visits for participants. A schematic diagram is highly recommended (see Figure)	_____9-10______

For peer review only - http://bmjopen.bmj.com/site/about/guidelines.xhtml

Sample size	14	Estimated number of participants needed to achieve study objectives and how it was determined, including clinical and statistical assumptions supporting any sample size calculations	______20_______
Recruitment	15	Strategies for achieving adequate participant enrolment to reach target sample size	______21_______

Methods: Assignment of interventions (for controlled trials)

Allocation:

Sequence generation	16a	Method of generating the allocation sequence (eg, computer-generated random numbers), and list of any factors for stratification. To reduce predictability of a random sequence, details of any planned restriction (eg, blocking) should be provided in a separate document that is unavailable to those who enrol participants or assign interventions	______9_______
Allocation concealment mechanism	16b	Mechanism of implementing the allocation sequence (eg, central telephone; sequentially numbered, opaque, sealed envelopes), describing any steps to conceal the sequence until interventions are assigned	______9_______
Implementation	16c	Who will generate the allocation sequence, who will enrol participants, and who will assign participants to interventions	______9_______
Blinding (masking)	17a	Who will be blinded after assignment to interventions (eg, trial participants, care providers, outcome assessors, data analysts), and how	______9-10_____
	17b	If blinded, circumstances under which unblinding is permissible, and procedure for revealing a participant's allocated intervention during the trial	______19-20____

Methods: Data collection, management, and analysis

Data collection methods	18a	Plans for assessment and collection of outcome, baseline, and other trial data, including any related processes to promote data quality (eg, duplicate measurements, training of assessors) and a description of study instruments (eg, questionnaires, laboratory tests) along with their reliability and validity, if known. Reference to where data collection forms can be found, if not in the protocol	____10-18______
	18b	Plans to promote participant retention and complete follow-up, including list of any outcome data to be collected for participants who discontinue or deviate from intervention protocols	_____9-10______

For peer review only - http://bmjopen.bmj.com/site/about/guidelines.xhtml

Data management	19	Plans for data entry, coding, security, and storage, including any related processes to promote data quality (eg, double data entry; range checks for data values). Reference to where details of data management procedures can be found, if not in the protocol	_____18________
Statistical methods	20a	Statistical methods for analysing primary and secondary outcomes. Reference to where other details of the statistical analysis plan can be found, if not in the protocol	_____18-19_____
	20b	Methods for any additional analyses (eg, subgroup and adjusted analyses)	_____19_______
	20c	Definition of analysis population relating to protocol non-adherence (eg, as randomised analysis), and any statistical methods to handle missing data (eg, multiple imputation)	_____19_______
**Methods: Monitoring**			
Data monitoring	21a	Composition of data monitoring committee (DMC); summary of its role and reporting structure; statement of whether it is independent from the sponsor and competing interests; and reference to where further details about its charter can be found, if not in the protocol. Alternatively, an explanation of why a DMC is not needed	_____19-20_____
	21b	Description of any interim analyses and stopping guidelines, including who will have access to these interim results and make the final decision to terminate the trial	_____19-20_____
Harms	22	Plans for collecting, assessing, reporting, and managing solicited and spontaneously reported adverse events and other unintended effects of trial interventions or trial conduct	_____19-20_____
Auditing	23	Frequency and procedures for auditing trial conduct, if any, and whether the process will be independent from investigators and the sponsor	______18_______
**Ethics and dissemination**			
Research ethics approval	24	Plans for seeking research ethics committee/institutional review board (REC/IRB) approval	______22_______
Protocol amendments	25	Plans for communicating important protocol modifications (eg, changes to eligibility criteria, outcomes, analyses) to relevant parties (eg, investigators, REC/IRBs, trial participants, trial registries, journals, regulators)	______22_______

For peer review only - http://bmjopen.bmj.com/site/about/guidelines.xhtml

Consent or assent	26a	Who will obtain informed consent or assent from potential trial participants or authorised surrogates, and how (see Item 32)	_____9________
	26b	Additional consent provisions for collection and use of participant data and biological specimens in ancillary studies, if applicable	_____9________
Confidentiality	27	How personal information about potential and enrolled participants will be collected, shared, and maintained in order to protect confidentiality before, during, and after the trial	_____18________
Declaration of interests	28	Financial and other competing interests for principal investigators for the overall trial and each study site	_____23________
Access to data	29	Statement of who will have access to the final trial dataset, and disclosure of contractual agreements that limit such access for investigators	_____18________
Ancillary and post-trial care	30	Provisions, if any, for ancillary and post-trial care, and for compensation to those who suffer harm from trial participation	_____19-20_____
Dissemination policy	31a	Plans for investigators and sponsor to communicate trial results to participants, healthcare professionals, the public, and other relevant groups (eg, via publication, reporting in results databases, or other data sharing arrangements), including any publication restrictions	________22______
	31b	Authorship eligibility guidelines and any intended use of professional writers	________22______
	31c	Plans, if any, for granting public access to the full protocol, participant-level dataset, and statistical code	________22______
**Appendices**			
Informed consent materials	32	Model consent form and other related documentation given to participants and authorised surrogates	__Supplement__
Biological specimens	33	Plans for collection, laboratory evaluation, and storage of biological specimens for genetic or molecular analysis in the current trial and for future use in ancillary studies, if applicable	____10-18______

*It is strongly recommended that this checklist be read in conjunction with the SPIRIT 2013 Explanation & Elaboration for important clarification on the items. Amendments to the protocol should be tracked and dated. The SPIRIT checklist is copyrighted by the SPIRIT Group under the Creative Commons "Attribution-NonCommercial-NoDerivs 3.0 Unported" license.

For peer review only - http://bmjopen.bmj.com/site/about/guidelines.xhtml
